# Monosulfonicpillar[5]arene: Synthesis, Characterization, and Complexation with Tetraphenylethene for Aggregation-Induced Emission

**DOI:** 10.1038/s41598-018-22446-y

**Published:** 2018-03-05

**Authors:** Xiao-Yu Jin, Nan Song, Xu Wang, Chun-Yu Wang, Yan Wang, Ying-Wei Yang

**Affiliations:** 1 International Joint Research Laboratory of Nano-Micro Architecture Chemistry (NMAC), College of Chemistry, Jilin University, 2699 Qianjin Street, Changchun, 130012 P. R. China; 2 State Key Laboratory of Supramolecular Structure and Materials, Institute of Theoretical Chemistry, Jilin University, 2699 Qianjin Street, Changchun, 130012 P. R. China

## Abstract

A pillar[5]arene derivative with a hydrophilic sulfonic group, *i.e*., monosulfonicpillar[5]arene (MSP5), has been successfully synthesized for the first time, which exhibited strong binding affinity towards alcohol analogs. Significantly, fluorescent supramolecular ensemble was fabricated from the supramolecular complexation of MSP5 and a neutral guest with tetraphenylethene core. Enhanced fluorescent emission of this system can be detected both in dilute solution and the solid state, and its temperature and competitive guest multi-responsive properties suggest its promising application as a chemical sensor towards alcohol analogs, ethylenediamine, and temperature variations.

## Introduction

Supramolecular macrocyclic compounds have been largely developed since the first generation of crown ethers till the fifth generation of pillar[n]arenes during the past decades^1–5^. Considering their superior properties, special structures and typical functions in supramolecular chemistry, remarkable attentions have been focused on the exploitation of their potentials and progress in molecular machines^6,7^, molecular recognition^8–11^, nanomaterials^12–16^, supramolecular polymers^17–21^, chemical sensors and detectors^22–25^, biological medicine^26–30^ and so on^31,32^. As a relatively new class of supramolecular macrocyclic hosts, pillar[n]arenes have attracted extensive attention owning to its unique properties, rigid structures, easy functionalization, etc.^33–35^. Monofunctionalized pillar[n]arenes, as one typical type of useful pillar[n]arene derivatives with unique substituent groups, possess variety of abilities such as molecular recognition and fluorescent detection, depending on their single modified functional groups and the different host-guest interactions^36–40^.

Aggregation-induced emission (AIE), entirely opposite to the aggregation-caused quenching (ACQ) effect of traditional fluorescent dyes^41–44^, was first reported by Tang and coworkers in 2001^45^, which paves a new way for the efficiency of fluorescent dyes in the solid state and the concentrated solution and breaks the limited applications of traditional fluorophores with ACQ properties. Restriction of intramolecular rotation (RIR) of AIE molecules was proven to be the well-known mechanism of their fluorescent enhancement in the aggregated state. Non-radiation energy dissipation channel from the excited state to ground state was blocked because of the hindrance of intramolecular steric interaction resulted from the impeded intramolecular rotations when the AIE molecules are assembled^46–48^. Tetraphenylethene (TPE) is a typical AIE molecule that has been widely investigated during the past two decades^49,50^. Several studies combining supramolecular approaches with typical fluorescent molecules with AIE properties to construct controllable fluorescent detectors or stimuli-responsive supramolecular materials have been reported^51–53^, especially using some pillar[n]arene derivatives as building blocks. This indicates that the combination of pillararenes and TPE is indeed an efficient way for the fabrication of novel smart optical devices to be applied in chemical sensors^51^, biological imaging^54,55^, and detection of pollutants and explosives^56,57^, among which the immediately selective detection of alcohol analogs is urgently required in the field of ecological environment and industrial development. The detection of alcohol analogs possesses special significance in traffic safety and medical emergency.

**Figure 1:**
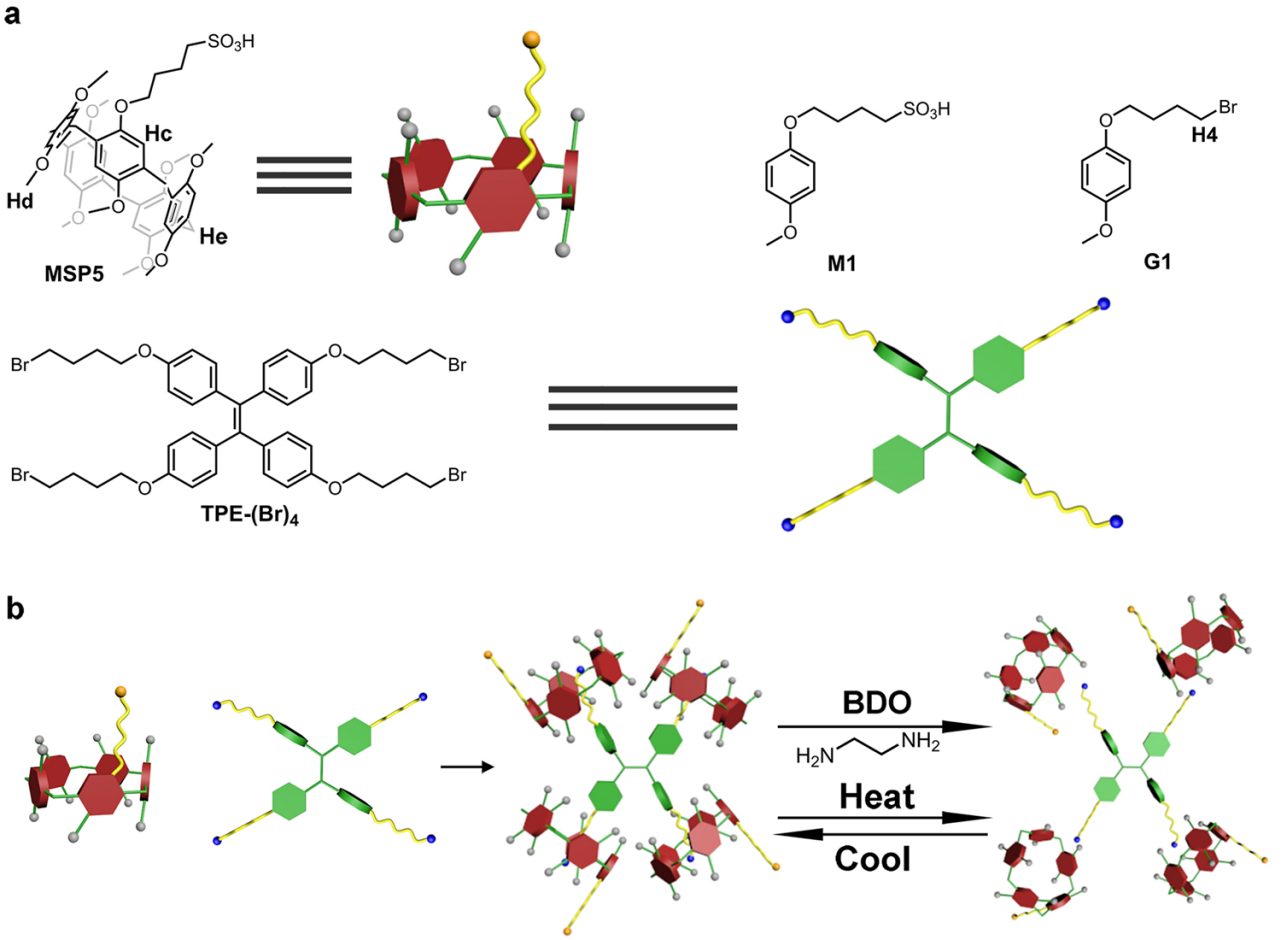
(**a**) Structural illustration of MSP5, TPE-(Br)_4_, M1 and G1; (**b**) Schematic representation of the interaction between MSP5 and TPE-(Br)_4_, and the stimuli-responsiveness of TPE-(Br)_4_⊂MSP5. BDO represents butanediol.

Herein, for the first time, we successfully synthesized sulfonic group-substituent monofunctionalized pillar[5]arene, i.e., monosulfonicpillar[5]arene (MSP5), (Fig. 1) in a good yield. A stable fluorescent complex between MSP5 and a guest TPE derivative, i.e., TPE-(Br)_4_, has also been designed and prepared *via* host-guest complexation, giving the credit to the hydrophilic group of MSP5 was not affected by pH of the solution. MSP5 also exhibited effective binding affinity towards alcohols *via* hydrogen bonds between hydroxyl of alcohols and sulfonic group of MSP5, making it possible to selectively detect alcohol analogs by destroying the complex of TPE-(Br)_4_⊂MSP5. Furthermore, the complex of MSP5 and TPE-(Br)_4_ can also serve as a temperature sensor and fluorescence probe for ethylenediamine.

## Results and Discussion

**Figure 2:**
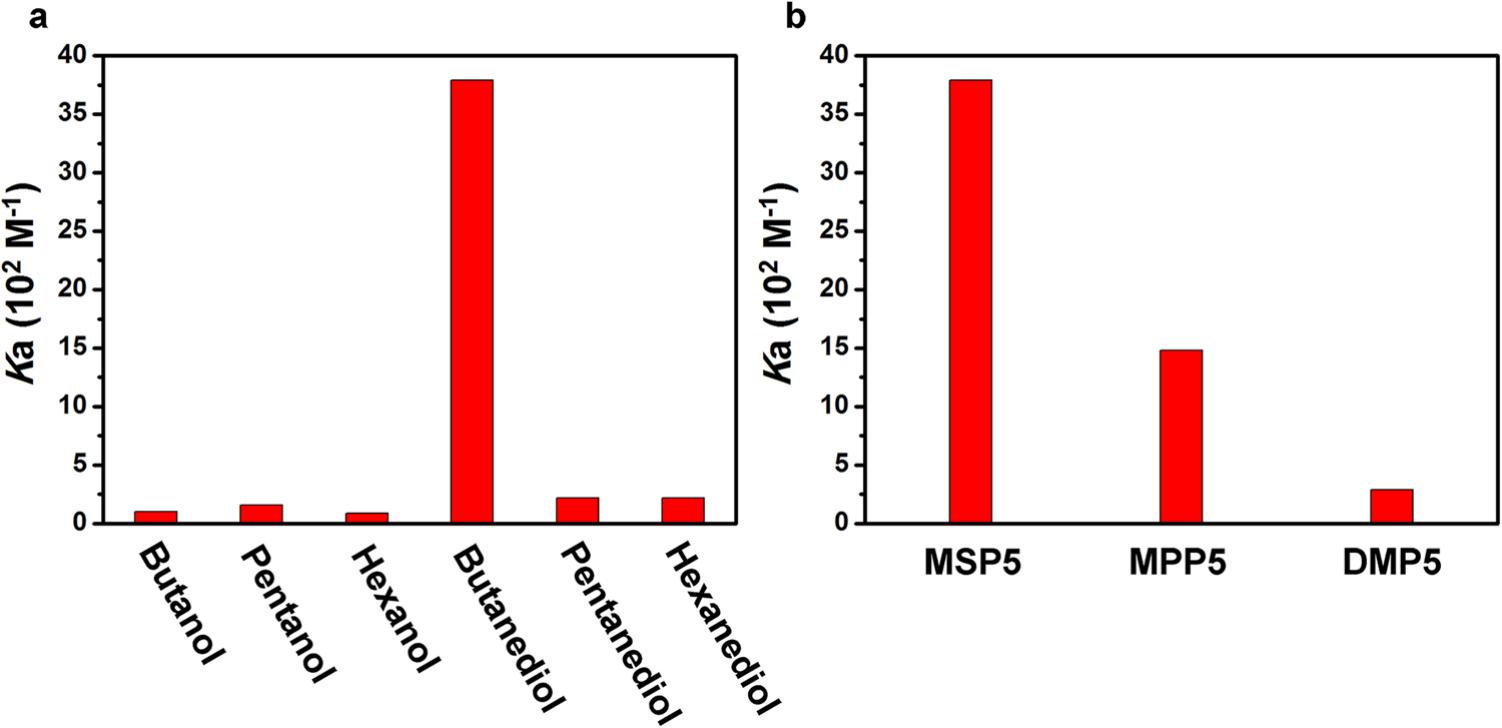
Association constants of (**a**) MSP5 with different alcohols; and (**b**) butanediol with different pillar[5]arene hosts including monosulfonicpillar[5]arene (MSP5), monophosphoryl copillar[5]arene (MPP5)^36^, and dimethoxypillar[5]arene (DMP5).

MSP5 was synthesized through the installation of a 1-butanesulfonic acid sodium onto pillar[5]arene *via* Williamson ether-type synthetic method. The structure of MSP5 was confirmed by ^1^H NMR, ^13^C NMR, HRMS, and FT-IR spectroscopy (Supplementary Fig. S5~S8). Considering that various monofunctionalized pillar[n]arenes possess the property of typical molecular recognition, six alcohol analogs were selected as guest molecules and the molecular recognition of MSP5 towards them was investigated *via* ^1^H NMR titration. As in Figs S34, S37, S40, S43, S46 and S49, when MSP5 was added into a chloroform solution of alcohols, the proton signal of H_a_ of alcohols showed an obvious upfield shift due to the shielding effect upon inclusion by pillararene cavity, indicating the host-guest interactions between MSP5 and alcohols. Nonlinear curve fitting method was employed to obtain the association constant (*K*a) between those alcohols and MSP5, respectively. MSP5 has the strongest binding affinity toward butanediol among other alcohols (Fig. 2a). Molar ratio plot based on the chemical shift changes of the protons of alcohols showed that all the stoichiometries of MSP5 and different alcohols are 1:1 (Supplementary Fig. S33). Interestingly, MSP5 possessing sulfonic entity exhibited much stronger binding affinity towards butanediol (Fig. 2b) as compared with monophosphoryl copillar[5]arene (MPP5)^36^ and dimethoxypillar[5]arene (DMP5), indicating its ability of selective recognition toward alcohol analogs particularly with an enlarged selectivity.

**Figure 3:**
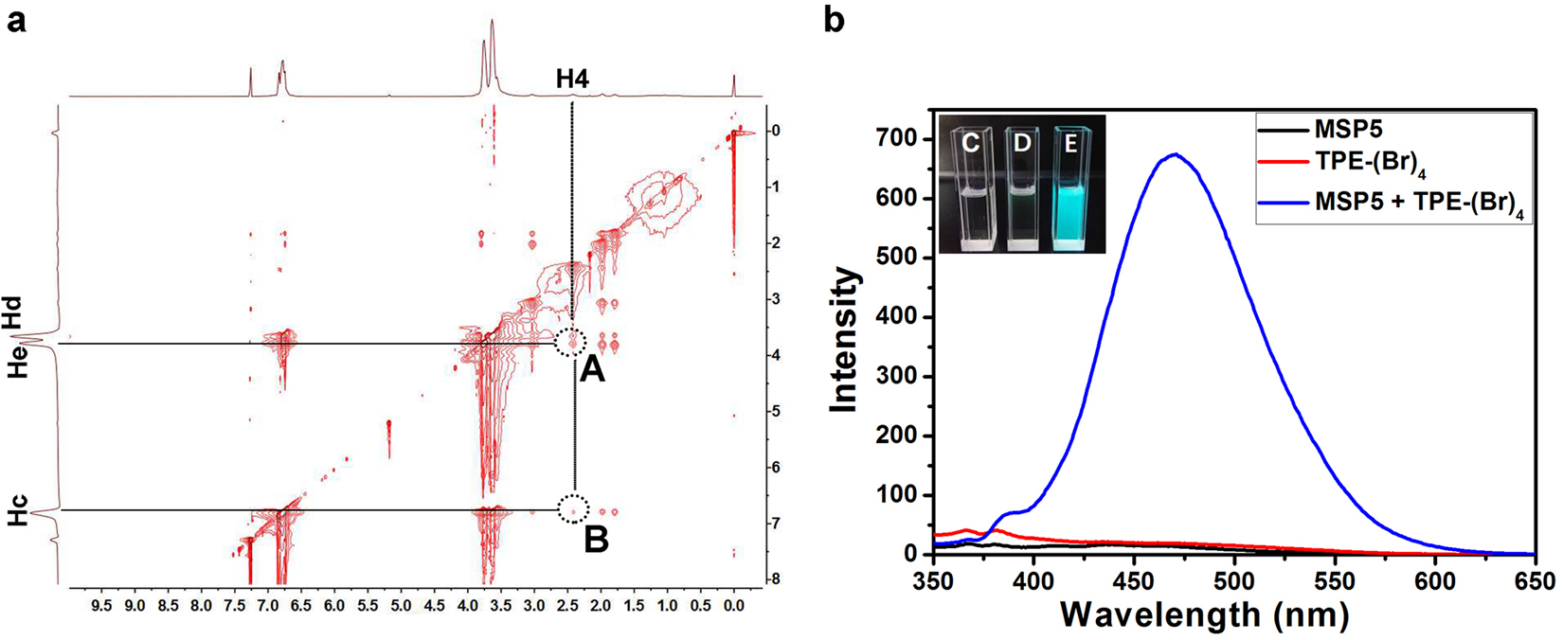
(**a**) NOESY NMR spectrum of MSP5 and G1 (see Fig. 1 for the assignments of NMR peaks); (**b**) The fluorescence spectra of MSP5, TPE-(Br)_4_ and TPE-(Br)_4_⊂MSP5 (2 × 10^−6^ M), the inset photographs show the corresponding fluorescence of each substrate upon excitation at 365 nm with a UV lamp at 298 K (concentration = 2 × 10^−4^ M), where C represents TPE-(Br)_4_, D represents MSP5 and E represents TPE-(Br)_4_⊂MSP5.

Based on the fact that MSP5 exhibited selective binding ability toward alcohols, we design a fluorescent complex *via* host-guest interaction between MSP5 and TPE-(Br)_4_ for molecular sensing and detection. We synthesized the TPE derivative with four binding arms that can be included in the cavity of MSP5, which can serve as a fluorescent indicator. A novel binary supramolecular-assembled fluorescent ensemble was constructed from MSP5 and TPE-(Br)_4_ (Fig. 1b). In order to investigate the host-guest properties between MSP5 and TPE-(Br)_4_, 1-(4-bromobutoxy)-4-methoxybenzene (G1) possessing the same binding site as TPE-(Br)_4_ was synthesized as a model compound. As shown in the Supplementary Fig. S52, when MSP5 was added into a chloroform solution of G1, the signals corresponding to the protons H_1_ and H_2_ on the alkyl chain shifted upfield, because these protons were located in the cavity of MSP5 and suffered from shielding effect. This provided a strong evidence for the interactions between MSP5 and G1. MSP5 forms a 1:1 complex with G1 as assessed by ^1^H NMR titration, and the *K*a of G1⊂MSP5 was calculated to be (1.08 ± 0.22) × 10^2^ M^−1^ in chloroform using nonlinear curve-fitting analysis (Supplementary Fig. S53). 2D NOESY NMR spectrum of MSP5 and G1 was also obtained for further investigation of the host-guest interaction between MSP5 and TPE-(Br)_4_. As shown in Fig. 3a, H_4_ was the proton on the guest while H_c_, H_d_, H_e_ were the protons on MSP5, the crosspeak A indicates that H_4_ is in close contact with H_d_, H_e_, and the crosspeak B indicates that H_4_ also interacts H_c_, suggesting that alkyl of G1 penetrated into the cavity of MSP5 to form a good inclusion complex. The proton NMR spectrum of MSP5 with TPE-(Br)_4_ in *d*-chloroform solution was also obtained and similar complexation-induced chemical shift changes were detected (Supplementary Fig. S58).

Strong emission in dilute solution was observed from the complex of TPE-(Br)_4_⊂MSP5, while non-fluorescent emission was surveyed from individual MSP5 and TPE-(Br)_4_ at the same concentration (Fig. 3b). Their host-guest fluorescence behaviors were investigated in detail and provided in the supplementary Fig. S59c. Upon increasing the concentration of MSP5, the fluorescence intensity of TPE-(Br)_4_ was gradually enhanced, which can be ascribed to the formation of host-guest inclusion complex that restricts the intramolecular rotation of phenyl rings of TPE-(Br)_4_. Besides, the fluorescence enhancement of TPE-(Br)_4_ induced by addition of MSP5 was clearly perceived by naked eyes (Fig. 3b-E), and strong cyan fluorescence can be visualized upon irradiation by a UV lamp with the wavelength of 365 nm, which was also supported by the above proposed mechanism.

**Figure 4:**
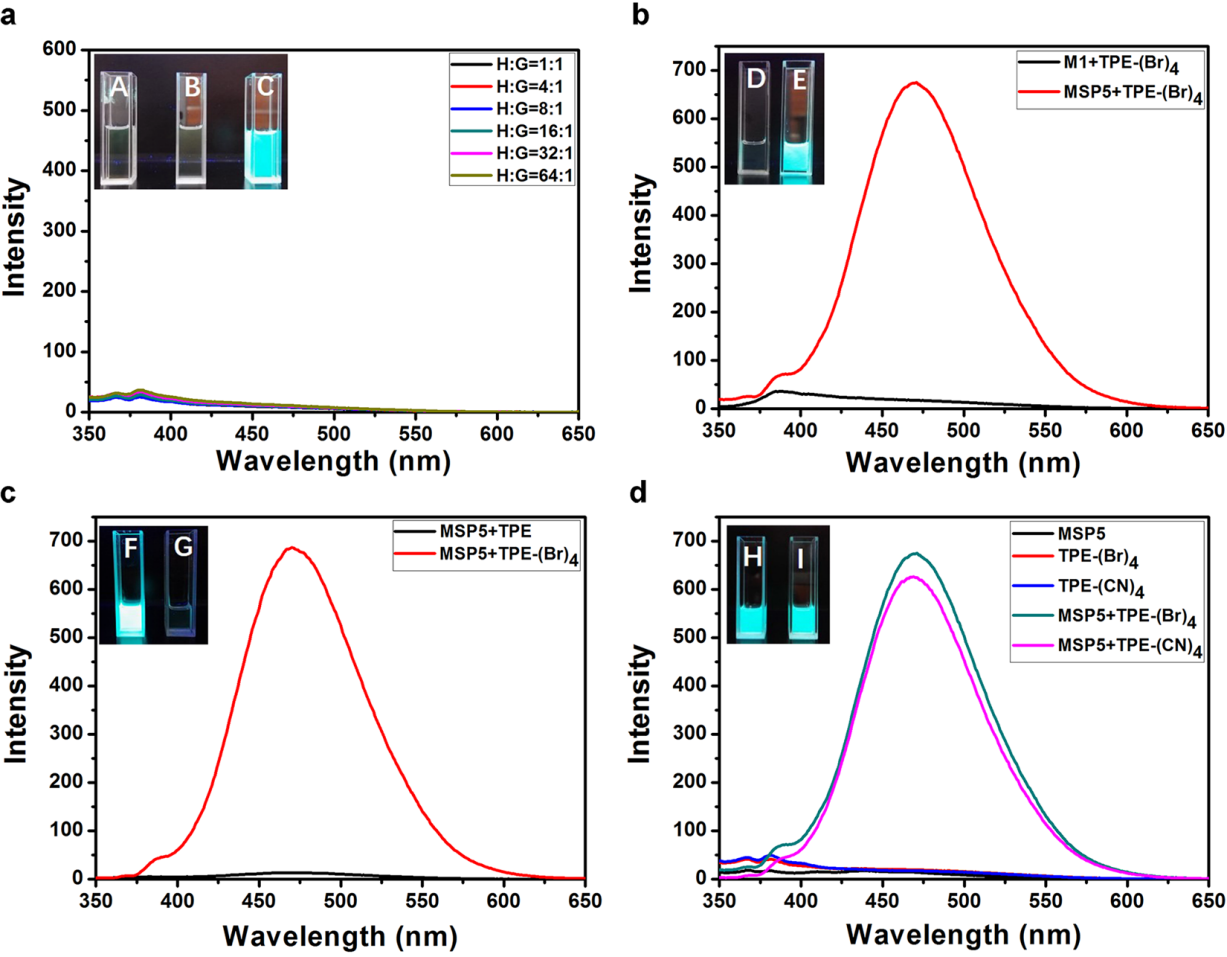
(**a**) The fluorescence changes of solutions upon addition of MCP5 to TPE-(Br)_4_ solution; The fluorescence spectra of (**b**) TPE-(Br)_4_⊂M1 and TPE-(Br)_4_⊂MSP5, (**c**) TPE-(Br)_4_⊂MSP5 and TPE⊂MSP5, (**d**) MSP5, TPE-(Br)_4_, TPE-(CN)_4_, TPE-(Br)_4_⊂MSP5 and TPE-(CN)_4_⊂MSP5 (concentration = 2 × 10^−6^ M); the insert photographs show the fluorescence of different host-guest complexes upon excitation at 365 nm using a UV lamp at 298 K (concentration = 2 × 10^−4^ M), (A) (E) (F) (H) TPE-(Br)_4_⊂DMP5, (B) TPE-(Br)_4_⊂MCP5, (C) TPE-(Br)_4_⊂MSP5, (D) TPE-(Br)_4_⊂M1, (G) TPE⊂MSP5 and (I) TPE-(CN)_4_⊂MSP5.

On the other hand, the fluorescence enhancement of TPE-(Br)_4_ upon addition of MSP5 confirmed the synergetic importance of host-guest interaction of pillararene and TPE guests and sulfonic functional group on pillararenes by a series of controlled experiments. The addition of MSP5 with a monosulfonic arm to form host-guest inclusion complex was proven to be the necessary condition for the fluorescent enhancement, consistent to RIR mechanism. TPE-(Br)_4_ and other two different host molecules, i.e., DMP5 and monocarboxylatopillar[5]arene (MCP5), were selected to investigate whether host-guest interaction itself will produce fluorescent enhancement of TPE-(Br)_4_. ^1^H NMR titration experiments provided *K*a between MCP5 and G1 (Supplementary Fig. S56), which is similar to that of G1⊂MCP5 and G1⊂MSP5. When DMP5 and MCP5 were added into TPE-(Br)_4_ chloroform solution gradually, the fluorescence of TPE-(Br)_4_ was almost unchanged (Fig. 4a), illustrating that host-guest interaction itself was unable to result in fluorescence enhancement. In addition, TPE and TPE-(CN)_4_ were also synthesized to investigate the effect of fluorescent molecules with different binding sites. When MSP5 mixed with TPE without binding affinity, the mixture was non-fluorescent (Fig. 4c), while strong fluorescence emission can be observed in the mixture of MSP5 and TPE-(CN)_4_ (Fig. 4d), for the reason of the host-guest complex between MSP5 and TPE-(CN)_4_^58^. Meanwhile, the fluorescent emission of TPE-(CN)_4_⊂MSP5 was similar to that of TPE-(Br)_4_⊂MSP5 (Fig. 4d), indicating that different functional groups on TPE with similar binding ability with pillararene had negligible influence on the fluorescence enhancement. Monomer of MSP5, i.e., 4-(4-methoxyphenoxy)butane-1-sulfonic acid (M1), was also synthesized to study the effect of sulfonic group on fluorescent enhancement of TPE-(Br)_4_. No fluorescence was observed when M1 mixed with TPE-(Br)_4_ (Fig. 4b), indicating that sulfonic group had no effect on the fluorescence of TPE-(Br)_4_.

Sulfonic group, as a hydrophilic entity, plays a key role in the complex system of TPE-(Br)_4_⊂MSP5 to maintain stable under different pH conditions, which was different from that of carboxylic acid group as the hydrophobic group in MCP5. We deduced that the hydrophobicity of the functional groups affected the fluorescence behaviors. Thus, three kinds of anionic monofunctional pillar[5]arenes and their sodium salts (sulfonic group, sulfonate group, carboxyl group, carboxylate group, phosphoric group, phosphate group) have been synthesized to investigate the role of sulfonic group in the fluorescent ensembles. The enhanced fluorescence of TPE-(Br)_4_⊂MSP5 can be detected under low concentration, and upon addition of MSP5, fluorescence enhanced gradually. Furthermore, the fluorescence of other five kinds of supramolecular ensembles has also been studied at the same conditions, and the results are shown in Supplementary Fig. S59. No obvious fluorescence enhancement of TPE-(Br)_4_⊂MCP5 was detected, while the fluorescence was largely enhanced in TPE-(Br)_4_⊂MSP5 and TPE-(Br)_4_⊂monosulfonatepillar[5]arene. Monophosphoricpillar[5]arene also induced weaker fluorescence enhancement. On the contrary, monophosphatepillar[5]arene induced remarkable fluorescent enhancement, same as MSP5 and monosulfonatepillar[5]arene. From the above results, we can further concluded that the water-soluble groups-substituents monofunctionalized pillar[5]arene can enhance the fluorescence of TPE-(Br)_4_ *via* host-guest inclusion. There was an obvious difference in p*Ka* of the substituent groups in pillar[5]arene derivatives: R-SO_3_H (1.6 in DMSO) < R-PO_3_H_2_ (2.59 and 8.19 in water/ethanol) < R-COOH (12.3 in DMSO)^59–61^, which indicates that sulfonic group exists in the form of acidic anion in chloroform solution. However, phosphoric has two p*Ka*, the strong acidic hydrogen will be ionized in chloroform, resulting in slight fluorescence enhancement of TPE-(Br)_4_⊂MSP5. The carboxylic acid group maintains un-ionized form in chloroform owning to the weak acidity, causing no fluorescence enhancement.

Scanning electron microscope (SEM), dynamic laser scattering (DLS), and DOSY NMR spectrum have been used to further investigate the fluorescence and self-assembled behaviors. The DOSY NMR spectrum showed that all the peaks correlated to the signals in the chemical shift dimensions are in a horizontal line (Supplementary Fig. S60), all proton signals of MSP5 and TPE-(Br)_4_ have the same diffusion coefficient (2.6 × 10^−9^ m^2^s^−1^), suggesting the host-guest interaction of TPE-(Br)_4_⊂MSP5. The solution of the host-guest complex exhibited obvious Tyndall effect (Supplementary Fig. S61e), indicating that the complex formed abundant colloid particles. SEM images and DLS data proved the aggregation of TPE-(Br)_4_⊂MSP5. All the above results illustrated that the TPE-(Br)_4_⊂MSP5 can self-assemble into nanoparticles with the average diameter of 16 nm (Supplementary Fig. S61c,d,f), while the individual host and guest are amorphous (Supplementary Fig. S61a,b).

We thus ascribed the fluorescence enhancement to the following reasons: (i) The host-guest interaction of TPE-(Br)_4_⊂MSP5 formed into pseudorotaxane, restricted the intramolecular rotation of phenyl rings of TPE-(Br)_4_ and blocked the nonradiative emission, leading to a strong fluorescence emission; (ii) The solubility of the host-guest complex (fluorescent nanoparticles) in chloroform was reduced due to the hydrophilic group, leading to the aggregation state; (iii) MSP5 can self-assemble with TPE-(Br)_4_ to construct organic fluorescent nanoparticles, reaching aggregation state and exhibiting strong emission.

**Figure 5:**
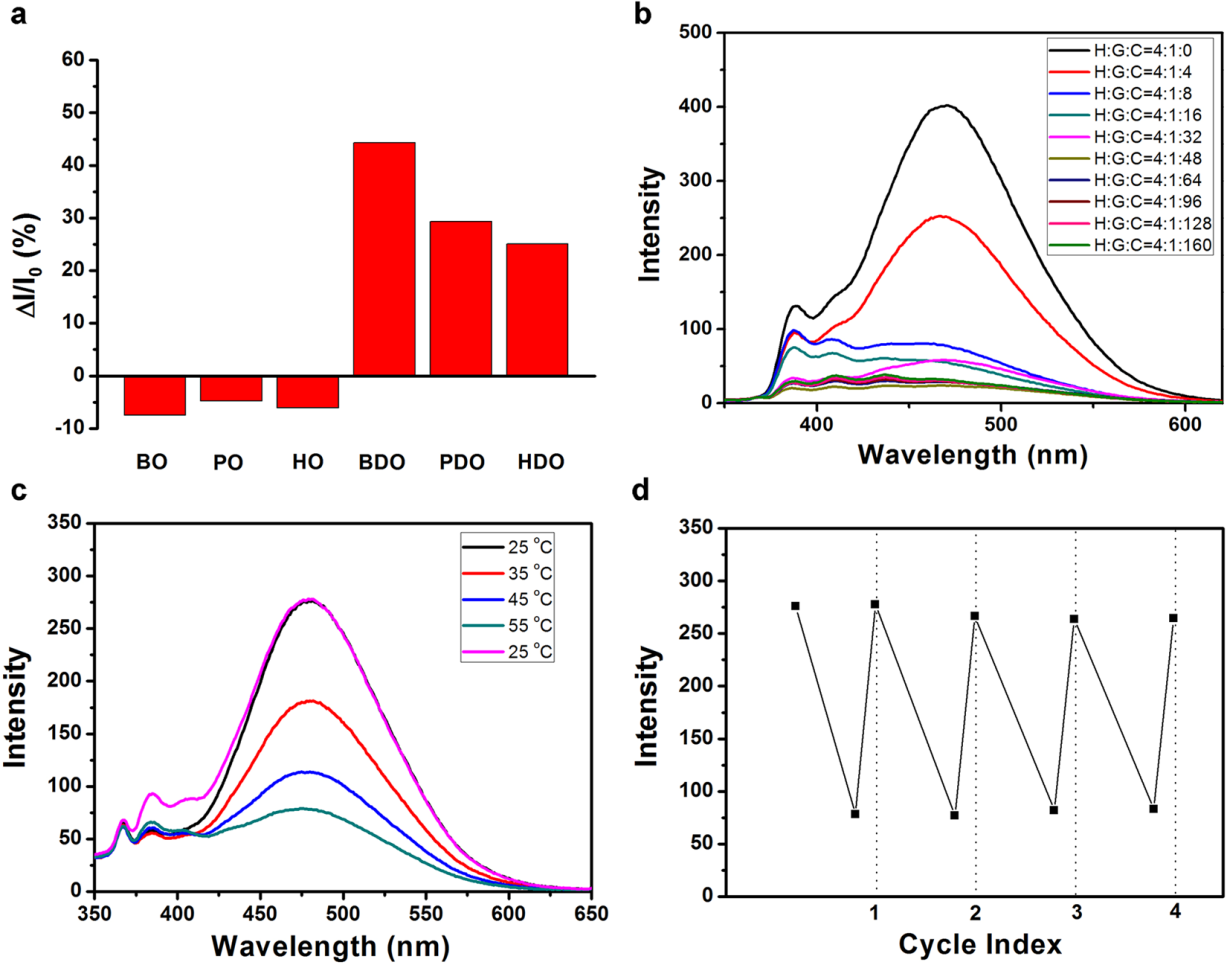
(**a**) The selectivity of butanediol (BDO) used fluorescence indicator (n-butanol = BO, n-pentanol = PO, n-hexanol = HO, pentanediol = PDO, hexanediol = HDO, concentration = 1 × 10^−4^ M); (**b**) the detection of ethylenediamine (concentration = 2 × 10^−6^ M, the amount of ethylenediamine is 8 × 10^−6^ M); (**c**) the temperature responsiveness of host-guest fluorescence composite (concentration = 1 × 10^−6^ M in THF); (**d**) the cycle performance of this fluorescence temperature sensor.

We successfully utilize the host-guest interaction property of pillar[5]arene and the AIE effect of TPE, prepared a binary complex system, where TPE was used as a fluorescence indicator for identifying butanediol effectively (Fig. 5a). Ethylenediamine is toxic, which would damage human bodies and environment seriously. On account of the much stronger binding affinity of MSP5 towards ethylenediamine than TPE-(Br)_4_ (Supplementary Fig. S62)^40^, the ensemble of TPE-(Br)_4_⊂MSP5 can be used to detect ethylenediamine sensitively and rapidly (Fig. 5b). In addition, this supramolecular assembly can also be applied as a temperature sensor, as the fluorescence intensity decreased gradually upon raising the temperature. The fluorescence intensity can revert to the initial intensity without wastage when temperature returned to the initial room temperature (Fig. 5c), indicating this temperature sensor has remarkable circulation performance and can be reused for many times (Fig. 5d).

## Conclusion

In summary, we synthesized MSP5 for the first time and employed it to construct a stable AIE-active binary complex system with TPE core via host-guest interaction and supramolecular self-assembly. Upon the formation of host-guest complex, the fluorescence emission of the complex was enhanced dramatically. The resulting pseudorotaxane-type structure restricted the intramolecular rotation of phenyl rings of the TPE-(Br)_4_ and blocked the nonradiative emission, finally resulting in strong fluorescence emission. We also investigated the molecular recognition ability of MSP5, and found that it can form stable complexes with alcohols. The MSP5 and TPE-(Br)_4_ can be used to fabricate supramolecular fluorescence composite through supramolecular self-assembly. This new fluorescence complex system possesses multi-stimuli responsive properties, and can selectively recognize butanediol among several similar alcohols. It also can act as a fluorescence probe to detect toxic substance ethanediamine and act as a temperature sensor. We envision that combining the AIE effect of TPE with the host-guest property of functional macrocycles may lead to many potential applications of pseudorotaxanes in sensors, cell imaging, controlled optical materials and smart materials.

## Experimental Section

### Methods

All reagents were commercially available and used without further purification. TPE-(Br)_4_, G1, M1 and MCP5 were synthesized according to a published literature procedure (See the Supporting Information for details)^20,37,50,62^. ^1^H NMR spectra were collected on a Bruker AVANCE III 300 MHz NMR spectrometer. ^13^C NMR, 2D NOESY NMR and DOSY NMR spectra were recorded on a Bruker AVANCE III 500 MHz NMR spectrometer. High-resolution electrospray ionization mass spectra (HRESI-MS) were obtained on a Bruker 7-Tesla FT-ICR mass spectrometer equipped with an electrospray source. Mass spectra were recorded on Bruker Daltonics Autoflex Speed Series: High-Performance MALDI-TOF Systems. FT-IR spectra were recorded on a Vertex 80 V spectrometer. Scanning electron microscope (SEM) images were obtained on a HITACHI-SU8082 instrument. The fluorescence experiments were conducted on a RF-5301 spectrofluorophotometer (Shimadzu Corporation, Japan). To determine the stoichiometry and association constants of alcohols⊂MSP5, ^1^H NMR titration was performed. By a nonlinear curve-fitting method, the association constants between the guests and host were calculated. Through a molar ratio plot, the stoichiometry was determined (see supporting information for details).

### Synthesis of MSP5

MonohydroxyDMP[5] (500 mg, 0.68 mmol, see supporting information for details) and NaOH (60 mg, 1.5 mmol) were added into 25 mL THF in a 50 mL flask, the mixture was stirred at room temperature for 1 h. Then, 1,4-butylenesulfone (0.15 mL) was added into the mixture. The mixture was stirred at 40 °C for 24 h. The crude product was recrystallized with dichloromethane/n-hexane and washed with water. After dryness, monosulfonatepillar[5]arene sodium salt was obtained as a yellow powder (400 mg, 64%). ^1^H NMR (300 MHz, CDCl_3_, 25 °C), δ (ppm): 6.73 (m, 10H), 3.46~3.77 (m, 39H), 3.02 (t, 2H), 1.79~1.96 (m, 4H). ^13^C NMR (126 MHz, CDCl_3_, 25 °C) δ (ppm): 150.73, 149.82, 128.22, 114.11, 68.31, 55.80, 50.97, 29.76, 28.82, 21.35. HRESIMS is shown in Fig. S11: *m*/*z* 871.3468 [M−Na]^−^ (100%). Then the sodium salt (200 mg) was dispersed in water and stirred with hydrochloric acid at room temperature for 12 h. After the solvent was removed, the obtain solid was purified by column chromatograph with dichloromethane/methanol (1:10 v/v) to get the final product of MSP5. Yellow powder: 140 mg, 72%. ^1^H NMR (300 MHz, CDCl_3_, 25 °C), δ (ppm): 6.72 (m, 10H), 3.55~3.76 (m, 39H), 3.03 (t, 2H), 1.80~1.99 (m, 4H). ^13^C NMR (125 MHz, CDCl_3_, 25 °C) δ (ppm): 150.77, 128.30, 114.17, 68.28, 55.82, 51.01, 29.72, 28.72, 21.38. HRESIMS is shown in Fig. S7: *m*/*z* 871.3373 [M−H]^−^ (100%).

### Synthesis of TPE-(CN)_4_

Tetra-hydroxyl-TPE (120 mg) and K_2_CO_3_ (144 mg) were added into 20 mL CH_3_CN. The mixture was stirred at room temperature for 30 min. Then 5-bromovaleronitrile was added into the above solution, reacted under reflux for 24 h. After the solvent was removed, the obtain solid was purified by column chromatograph with petroleum ether/dichloromethane/ethyl acetate (15:15:1 v/v) to get the final product. Pale yellow powder, 75 mg, 34%. ^1^H NMR (300 MHz, CDCl_3_, 25 °C), δ (ppm): 6.90 (d, *J* = 9Hz, 8H), 6.60 (d, *J* = 9 Hz, 8H), 3.93 (m, 8H), 2.44 (m, *J* = 6 Hz, 8H), 1.90 ${({\rm{m}},{\rm{16H}})}_{.}$ ^13^C NMR (126 MHz, CDCl_3_, 25 °C) δ (ppm): 157.04, 137.11, 132.66, 119.62, 113.62, 66.56, 28.31, 22.59, 17.13. MALDI-TOF MS is shown in Fig. S17: *m*/*z* 720.2358 [M] (100%).

### Synthesis of monocarboxylatepillar[5]arene sodium salt

Monoesterpillar[5]arene (50 mg, 0.06 mmol) was added into sodium hydroxide aqueous solution (2.5 mL, 20%) and THF (4.2 mL). The mixture was stirred at 85 °C for 24 h. After solvent was removed, the obtained solid was recrystallized with dichloromethane/n-hexane. White powder: 40 mg, 82%. ^1^H NMR (300 MHz, CDCl_3_, 25 °C), δ (ppm): 6.67~6.74 (m, 10H), 4.18 (t, 2H), 3.59~3.76 (m, 37H). ^13^C NMR (126 MHz, CDCl_3_, 25 °C) δ (ppm): 175.86, 150.88, 128.45, 114.13, 71.74, 55.95, 29.81. HRESIMS is shown in Fig. S21: *m*/*z* 871.3468 [M−Na]^−^ (100%).

### Synthesis of monophosphite-DMP[5]

Copillar[5]arene 1 (0.3 g, 0.36 mmol) was added into triethyl phosphite (1.2 mL). The mixture was stirred at 150 °C for 24 h. The crude product was concentrated and subjected to column chromatograph with petroleum ether/ethyl acetate (1:20 v/v) to get the final product. Pale yellow powder, 260 mg, 78%. ^1^H NMR (300 MHz, CDCl_3_, 25 °C), δ (ppm): 6.72~6.77 (m, 10H), 4.09 (m, 4H), 3.85 (m, 3H), 3.77 (m, 10H), 3.64 (m, 27H), 1.85~1.89 (m, 4H), 1.70 (m, 2H), 1.29 (t, 6H). ^13^C NMR (126 MHz, CDCl_3_, 25 °C) δ (ppm): 150.75, 149.87, 128.32, 114.95, 114.00, 67.74, 61.54, 55.87, 30.68, 29.82, 19.57, 16.45. MALDI-TOF MS is shown in Fig. S26: *m*/*z* 930.4110 [M + H]^+^, 953.2957 [M + H + Na]^2+^, 969.3292 [M + H + K]^2+^.

### Synthesis of Monophosphoricpillar[5]arene

Monophosphite-DMP[5] (0.28 g, 0.3 mmol, see supporting information for details) was added into 10 mL CH_2_Cl_2_. Then TMSBr (0.4 mL) was added into the above solution. The mixture was stirred at room temperature for 24 h. The crude product was concentrated and recrystallized to give the final product. White powder: 204 mg, 78%. ^1^H NMR (300 MHz, CDCl_3_, 25 °C), δ (ppm): 6.75 (m, 10H), 3.62~3.76 (m, 39H), 1.67 (m, 4H), 1.02 (t, 2H). ^13^C NMR (126 MHz, CDCl_3_, 25 °C) δ (ppm): 150.74, 128.26, 113.97, 67.80, 55.78, 52.55, 30.61, 29.60, 19.30. HRESIMS is shown in Fig. S29: *m*/*z* 871.3453 [M−H]^−^ (100%).

### Synthesis of Monophosphatepillar[5]arene sodium salt

Monophosphoricpillar[5]arene (50 mg) was dissolved in 5 mL THF, then added into 5 mL NaOH aqueous (2 M/L) dropwise, which was allowed to react at room temperature for 12 h. Then THF solvent was evaporated and the solid was collected as a pale yellow powder: 30 mg, 59%. ^1^H NMR (300 MHz, CDCl_3_, 25 °C), δ (ppm): 6.69 (m, 10H), 3.53~3.75 (m, 39H), 1.74 (m, 4H), 1.27 (m, 2H). ^13^C NMR (126 MHz, CDCl_3_, 25 °C) δ (ppm): 150.78, 128.20, 114.21, 67.97, 61.54, 55.76, 29.71, 25.60, 14.11. HRESIMS is shown in Fig. S32: *m*/*z* 871.3604 [M−H]^−^ (100%).

## Electronic supplementary material

Supplementary Information
