# Supplementary material for: Monosulfonicpillar[5]arene: Synthesis, Characterization, and Complexation with Tetraphenylethene for Aggregation-Induced Emission
Source: Sci Rep. 2018 Mar 5;8:4035. doi: 10.1038/s41598-018-22446-y (PMC5838235; doi:10.1038/s41598-018-22446-y)
Supplement: Supplementary file 1 — Supplementary Information [file 41598_2018_22446_MOESM1_ESM.pdf]

Supporting Information

Monosulfonicpillar[5]arene: Synthesis, Characterization, and Complexation with Tetraphenylethene for Aggregation-Induced Emission in Dilute Solution

Xiao-Yu Jin[1], Nan Song[1], Xu Wang[1], Chun-Yu Wang[2], Yan Wang[1,*], and Ying-Wei Yang[1,*]

[1]International Joint Research Laboratory of Nano-Micro Architecture Chemistry (NMAC), College of Chemistry, Jilin University, 2699 Qianjin Street, Changchun 130012, P. R. China.

[2]State Key Laboratory of Supramolecular Structure and Materials, Institute of Theoretical Chemistry, Jilin University, 2699 Qianjin Street, Changchun 130012, P. R. China.

[†]E-mail: ywyang@jlu.edu.cn (Y.-W.Y), wangy2011@jlu.edu.cn (Y. W.)

1. Materials and methods

2. Synthesis of TPE-$(Br)_4$

3. Synthesis of MSP5

4. Synthesis of M1

5. Synthesis of G1

6. Synthesis of TPE-$(CN)_4$

7. Synthesis of MCP5

8. Synthesis of Monophosphoricpillar[5]arene

9. Stoichiometry and association constant determination for alcohols⊂MSP5 in $CHCl_3$

10. Association constant determination for G1⊂MSP5 in $CHCl_3$

11. Association constant determination for G1⊂MCP5 in $CHCl_3$

12. Host-guest interaction for TPE-$(Br)_4$⊂MSP5 in $CHCl_3$

13. Host-guest interaction for TPE-$(Br)_4$⊂pillararene derivatives in $CHCl_3$

14. DOSY NMR spectrum of TPE-$(Br)_4$⊂MSP5

15. SEM images of MSP5 and TPE-$(Br)_4$

16. NMR spectra of MSP5 and ethylenediamine

17. Solid state fluorescence image and spectrum of host-guest complex

1. Materials and methods

All reagents were commercially available and used without further purification. TPE-$(Br)_4$, G1, M1 and MCP5 were synthesized according to a published literature procedure (See the Supporting Information for details).[S1-S4]

2. Synthesis of TPE-$(Br)_4$[S1]

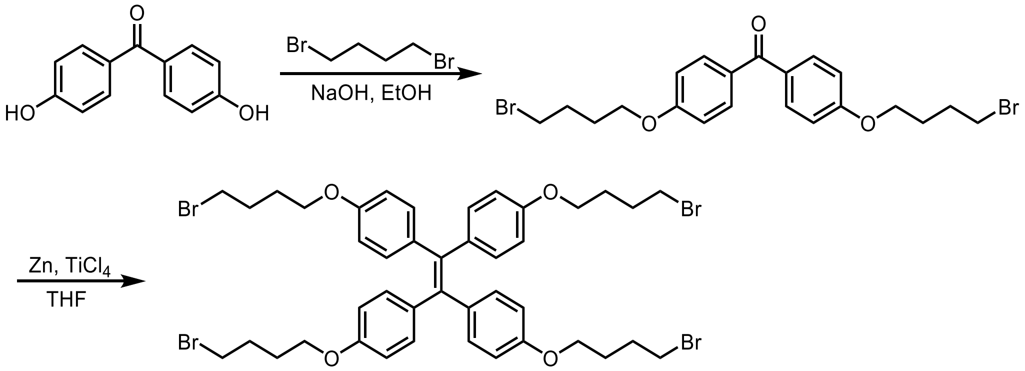

Bis(4-hydroxyphenyl)methanone (5 g, 23.4 mmol) and NaOH (3.7 g, 56 mmol) were added into a 250 mL CH_3CH_2OH in a 500 mL flask. Then 1,4-dibromobutane (16.7 mL) was added into the above solution and reacted under reflux for 12 h. The mixture was filtered and washed with CH_3CH_2OH and water to give a white powder, which is bis(4-(4-bromobutoxy)phenyl)methanone: 6.7g, 59 %. ^{1}H NMR (300 MHz, $CDCl_3$, 25 °C), δ (ppm): 7.76 (d, J = 9 Hz, 4 H), 6.93 (d, J = 9 Hz, 4 H), 4.08 (t, J = 6 Hz, 4 H), 3.51 (t, J = 6 Hz,4 H), 1.98~2.10 (m, 8 H).

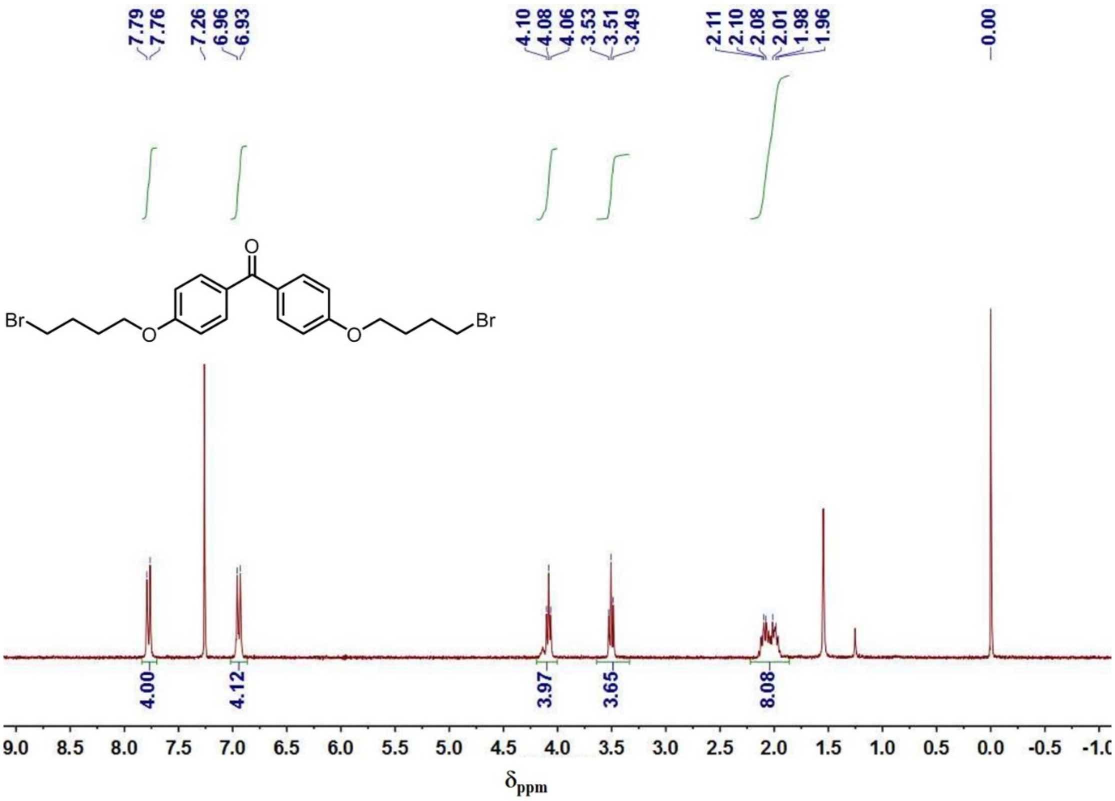

Figure S1. ^{1}H NMR spectrum (300 MHz, $CDCl_3$, 298 K) of bis(4-(4-bromobutoxy)phenyl)methanone.

Znic (1.7 g) and titaniumtetrachloride (1.7 mL) were added into 100 mL dry tetrahydrofuran at 0 °C and reacted at 0 °C for 30 min. Then bis(4-(4-bromobutoxy)phenyl)methanone (2.4 g) tetrahydrofuran solution was added into above solution and the mixture was reacted under reflux for 3 h. Then, potassium carbonate (K_2CO_3) solution was added into the mixture to quench the reaction. After the solvent was removed, the obtain solid was purified by column chromatograph with a eluent of petroleum ether/dichloromethane (2:1 v/v) to give the final product of TPE-$(Br)_4$. White powder: 550 mg, 24 %. ^{1}H NMR (300 MHz, $CDCl_3$, 25 °C), δ (ppm): 6.93 (d, J = 9 Hz, 8 H), 6.63 (d, J = 9 Hz, 8 H), 3.92 (t, J = 6 Hz, 8 H), 3.48 (t, J = 6 Hz, 8 H), 1.90~2.08 (m, 16 H).

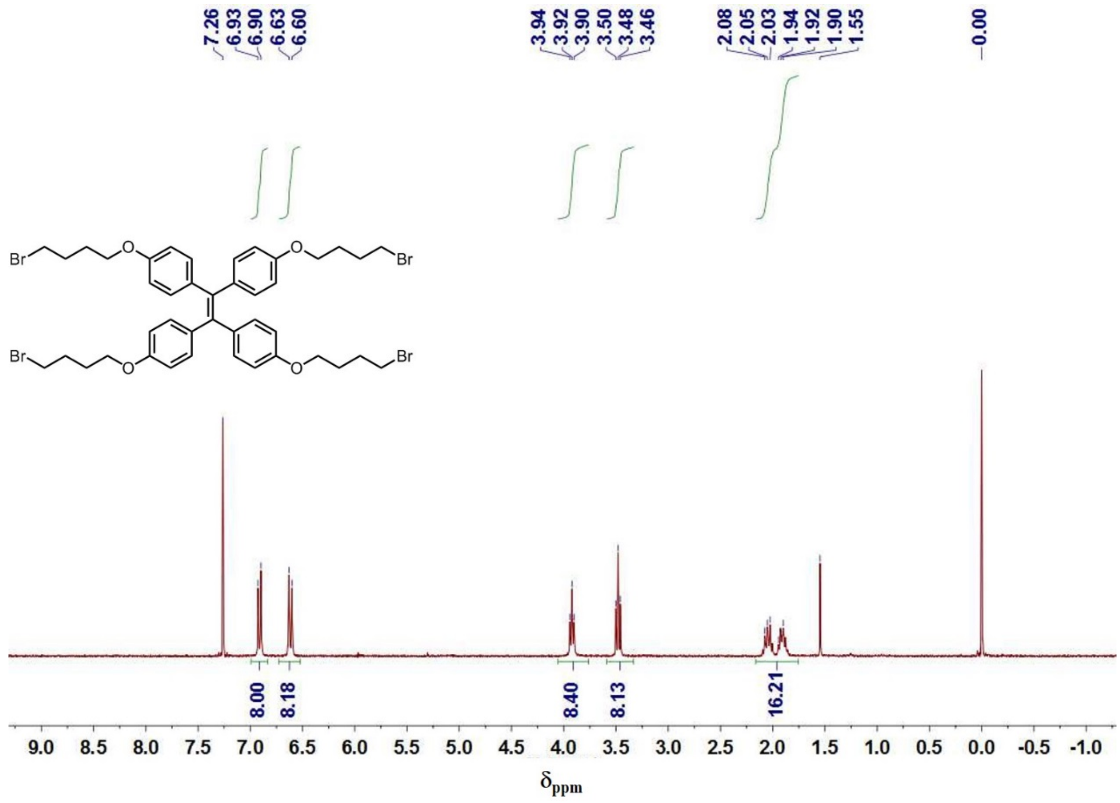

Figure S2. ^{1}H NMR spectrum (300 MHz, $CDCl_3$, 298 K) of TPE-$(Br)_4$.

3. Synthesis of MSP5

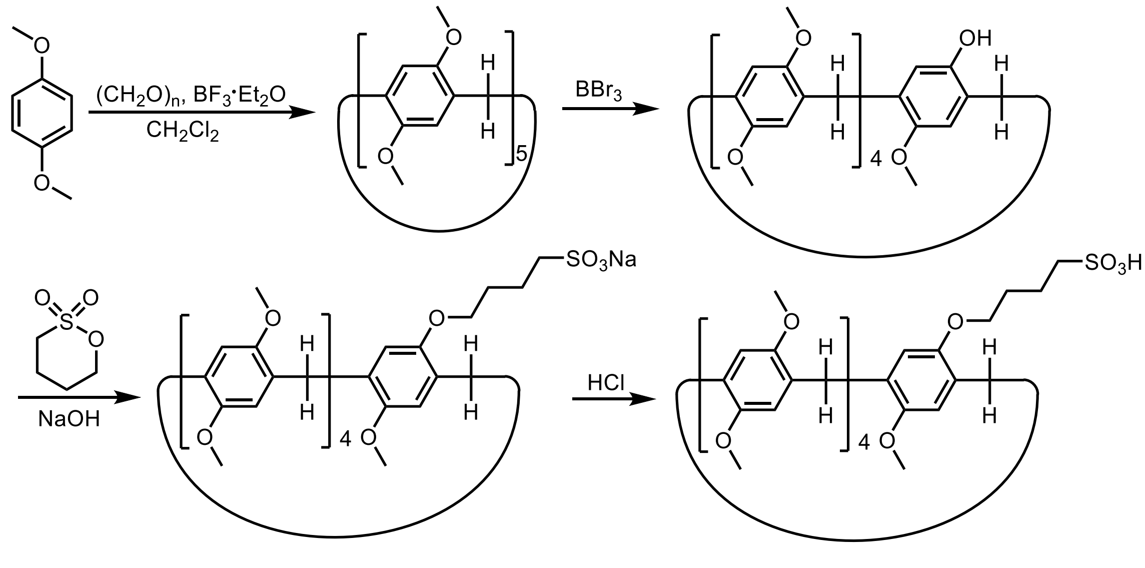

1,4-Dimethoxybenzene (7g, 50 mmol) and $(CH_2O)_n$ (4.5 g, 150 mmol) were dissolved

in dry DCM (500 mL) in a 1000 mL flask and stirred at 0 °C for 30 min. Then $BF_3 \cdot O(C_2H_5)_2$ (7 mL) was added into the flask under nitrogen atmosphere. Then the mixture was quenched with $NaHCO_3$ aqueous solution. After the solvent was removed, the obtain solid was purified by column chromatograph with petroleum ether/dichloromethane (1:2 v/v) to get the final product of DMP[5]. White powder: 3.2 g, 42.7 %. ^1H NMR (300 MHz, $CDCl_3$, 25 °C), δ (ppm): 6.77 (s, 10H), 3.77 (s, 10H), 3.65 (s, 30H).

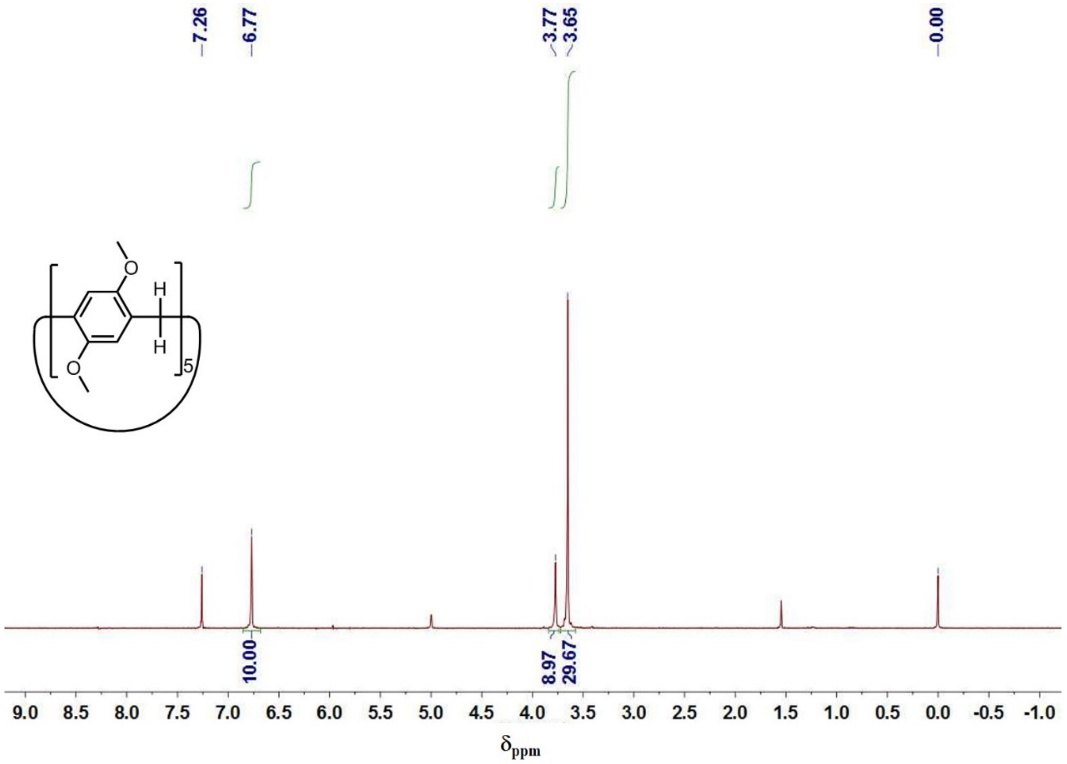

Figure S3. ^1H NMR spectrum (300 MHz, $CDCl_3$, 298 K) of DMP[5].

DMP[5] (3 g, 4 mmol) was dissolved in 100 mL $CHCl_3$ in a 250 mL flask. Then BBr_3 was added into the solution, the mixture was stirred at 0 °C for 1.5 h. Then the reaction was quenched with water and the organic phase was collected. After the solvent was removed, the obtain solid was purified by column chromatograph with petroleum ether/ethyl acetate (1:8 v/v) to get the final product of monohydroxyDMP[5]. White powder: 600 mg, 16 %. ^{1}H NMR (300 MHz, $CDCl_3$, 25 °C), δ (ppm): 6.70 (m, 11 H), 3.60 (m, 37 H).

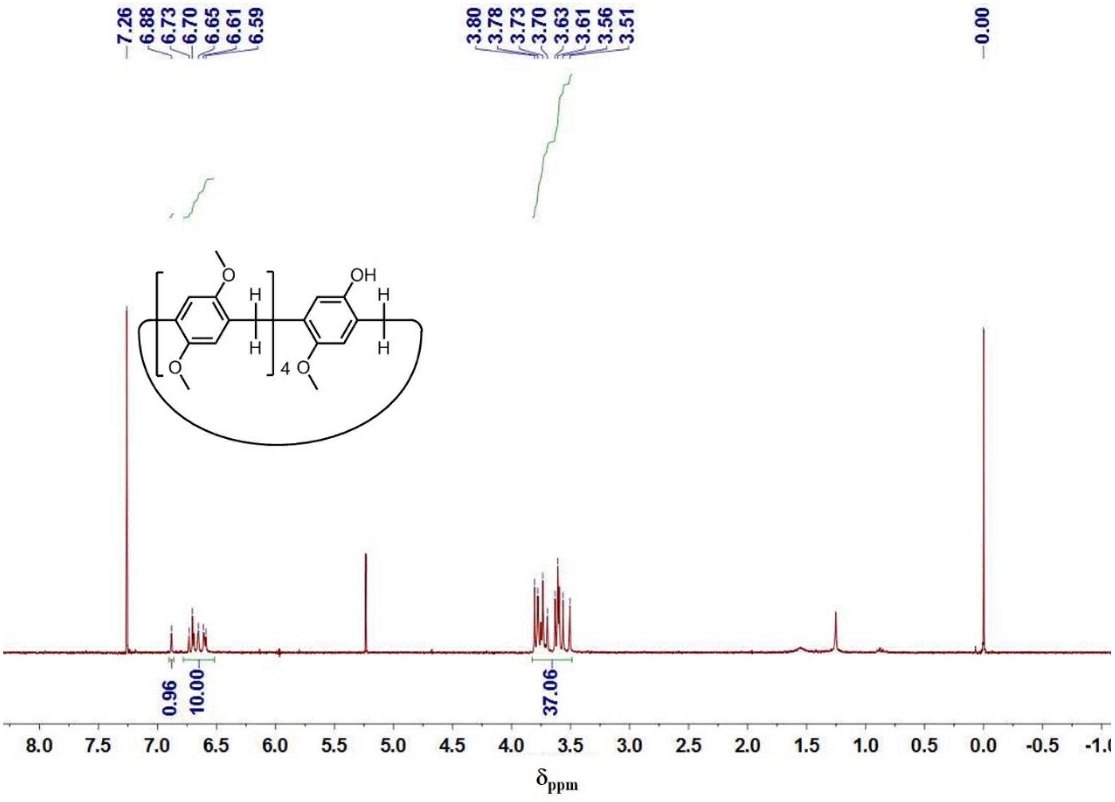

Figure S4. ^{1}H NMR spectrum (300 MHz, $CDCl_3$, 298 K) of monohydroxyDMP[5].

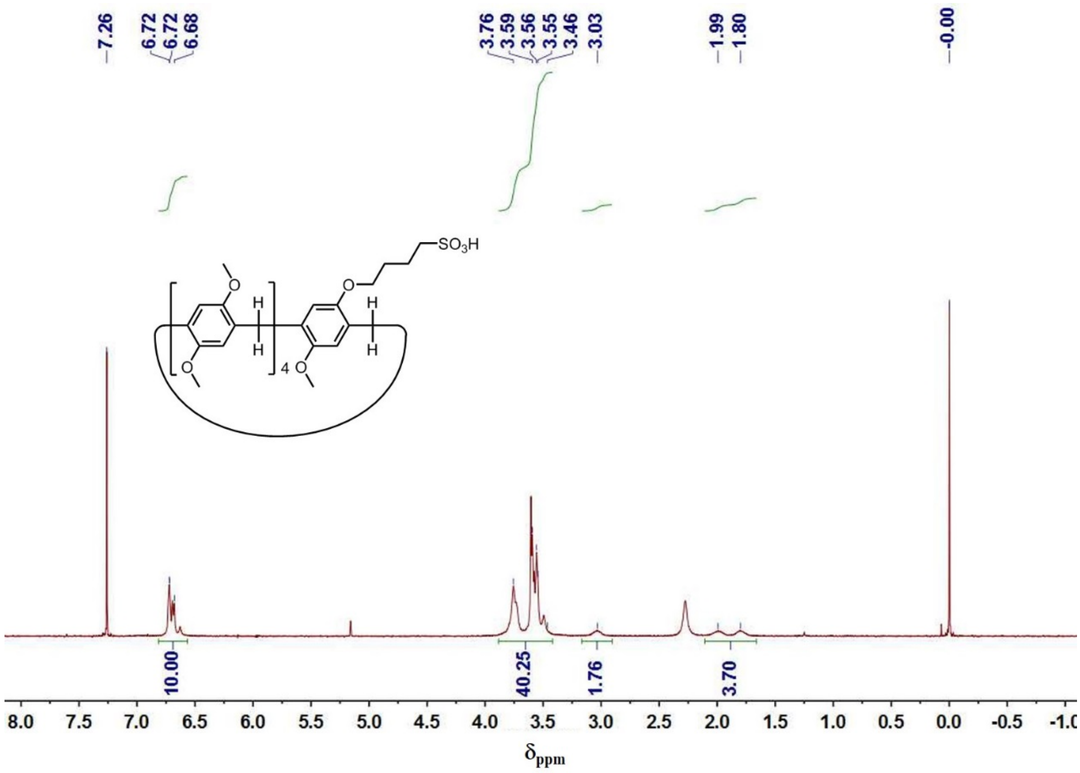

Figure S5. ^{1}H NMR spectrum (300 MHz, $CDCl_3$, 298 K) of MSP5.

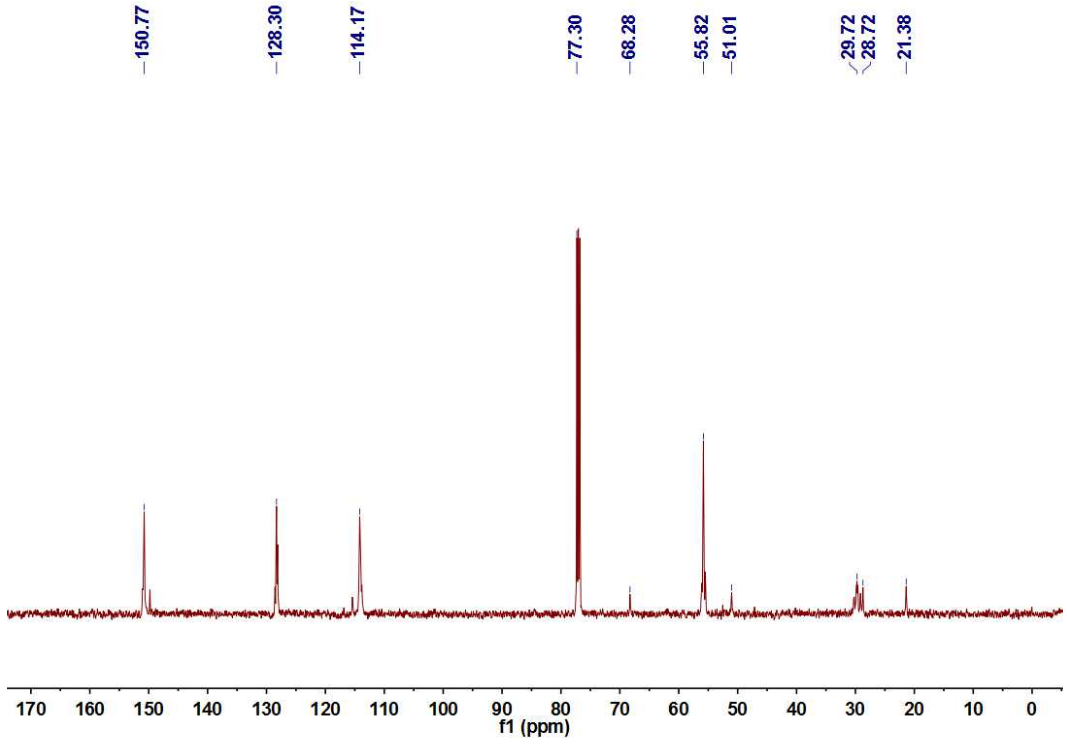

Figure S6. ^{13}C NMR spectrum (126 MHz, $CDCl_3$, 298 K) of MSP5.

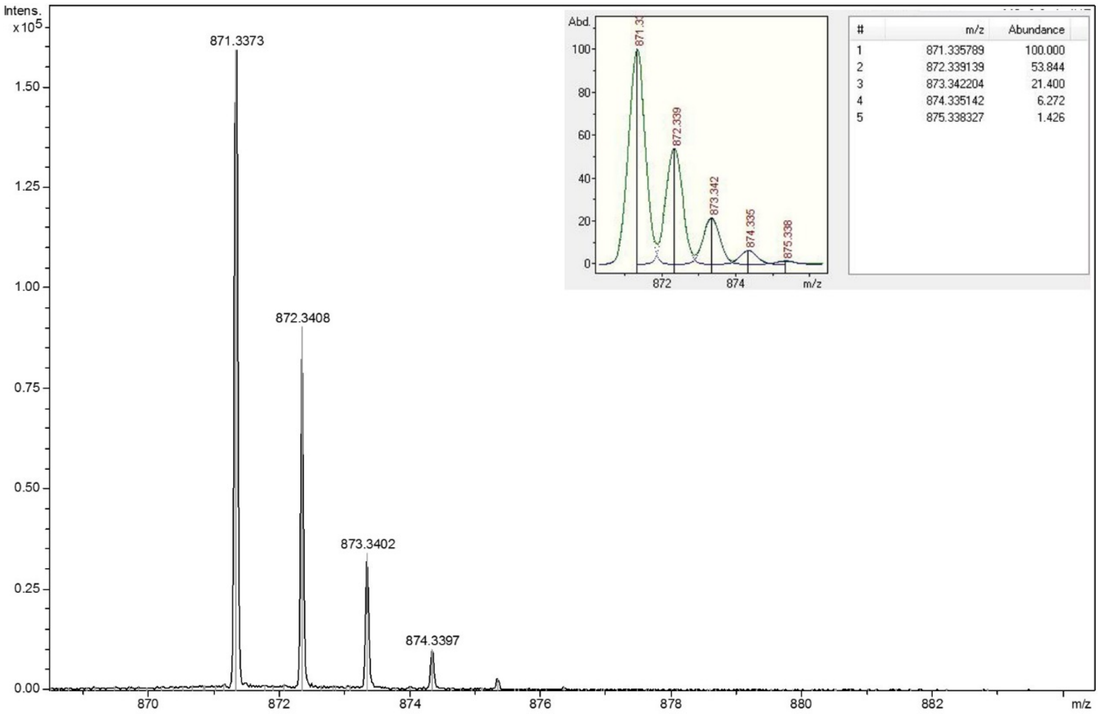

Figure S7. Electrospray ionization mass spectrum of MSP5. The peak at *m*/*z* 871.3373 corresponding to $[M - H]^-$ was clearly observed.

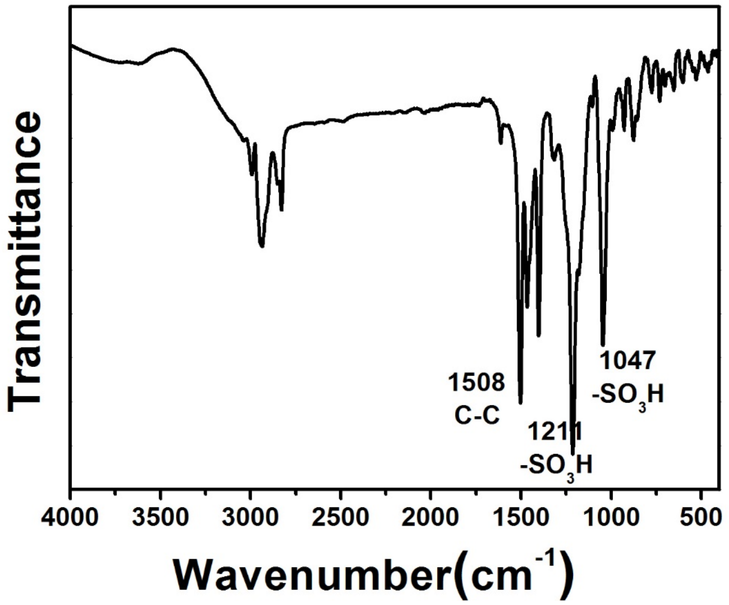

Figure S8. The FT-IR spectrum of MSP5. The peaks at 1211 cm^{-1} and 1047 cm^{-1} are the characteristic absorption of the sulfonic acid group vibration.

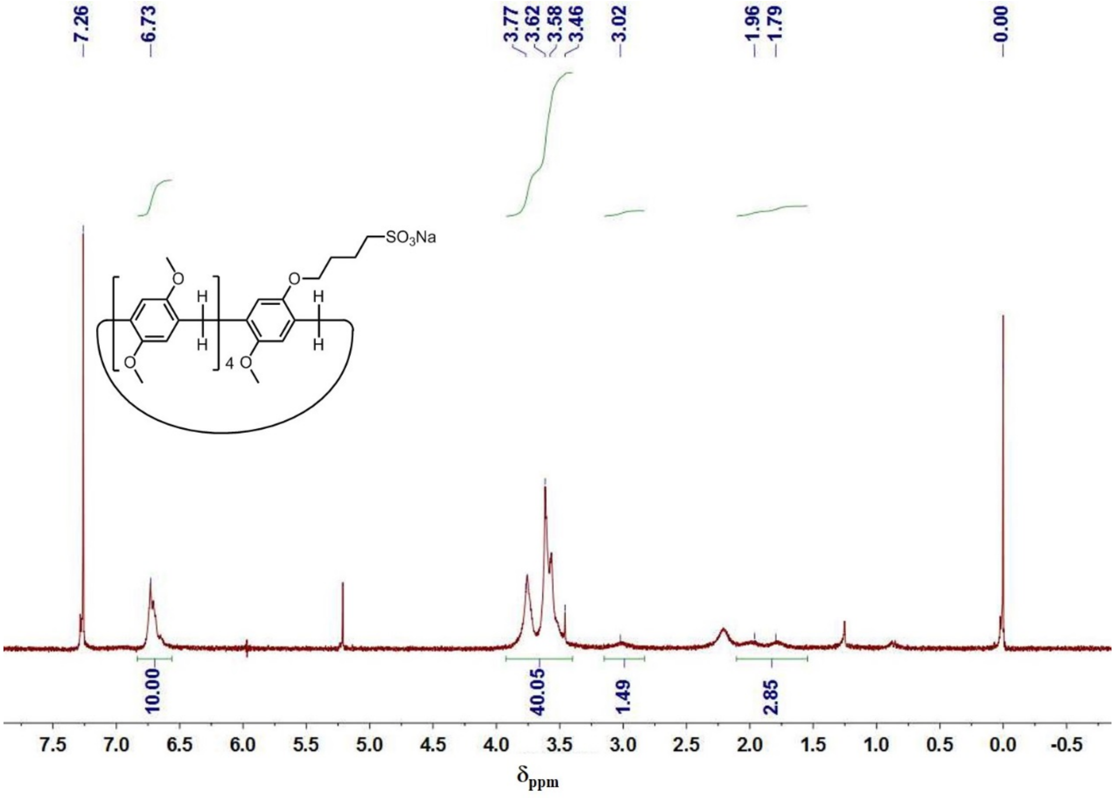

Figure S9. ^{1}H NMR spectrum (300 MHz, $CDCl_3$, 298 K) of monosulfonateDMP[5].

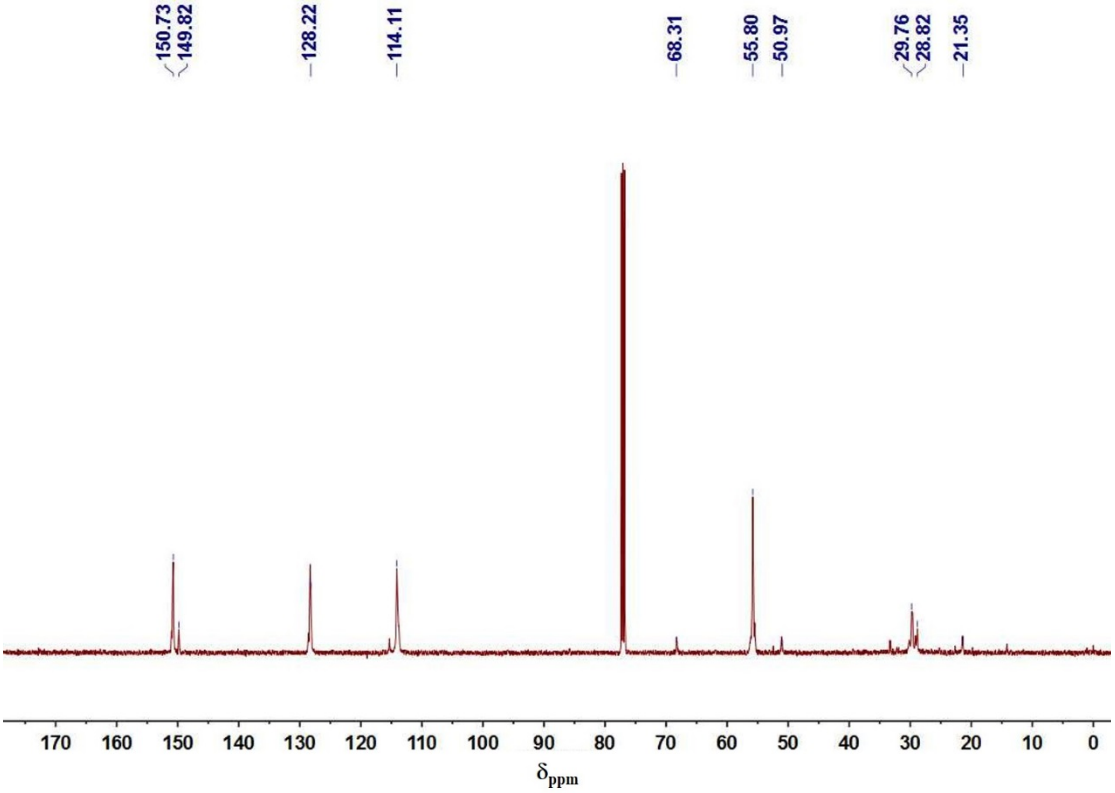

Figure S10. ^{13}C NMR spectrum (126 MHz, $CDCl_3$, 298 K) of monosulfonateDMP[5].

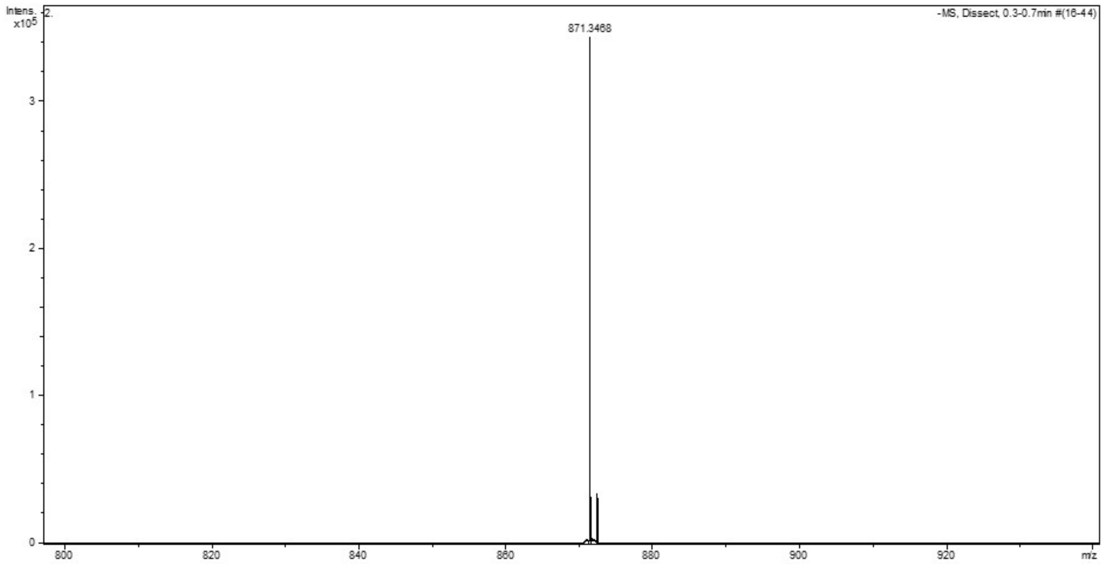

Figure S11. Electrospray ionization mass spectrum of monosulfonateDMP[5]. The peak at *m*/*z* 871.3468 corresponding to $[M - Na]^-$ was clearly observed.

4. Synthesis of M1[S2]

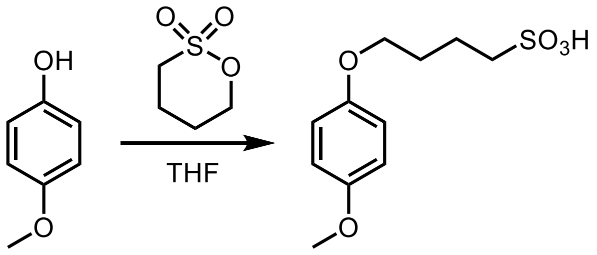

4-Methoxyphenol (1 g, 8 mmol) and NaOH (0.65 g, 16 mmol) were added into 100 mL THF. The mixture was stirred at room temperature for 30 min. Then 1,4-butylenesulfone (0.6 mL) was added into the above solution. The mixture was stirred under reflux for 24 h. Then the crude product was collected and washed with CH_2Cl_2. The obtained product was dissolved in 15 mL water, then hydrochloric acid was added into the aqueous solution dropwise. Then the mixture was stirred at room temperature for 12 h, concentrated and subjected to cation exchange resin to get pure product. White powder: 390 mg. ^1H NMR (300 MHz, D_2O, 25 °C), δ (ppm): 6.96 (m, 4 H), 4.04 (t, *J* = 6 Hz, 2 H), 3.78 (s, 3 H), 2.95 (t, *J* = 6 Hz, 2 H), 1.86 (m, 4 H).

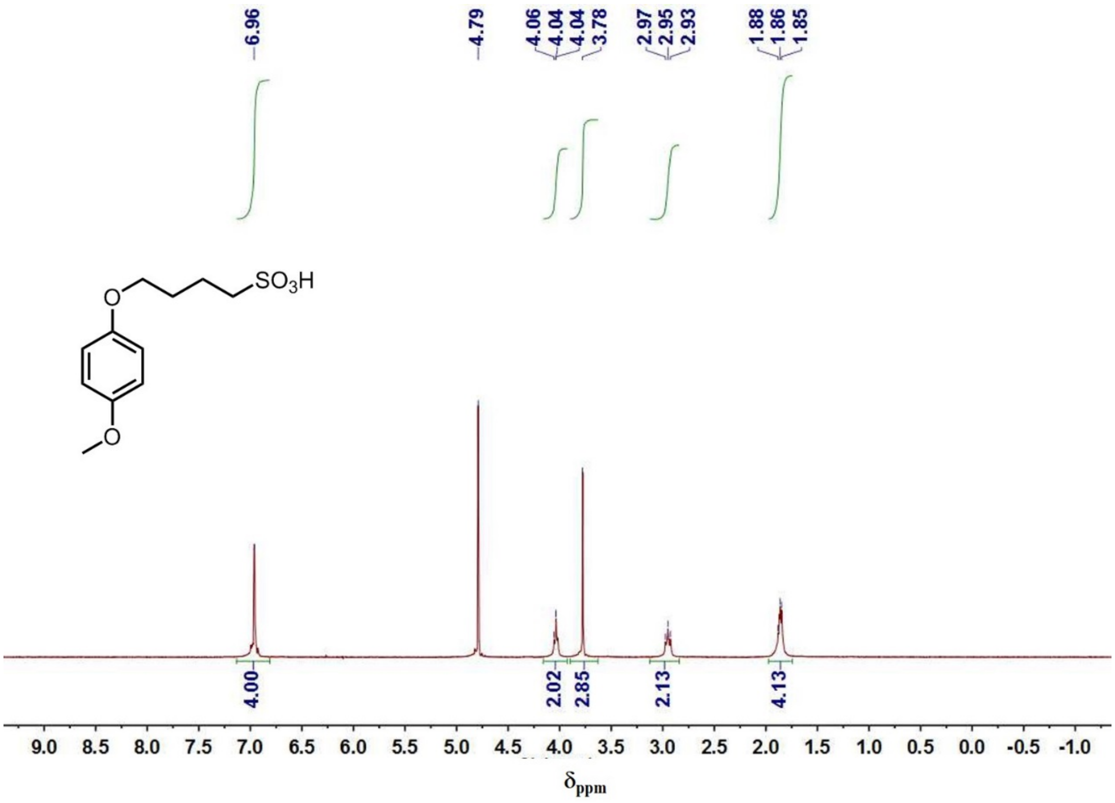

Figure S12. ^{1}H NMR spectrum (300 MHz, D_2O, 298 K) of M1.

5. Synthesis of G1[S3]

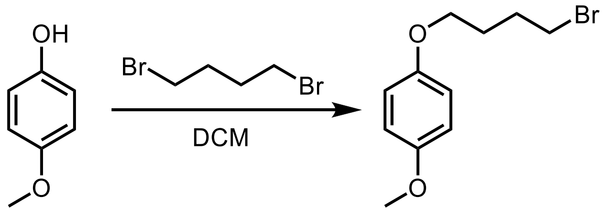

4-Methoxyphenol (12.4 g, 0.1 mol) and K_2CO_3 (22 g) were added into MeCN (400 mL). The mixture was stirred at room temperature for 30 min. Then excess 1,4-dibromobutane (15 mL, 0.125 mol) was added into the mixture. The mixture was stirred under reflux for 12 h, filtered and washed with dichloromethane. After the solvent was removed, the obtained solid was purified by column chromatograph with petroleum ether/ethyl acetate (15:1 v/v) to get the final product. White powder: 16.24 g, 63 %. ^{1}H NMR (300 MHz, $CDCl_3$, 25 °C), δ (ppm): 6.83 (s, 4 H), 3.95 (t, J = 6 Hz, 2 H), 3.76 (s, 3 H), 3.49 (t, J = 6 Hz, 2 H), 1.96 (m, 2H), 1.89 (m, 2H).

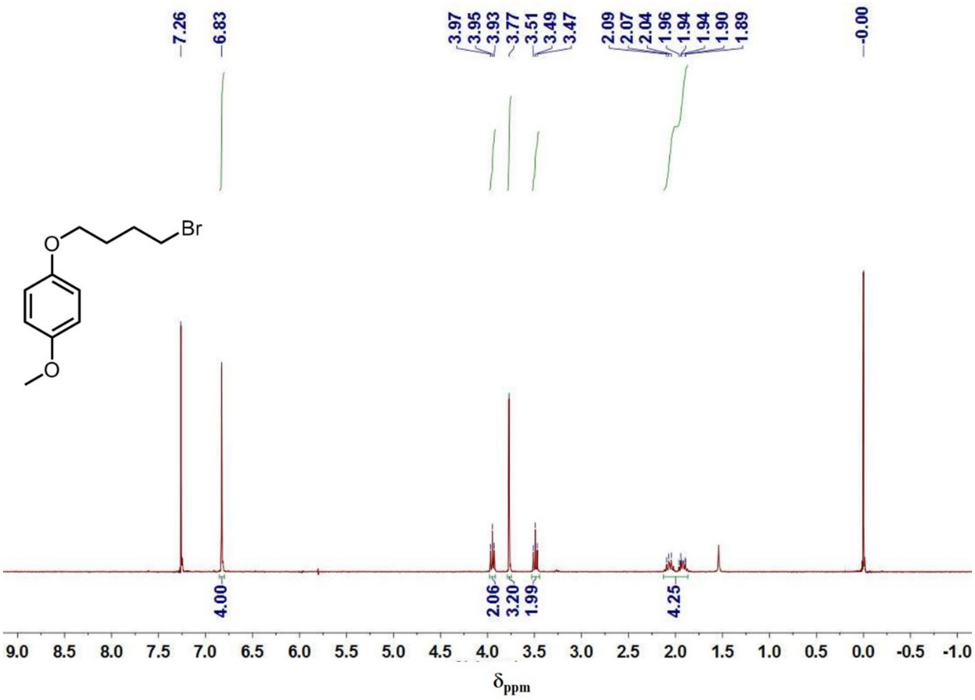

Figure S13. ^{1}H NMR spectrum (300 MHz, $CDCl_3$, 298 K) of G1.

6. Synthesis of TPE-(CN)$_4$

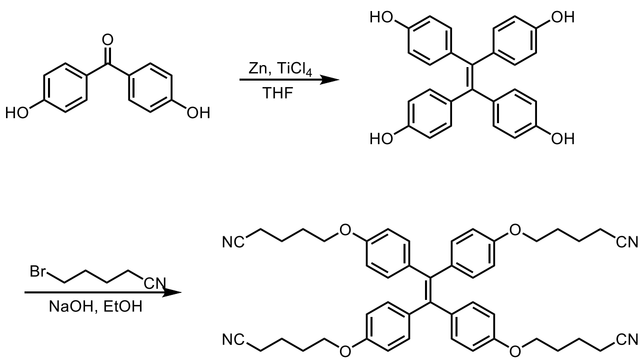

Znic (3.4 g) and titaniumtetrachloride (3.4 mL) was added into 80 mL dry tetrahydrofuran at 0 °C. The mixture reacted at 0 °C for 30 min. Then 4,4'-hydroxybenzophenone (2.14 g) tetrahydrofuran solution was added into the above solution, reacted under reflux for 18 h. Then the mixture was quenched with potassium carbonate solution. After solvent was removed, the obtain solid was purified by column chromatograph with petroleum ether/ethyl acetate (1:1 v/v) to get the final product (tetra-hydroxyl-TPE). Pale yellow powder: 320 mg, 16 %. ^{1}H NMR (300 MHz, DMSO-d_6, 25 °C), δ (ppm): 9.22 (s, 4 H), 6.68 (d, J = 9 Hz, 8 H), 6.46 (d, J = 9 Hz, 8 H).

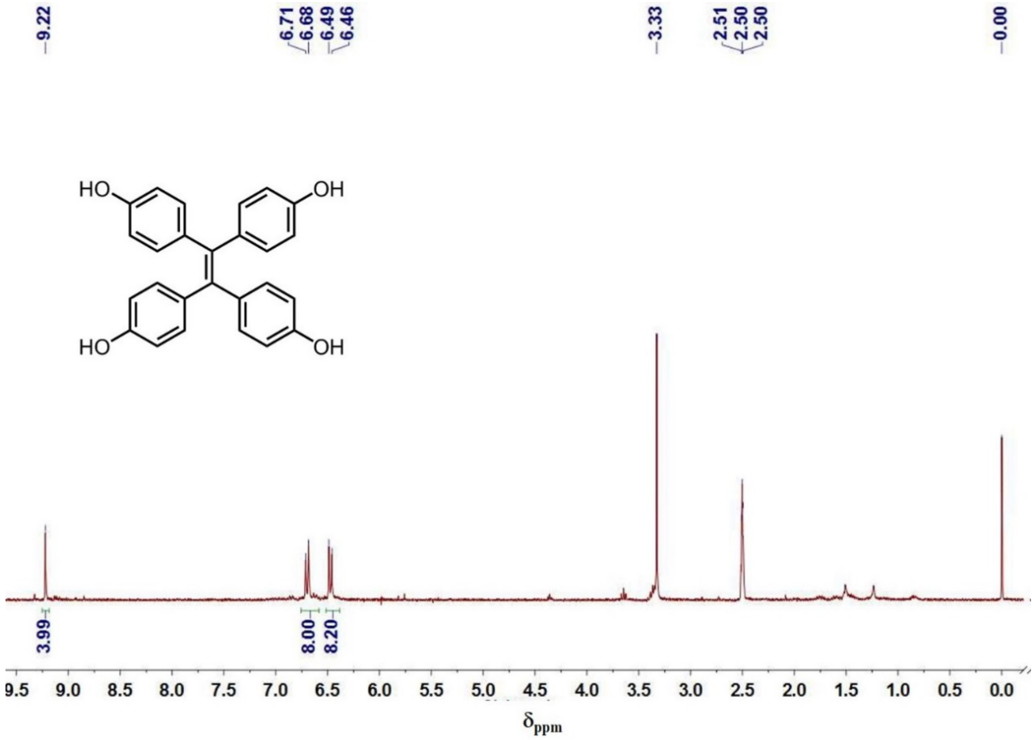

Figure S14. ^{1}H NMR spectrum (300 MHz, DMSO-d_6, 298 K) of tetra-hydroxyl-TPE.

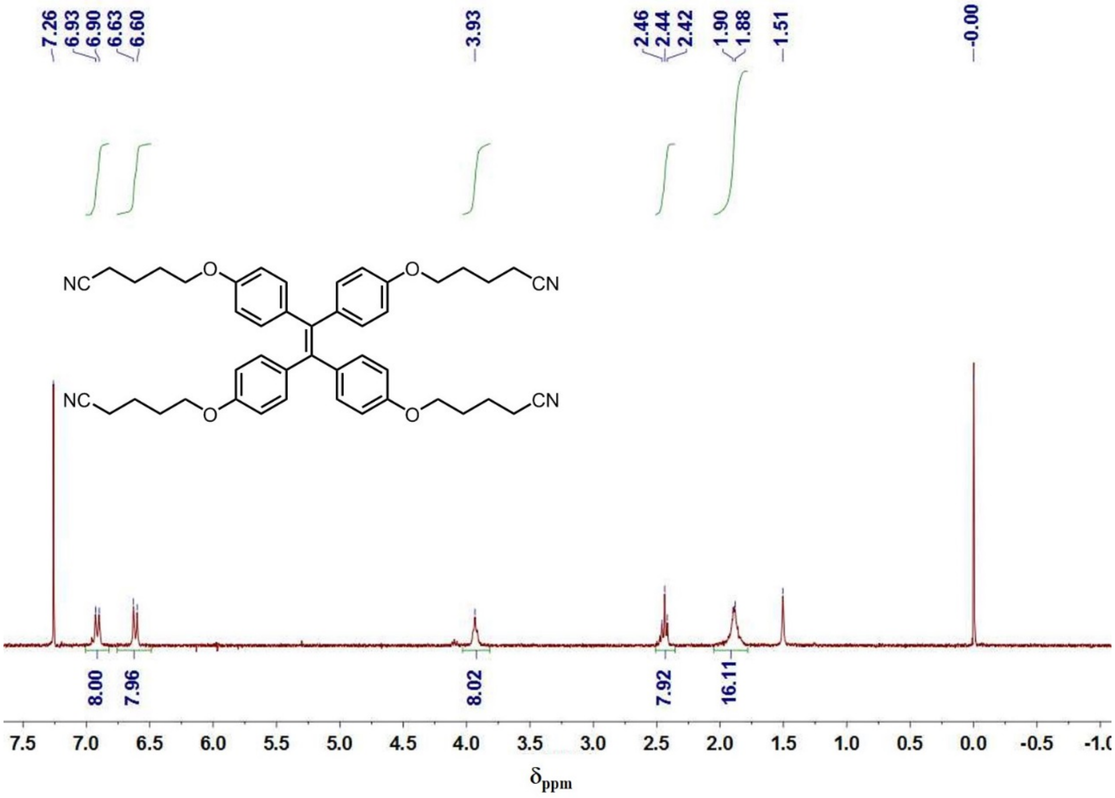

Figure S15. ^{1}H NMR spectrum (300 MHz, $CDCl_3$, 298 K) of TPE-$(CN)_4$.

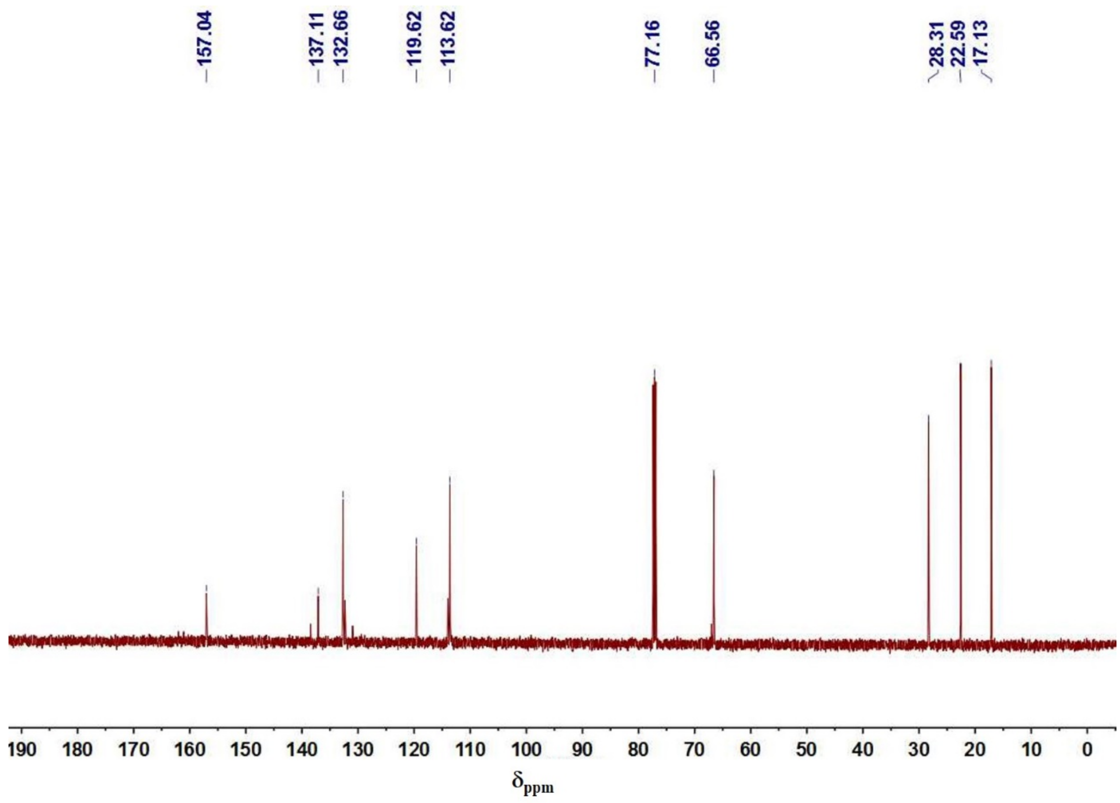

Figure S16. ^{13}C NMR spectrum (126 MHz, $CDCl_3$, 298 K) of TPE-$(CN)_4$.

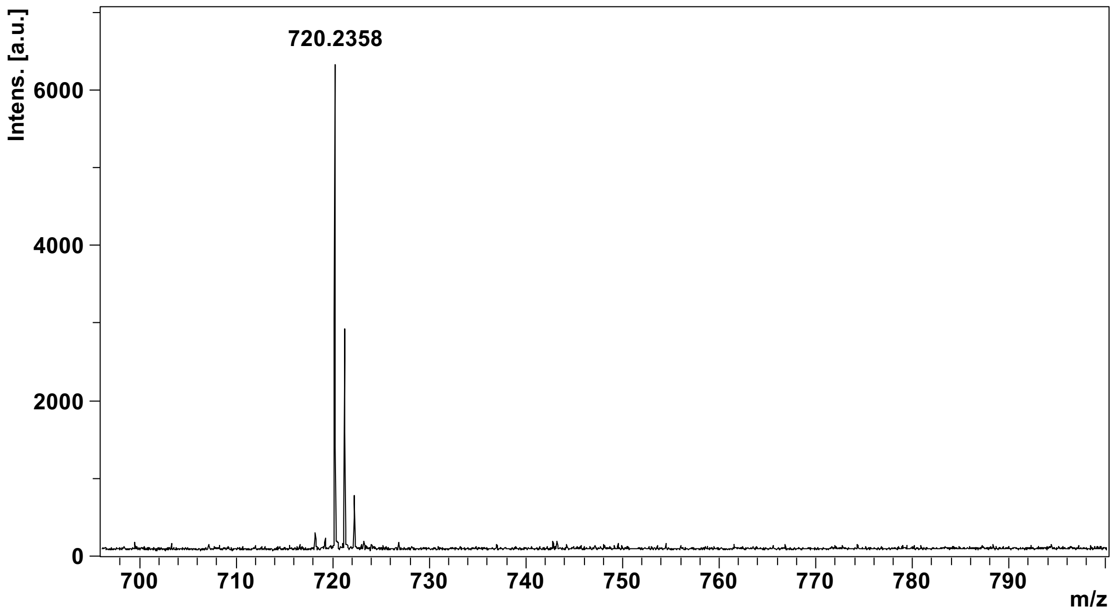

Figure S17. MOLDI-TOF mass spectrum of TPE-$(CN)_4$. The peak at *m*/*z* 720.2358 corresponding to [M] was clearly observed.

7. Synthesis of MCP5

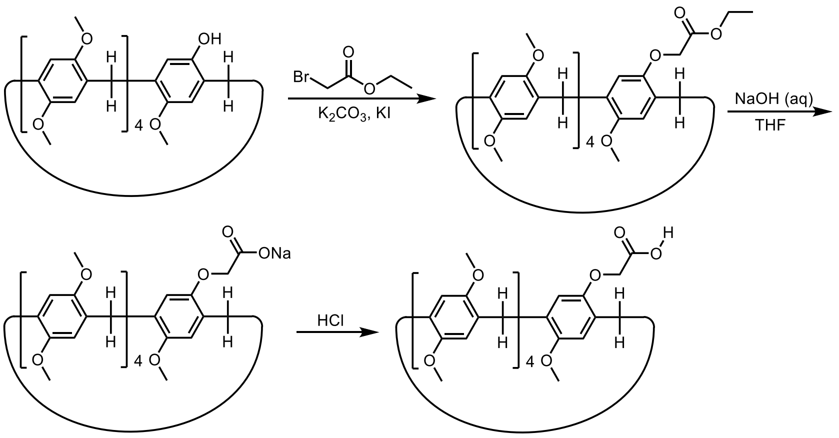

MonohydroxyDMP[5] (0.44 g, 0.6 mmol), ethyl bromoacetate (0.1 mL, 0.9 mmol), potassium carbonate (0.16 g, 1.2 mmol) and potassium iodide (20 mg, 0.12 mmol) were added into 50 mL acetonitrile. The mixture was stirred under reflux for 24 h. After solvent was removed, the obtain solid was recrystallized with dichloromethane / n-hexane to give monoesterpillar[5]arene, which was further used for the synthesis of monocarboxylatepillar[5]arene sodium salt (see the main text). White powder: 300 mg, 61%. ^{1}H NMR (300 Hz, CDCl$_3$, 25 °C), δ (ppm): 6.58~6.91 (m, 10 H), 4.51(s,2 H), 3.63~3.80 (m, 37H), 2.18 (m, 2 H), -1.47 (t, J = 6 Hz, 2 H).

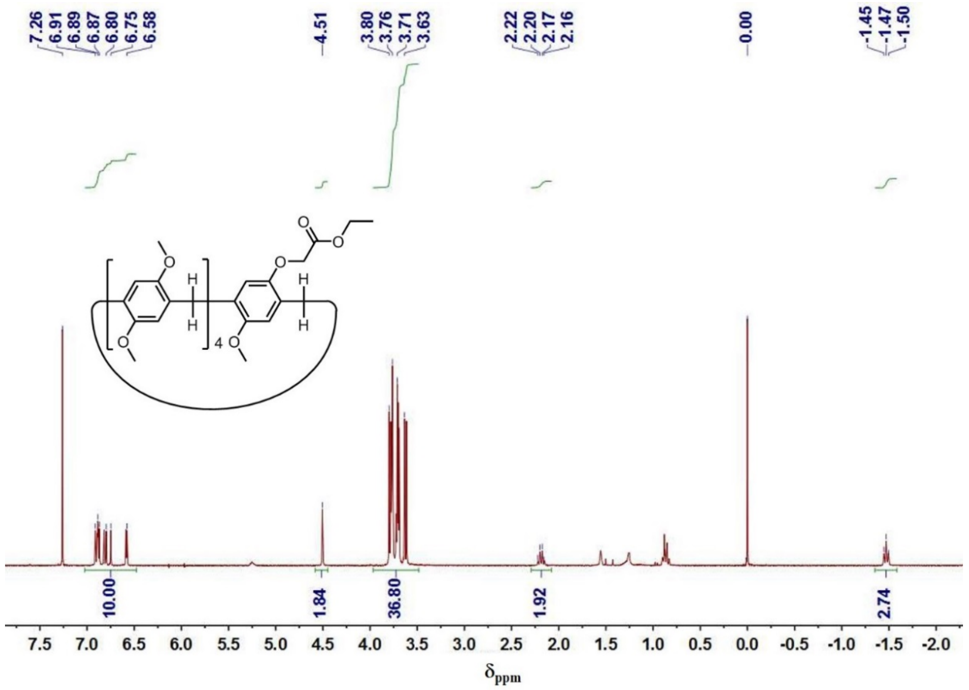

Figure S18. ^{1}H NMR spectrum (300 MHz, CDCl$_3$, 298 K) of monoesterpillar[5]arene.

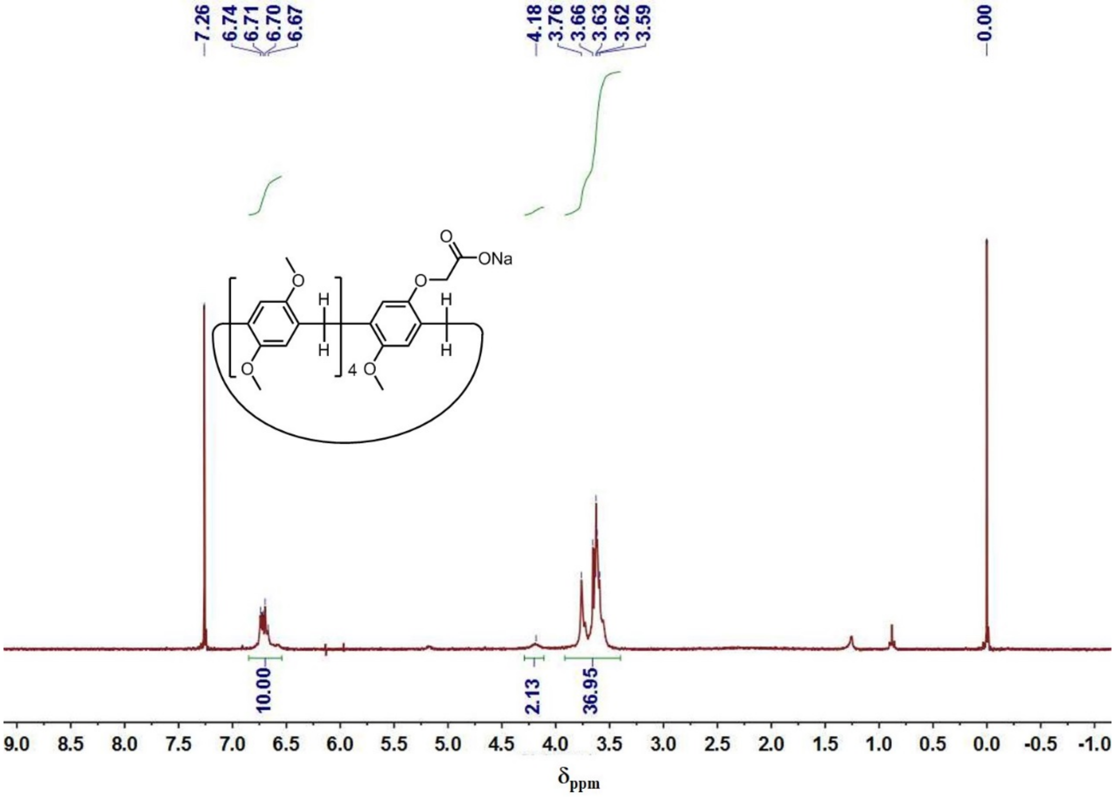

Figure S19. ^{1}H NMR spectrum (300 MHz, $CDCl_3$, 298 K) of monocarboxylatepillar[5]arene.

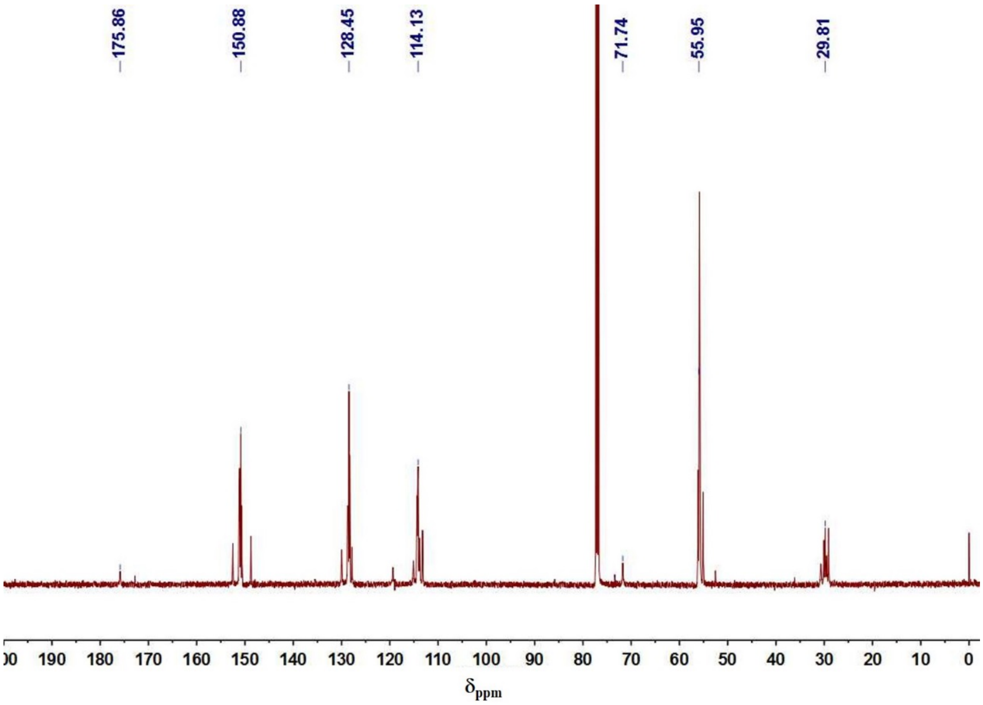

Figure S20. ^{13}C NMR spectrum (126 MHz, $CDCl_3$, 298 K) of monocarboxylatepillar[5]arene.

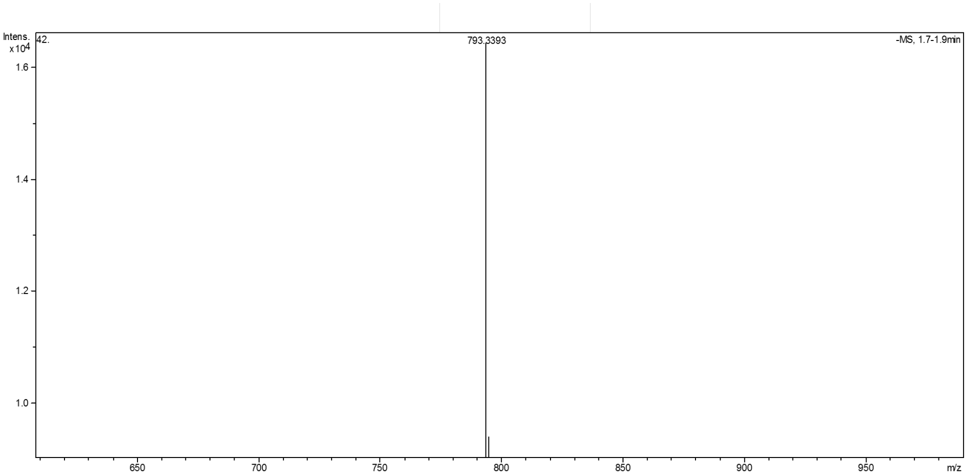

Figure S21. Electrospray ionization mass spectrum of MCP5Na. The peak at *m*/*z* 793.3393 corresponding to $[M - Na]^-$ was clearly observed.

After putting monocarboxylatepillar[5]arene sodium salt (280 mg) into a mixed solvent of water/THF (20 mL, 1:1), hydrochloric acid was added to neutralize it. The crude product was separated, extracted and concentrated. The obtained product was recrystallized to get the final product of MCP5. White powder: 260 mg, 93 %. ^{1}H NMR (300 MHz, $CDCl_3$, 25 °C), δ (ppm): 6.55~6.82 (m, 10 H), 4.30 (s,2 H), 3.53~3.82 (m, 37 H).

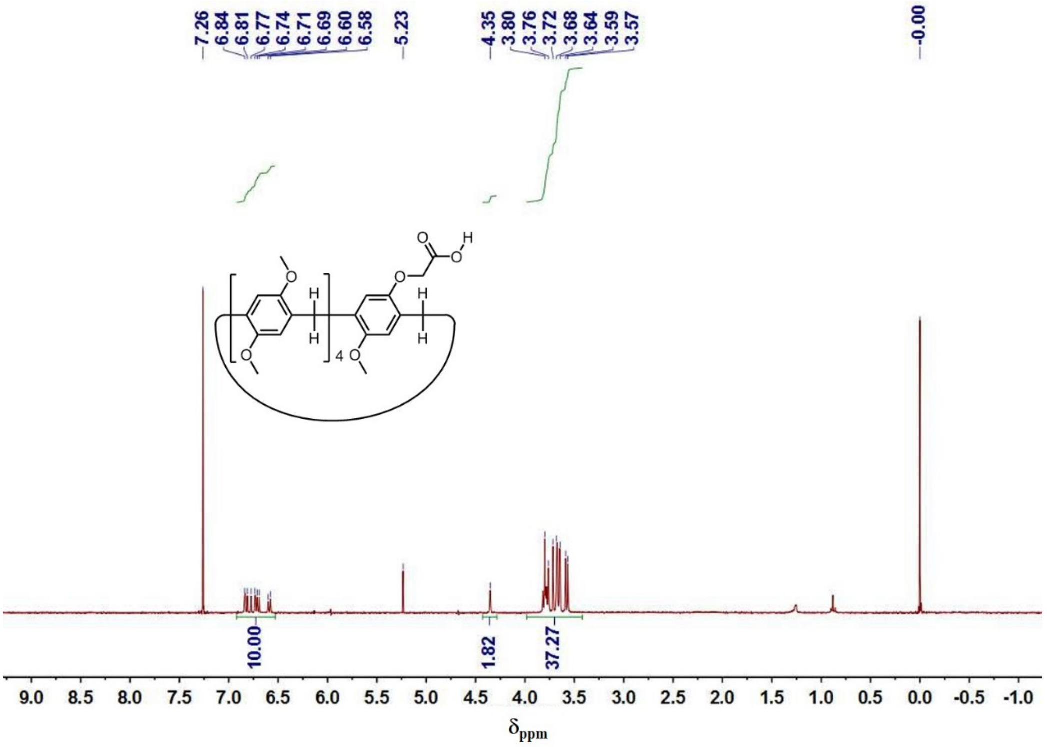

Figure S22. ^{1}H NMR spectrum (300 MHz, $CDCl_3$, 298 K) of MCP5.

8. Synthesis of Monophosphoricpillar[5]arene

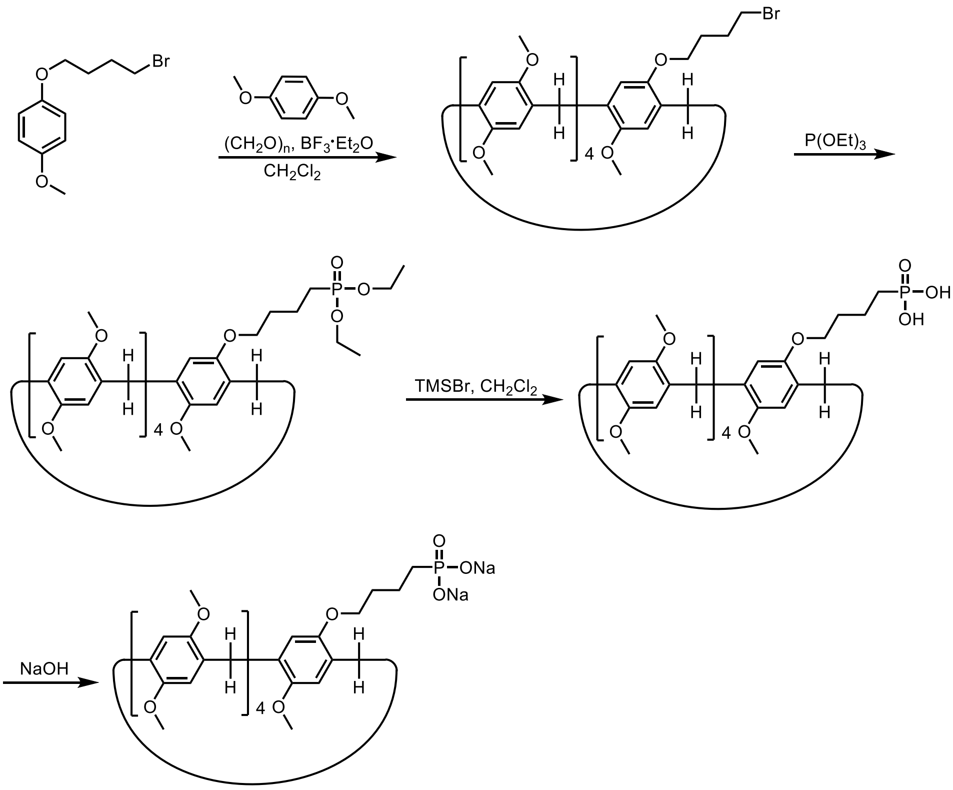

1,4-Dimethoxybenzene (2.75 g, 20 mmol), 4-bromobutyl-anisole (1.04 g, 4 mmol) and paraformaldehyde (2.2 g) were added into dried dichloromethane (180 mL). And then boron fluoride ethyl ether (3.6 mL) was added into the mixture at 0 °C and reacted for 30 min. Then the mixture was added with water to quench the reaction. After the solvent was removed, the obtained solid was purified by column chromatograph with petroleum ether/dichloromethane/ethyl acetate (90:30:1 v/v). White powder: 610 mg, 17.5 %. ^{1}H NMR (300 MHz, $CDCl_3$, 25 °C), δ (ppm): 6.70~6.80 (m, 10 H), 3.78 (m, 12 H), 3.65 (m, 27 H), 3.21 (s, 2 H), 1.77 (s, 4 H).

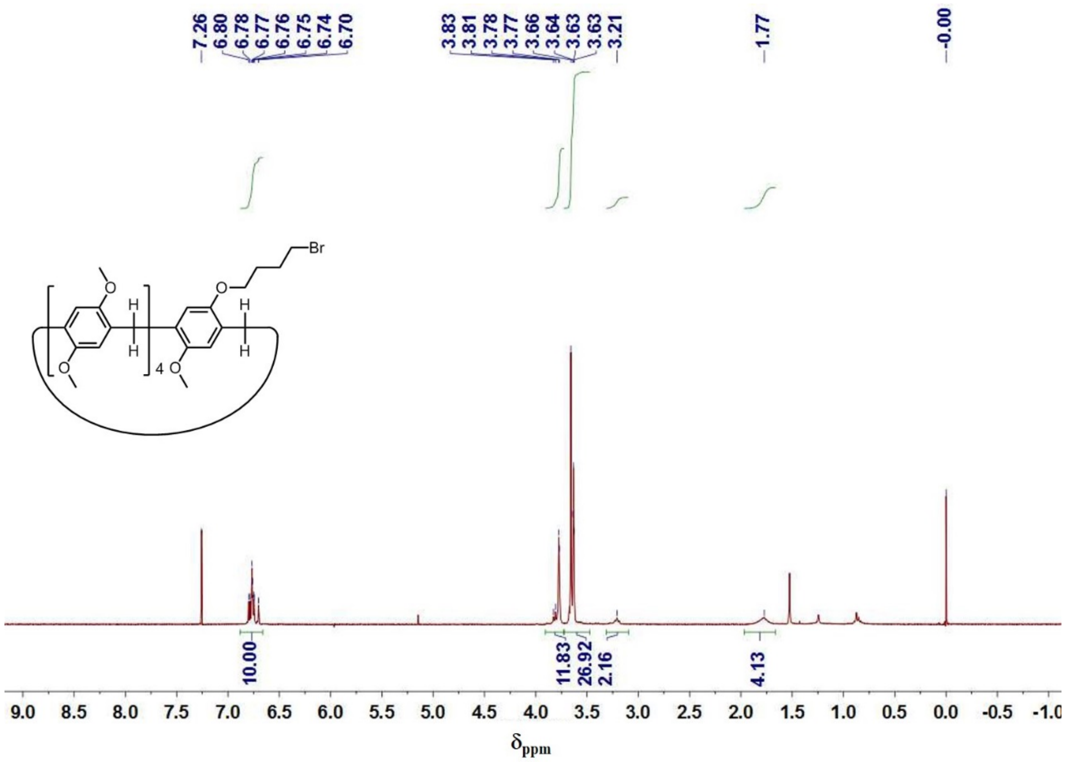

Figure S23. ^{1}H NMR spectrum (300 MHz, $CDCl_3$, 298 K) of monobromocopillar[5]arene.

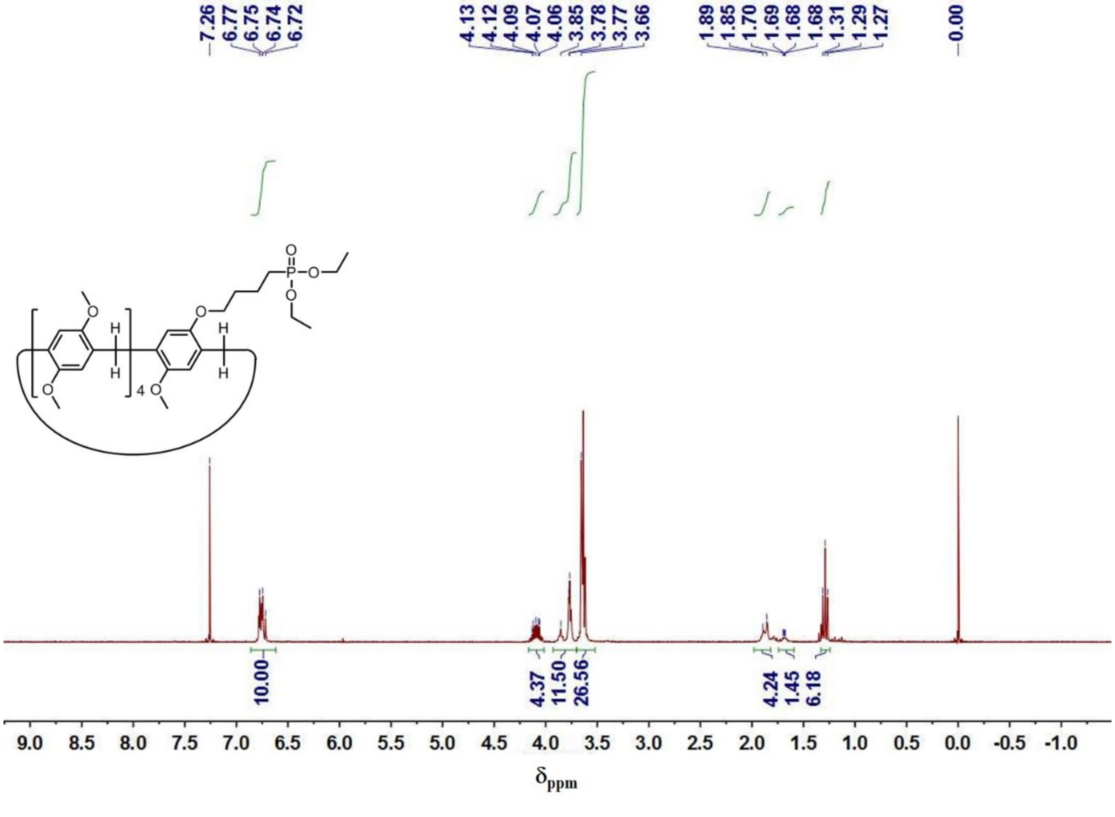

Figure S24. ^{1}H NMR spectrum (300 MHz, $CDCl_3$, 298 K) of monophosphite-DMP[5].

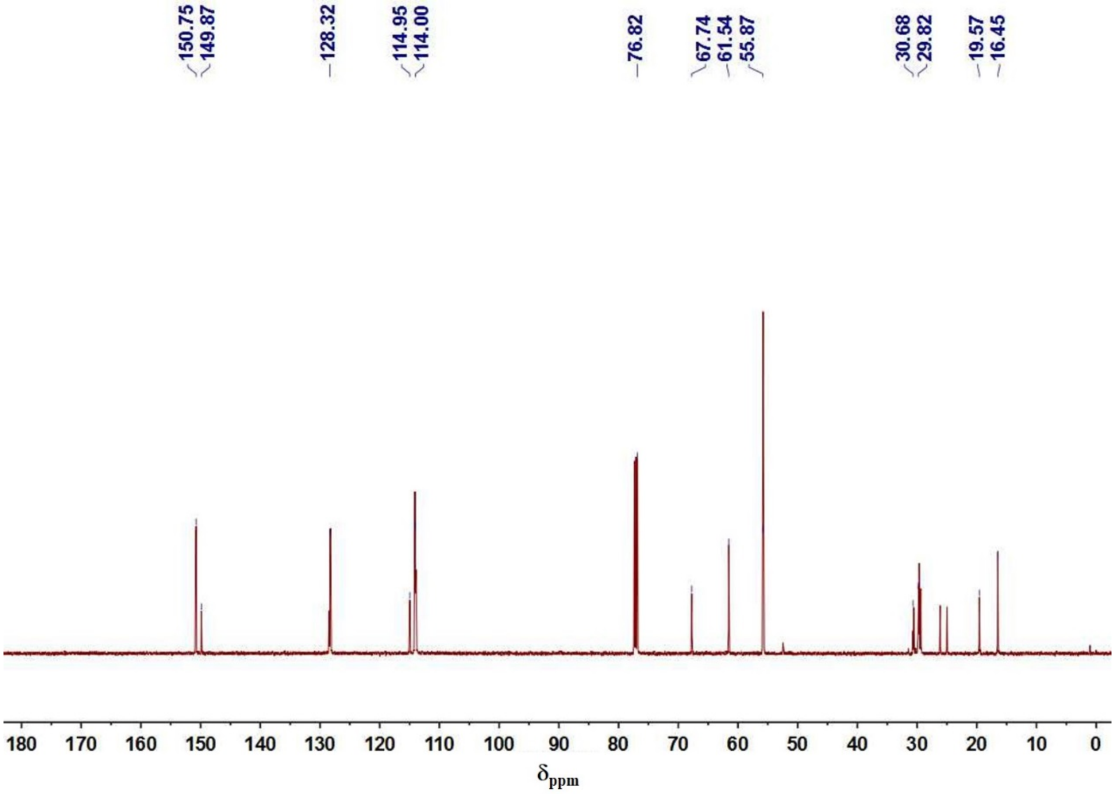

Figure S25. ^{13}C NMR spectrum (126 MHz, $CDCl_3$, 298 K) of monophosphiteDMP[5].

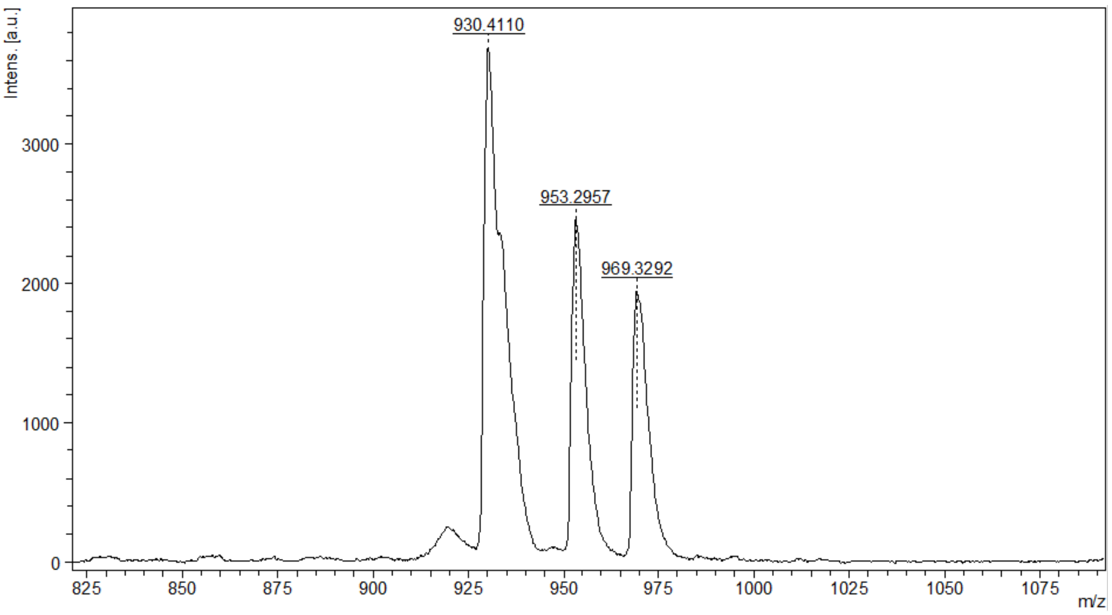

Figure S26. MOLDI-TOF mass spectrum of monophosphiteDMP[5]. The peak at *m*/*z* 930.4110 corresponding to $[M+H]^{+}$, 953.2957 corresponding to $[M+H+Na]^{2+}$, 969.3292 corresponding to $[M+H+K]^{2+}$ was clearly observed.

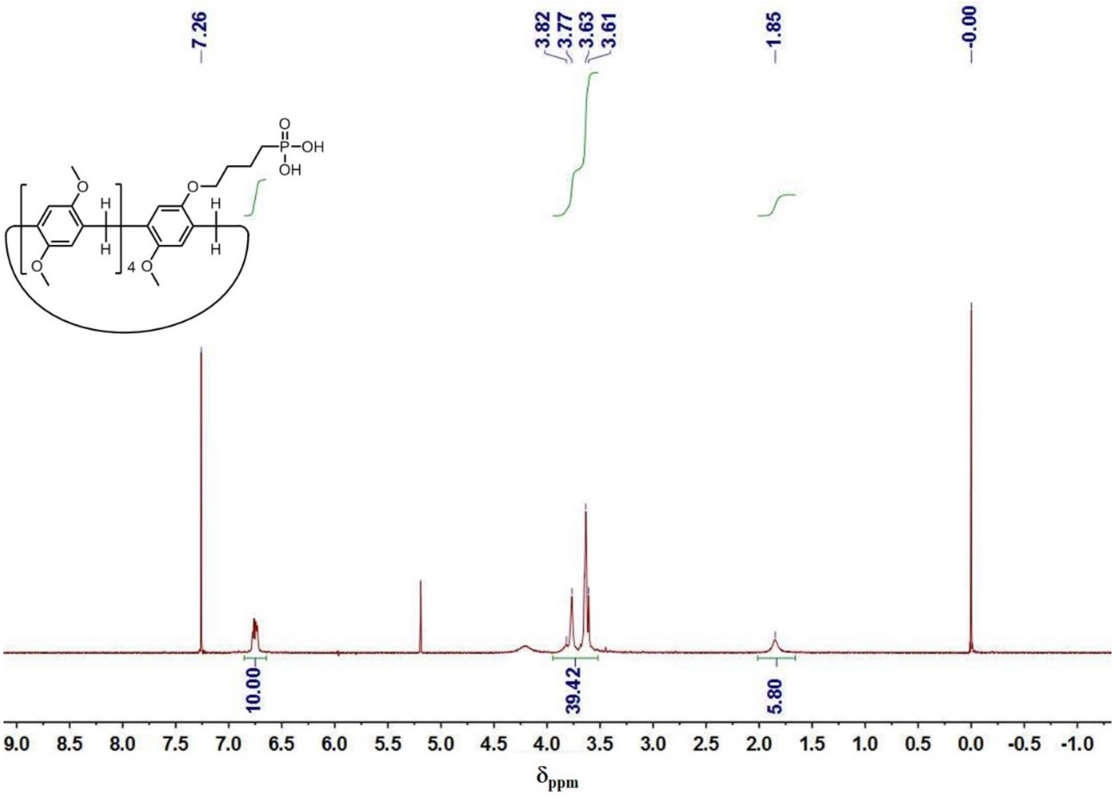

Figure S27. ^{1}H NMR spectrum (300 MHz, $CDCl_3$, 298 K) of monophosphoricpillar[5]arene.

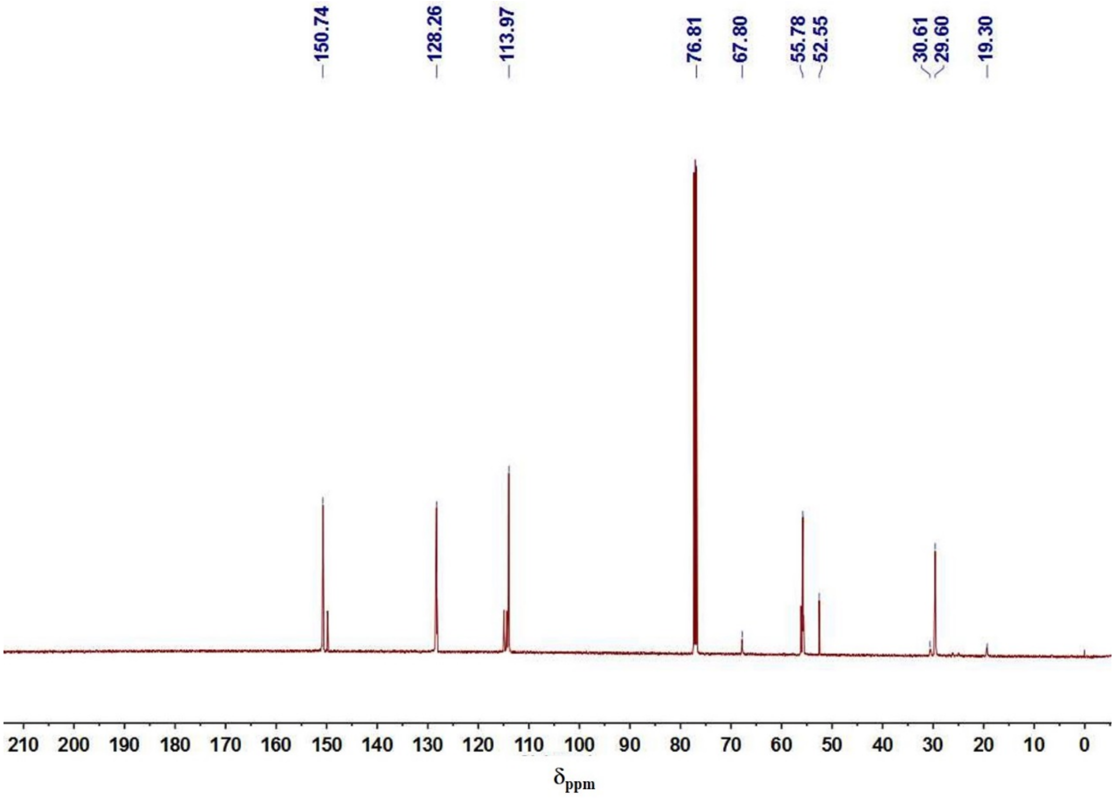

Figure S28. ^{13}C NMR spectrum (126 MHz, $CDCl_3$, 298 K) of monophosphoricpillar[5]arene.

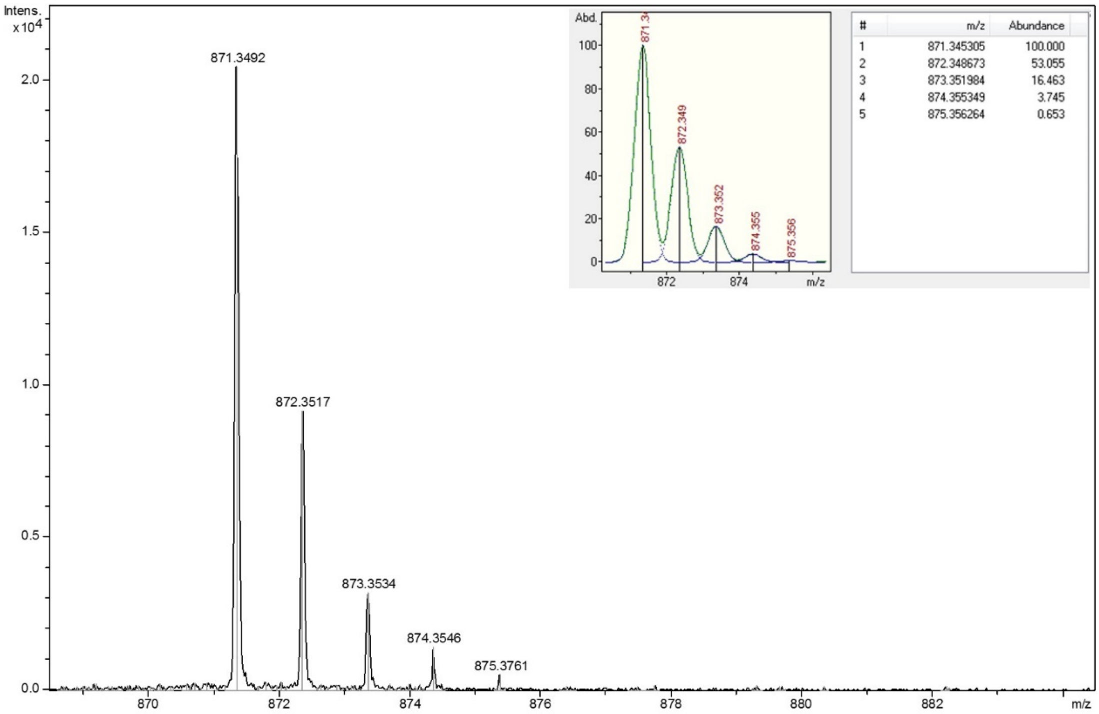

Figure S29. Electrospray ionization mass spectrum of monophosphoricpillar[5]arene. The peak at *m*/*z* 871.3453 corresponding to $[M - H]^-$ was clearly observed.

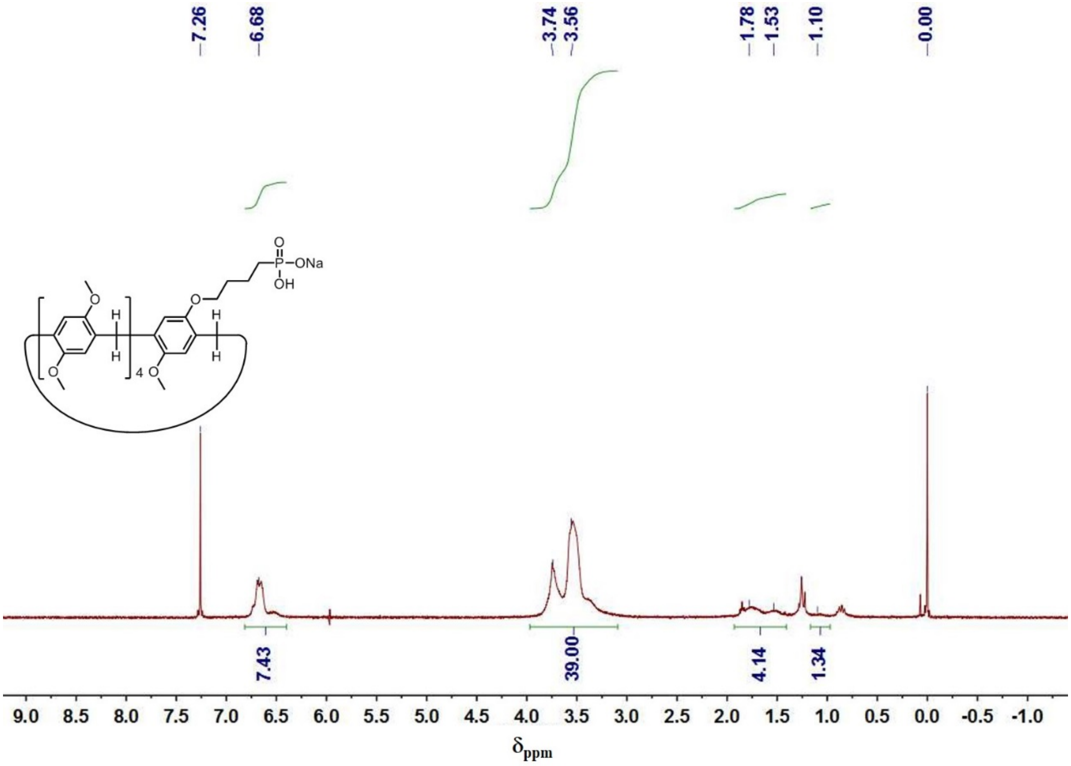

Figure S30. ^{1}H NMR spectrum (300 MHz, $CDCl_3$, 298 K) of monophosphatepillar[5]arene.

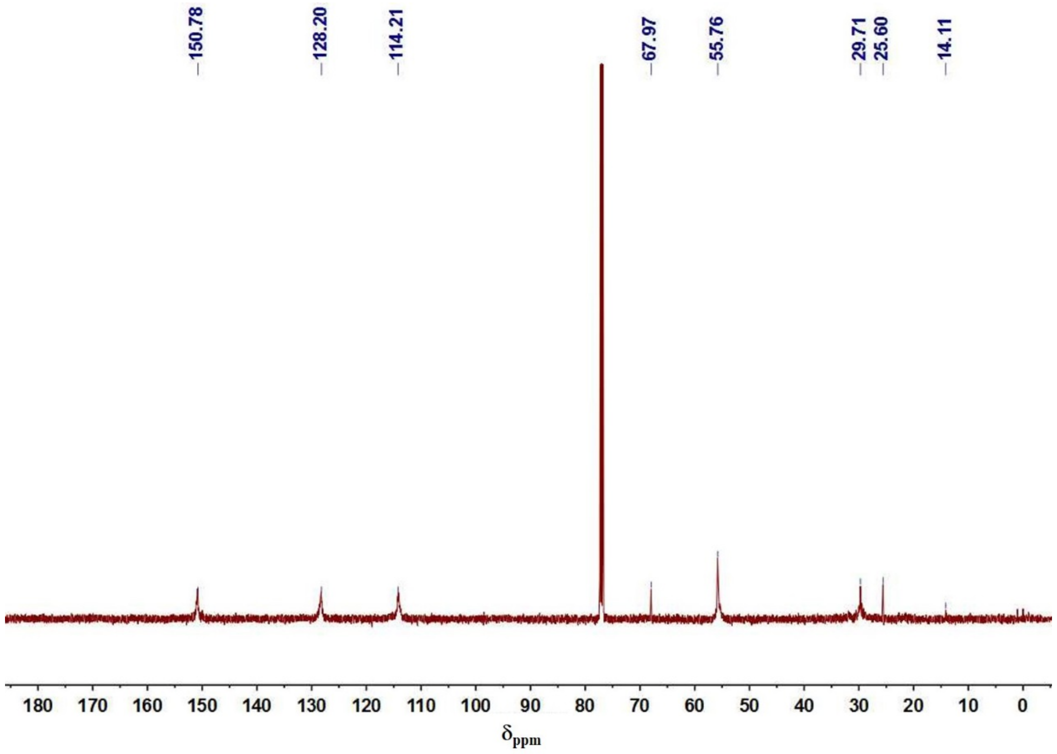

Figure S31. ^{13}C NMR spectrum (126 MHz, $CDCl_3$, 298 K) of monophosphatepillar[5]arene.

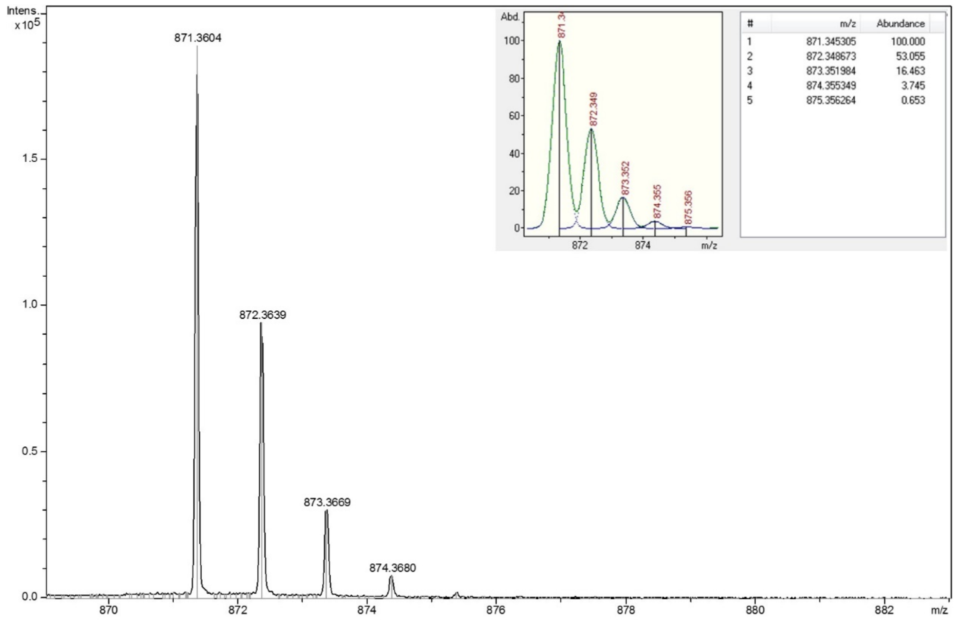

Figure S32. Electrospray ionization mass spectrum of monophosphatepillar[5]arene. The peak at *m*/*z* 871.3604 corresponding to $[M - Na]^-$ was clearly observed.

9. Stoichiometry & association constant determination for alcohols⊂MSP5 in $CHCl_3$

To determine the stoichiometry and association constants of alcohols⊂MSP5, ^{1}H NMR titration was performed. By a nonlinear curve-fitting method, the association constants between the guests and host were calculated. Through a molar ratio plot, the stoichiometry was determined. The results are shown as follows:

Host	Guest	*Ka* (M^{-1})	Stoichiometry
MSP5	n-Butanol (BO)	$(0.99 \pm 0.17) \times 10^2$	1:1
	n-Pentanol (PO)	$(1.58 \pm 0.35) \times 10^2$	1:1
	n-Hexanol (HO)	$(0.88 \pm 0.34) \times 10^2$	1:1
	Butanediol (BDO)	$(3.79 \pm 0.83) \times 10^3$	1:1
	Pentanediol (PDO)	$(2.19 \pm 0.21) \times 10^2$	1:1
	Hexanediol (HDO)	$(2.20 \pm 0.07) \times 10^2$	1:1
MPP5	n-Butanol (BO)	$(3.45 \pm 0.61) \times 10^2$	1:1
	Butanediol (BDO)	$(1.48 \pm 0.21) \times 10^3$	1:1
DMP5	n-Butanol (BO)	-	
	Butanediol (BDO)	$(2.91 \pm 0.44) \times 10^2$	1:1

Figure S33. Summary of the association constant (Ka/M^{-1}) and stoichiometry of the complexations of host (MSP5, MPP5 and DMP5) and different guests in $CHCl_3$ at 298 K. (The data of MPP5 and DMP5 had been reported[S5])

(1) n-Butanol ⊂ MSP5

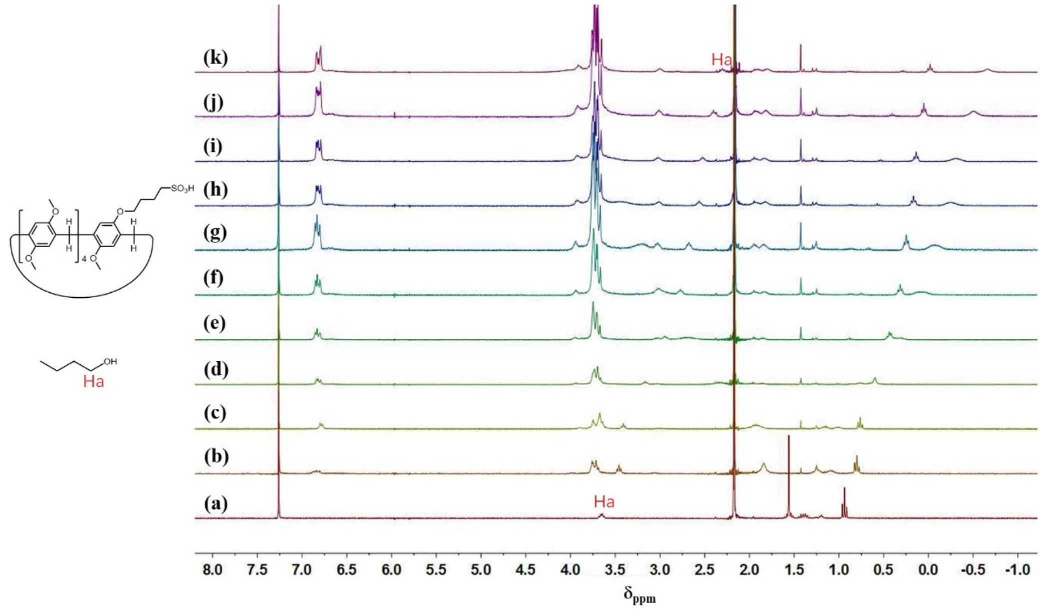

Figure S34. ^{1}H NMR titration (300 MHz, $CDCl_3$, 298 K) of n-butanol at a concentration of 5.00 mM with different concentrations (mM) of MSP5 in $CDCl_3$. (a) 0.00, (b) 1.00, (c) 2.00, (d) 3.00, (e) 4.00, (f) 5.00, (g) 6.00, (h) 7.00, (i) 8.00, (j) 9.00, and (k) 10.00.

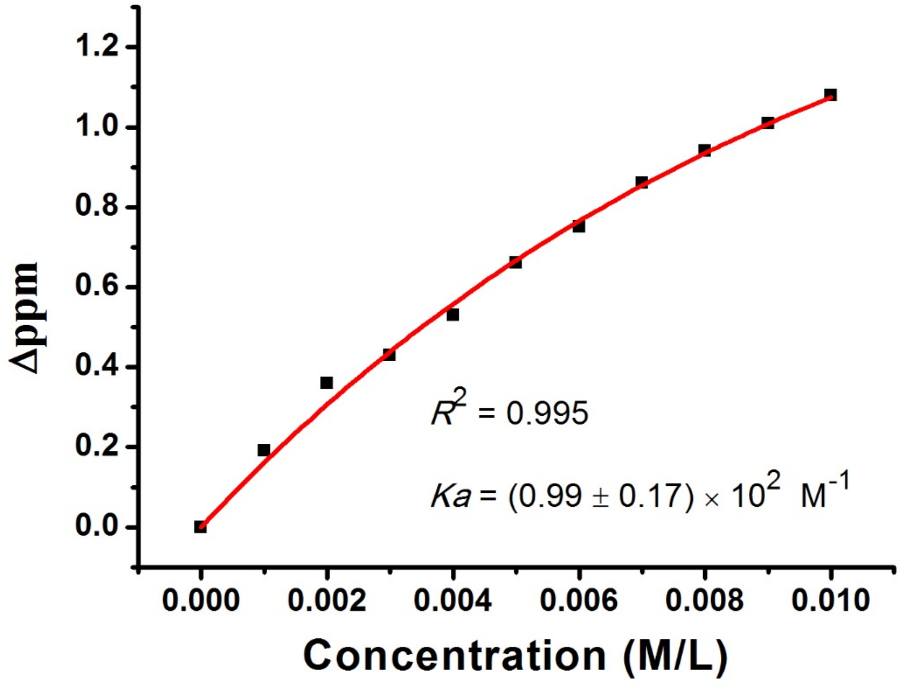

Figure S35. The chemical shift changes of H_a in n-butanol upon addition of **MSP5**. The red solid line was obtained from the non-linear curve-fitting method.

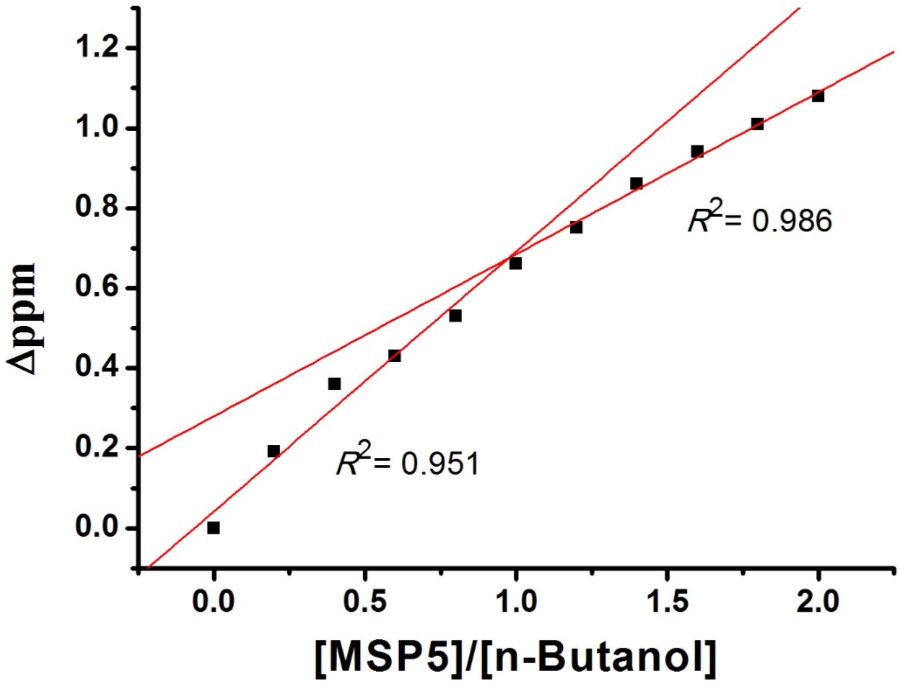

Figure S36. Mole ratio plot for the complexation between MSP5 and n-butanol, indicating a 1:1 stoichiometry.

(2) Pentanol ⊂ MSP5

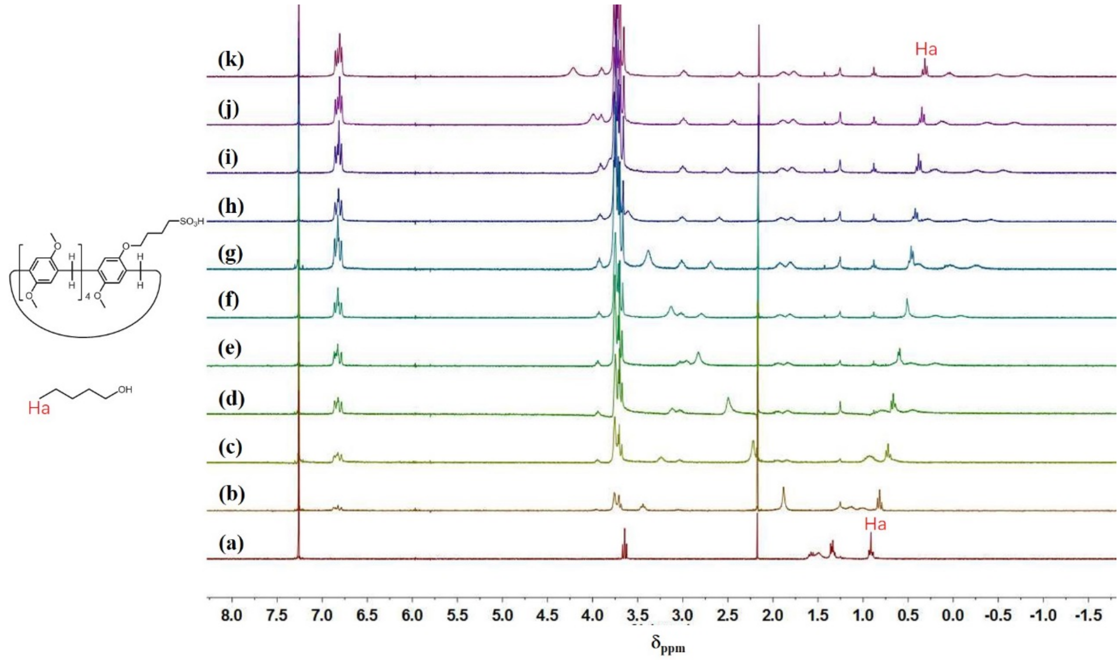

Figure S37. ^{1}H NMR titration (300 MHz, $CDCl_3$, 298 K) of n-pentanol at a concentration of 5.00 mM with different concentration (mM) of MSP5 in $CDCl_3$. (a) 0.00, (b) 1.00, (c) 2.00, (d) 3.00, (e) 4.00, (f) 5.00, (g) 6.00, (h) 7.00, (i) 8.00, (j) 9.00, and (k) 10.00.

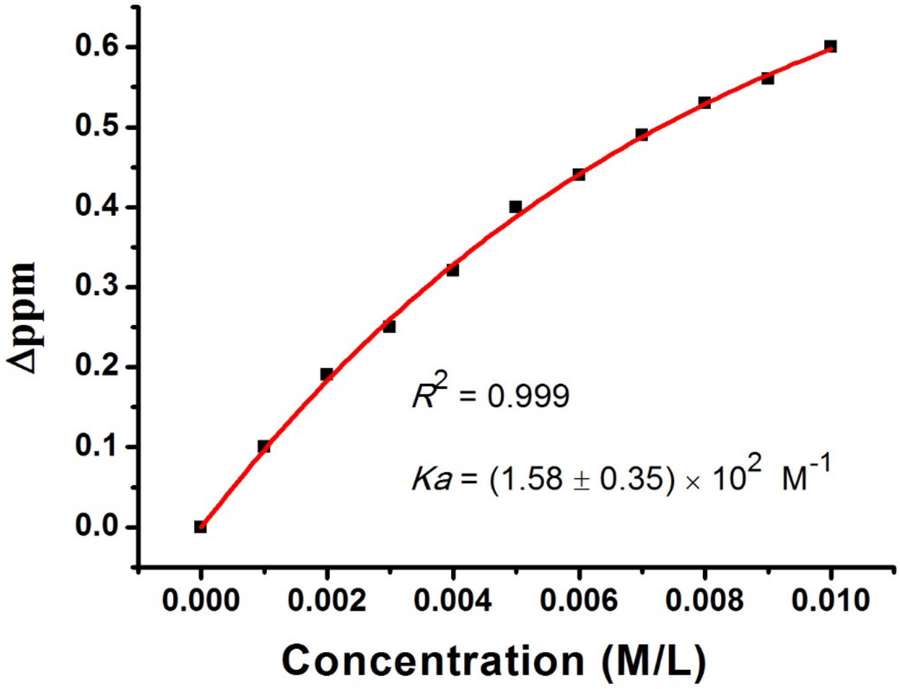

Figure S38. The chemical shift changes of H_a in n-pentanol upon addition of MSP5. The red solid line was obtained from the non-linear curve-fitting method.

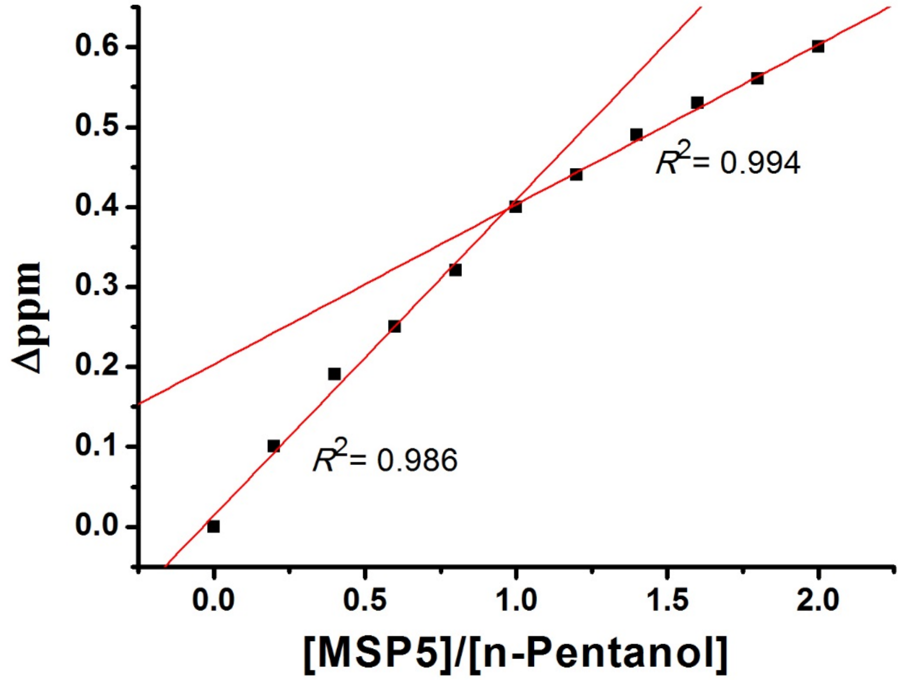

Figure S39. Mole ratio plot for the complexation between MSP5 and n-pentanol, indicating a 1:1 stoichiometry.

(3) n-Hexanol ⊂ MSP5

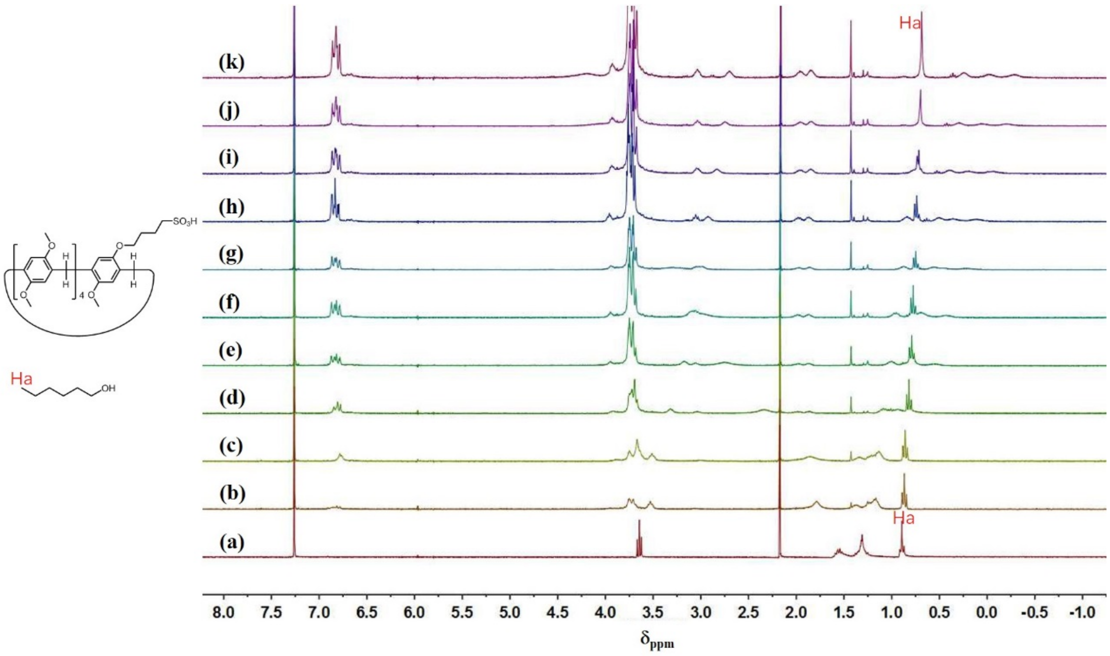

Figure S40. ^{1}H NMR titration (300 MHz, $CDCl_3$, 298 K) of n-hexanol at a concentration of 5.00 mM with different concentration (mM) of MSP5 in $CDCl_3$. (a) 0.00, (b) 1.00, (c) 2.00, (d) 3.00, (e) 4.00, (f) 5.00, (g) 6.00, (h) 7.00, (i) 8.00, (j) 9.00, and (k) 10.00.

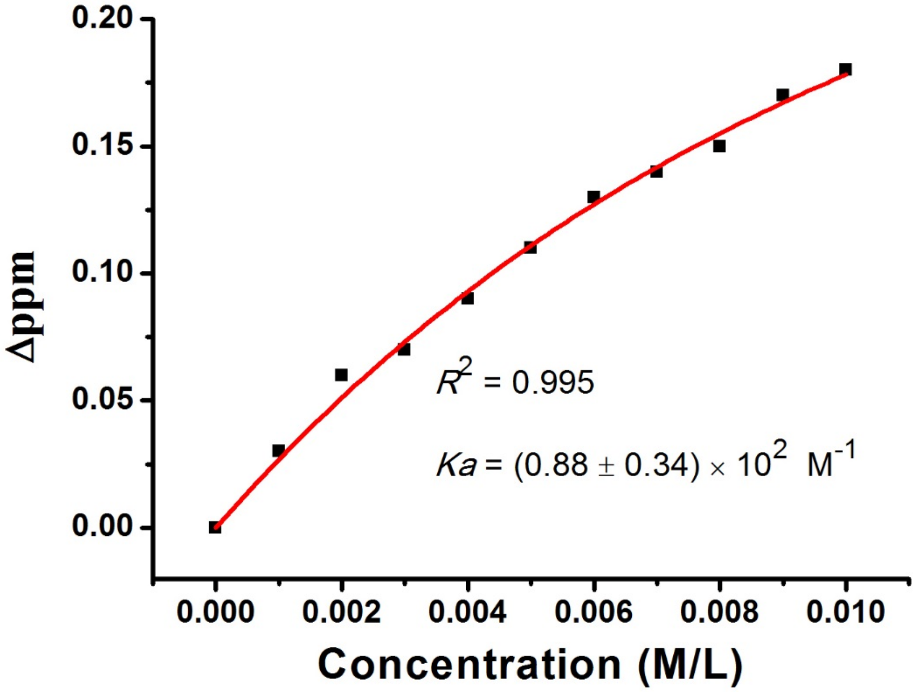

Figure S41. The chemical shift changes of H_a in n-hexanol upon addition of MSP5. The red solid line was obtained from the non-linear curve-fitting method.

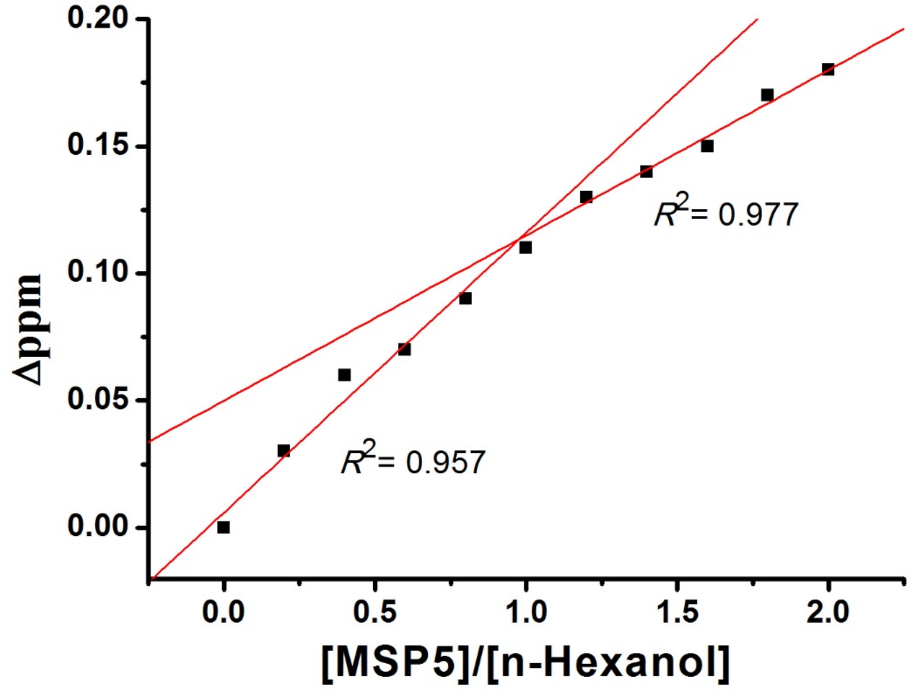

Figure S42. Mole ratio plot for the complexation between MSP5 and n-hexanol, indicating a 1:1 stoichiometry.

(4) 1,4-Butanediol ⊂ MSP5

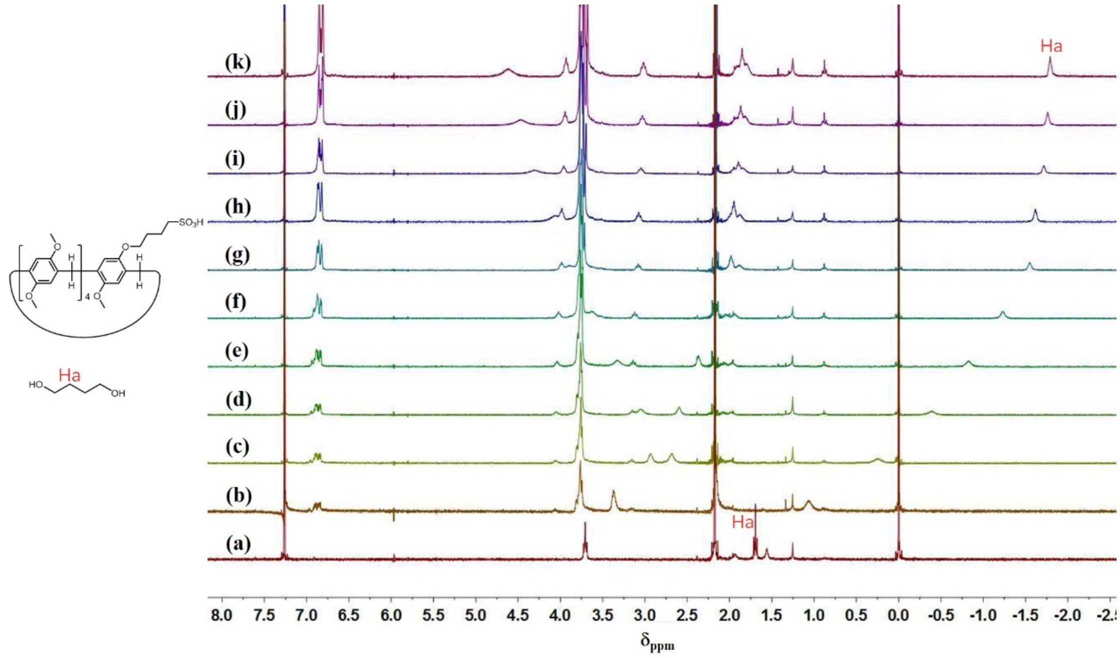

Figure S43. ^{1}H NMR titration (300 MHz, $CDCl_3$, 298 K) of 1,4-butanediol at a concentration of 5.00 mM with different concentration (mM) of MSP5 in $CDCl_3$. (a) 0.00, (b) 1.00, (c) 2.00, (d) 3.00, (e) 4.00, (f) 5.00, (g) 6.00, (h) 7.00, (i) 8.00, (j) 9.00, and (k) 10.00.

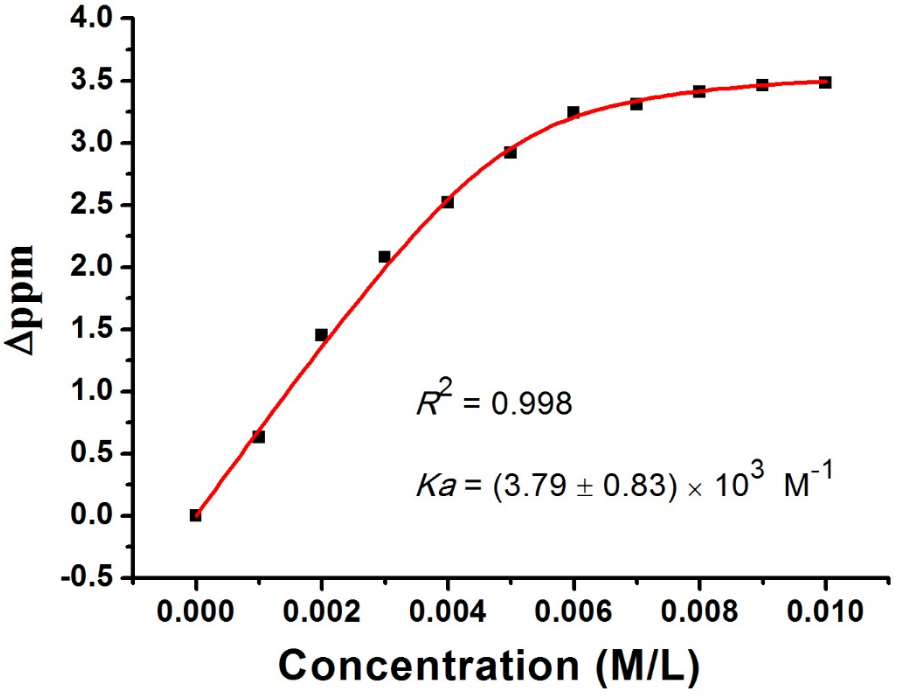

Figure S44. The chemical shift changes of H_a in 1,4-butanediol upon addition of MSP5. The red solid line was obtained from the non-linear curve-fitting method.

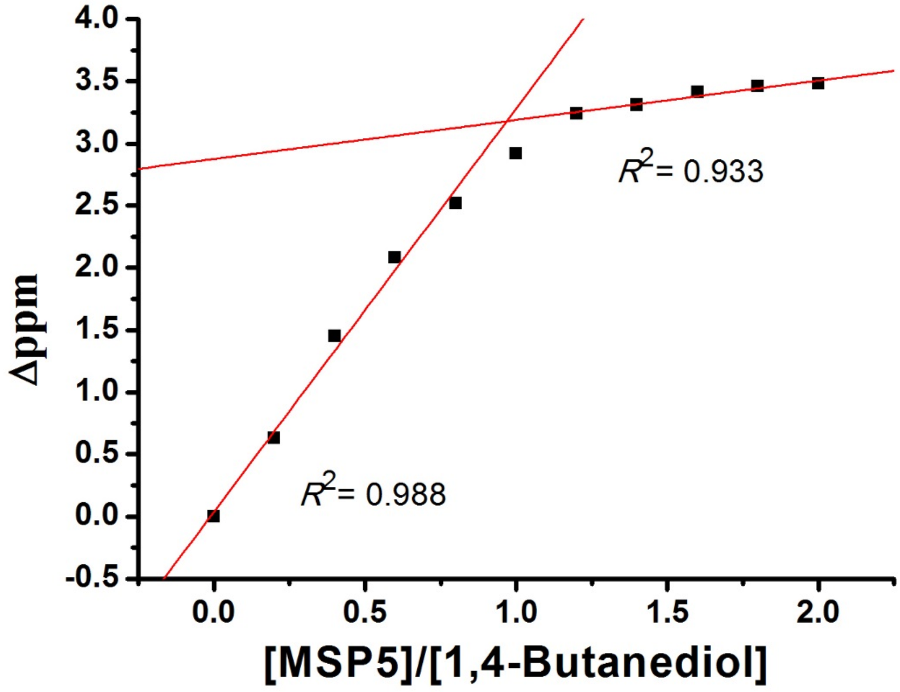

Figure S45. Mole ratio plot for the complexation between MSP5 and 1,4-butanediol, indicating a 1:1 stoichiometry.

(5) Pentanediol ⊂ MSP5

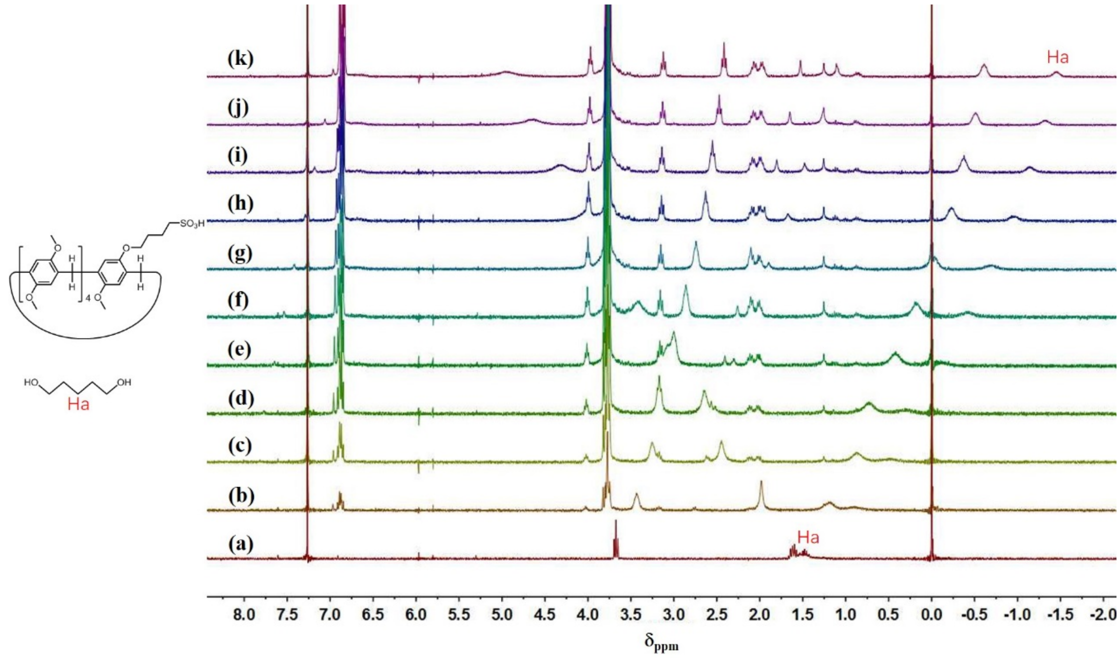

Figure S46. ^{1}H NMR titration (300 MHz, $CDCl_3$, 298 K) of pentanediol at a concentration of 5.00 mM with different concentration (mM) of MSP5 in $CDCl_3$. (a) 0.00, (b) 1.00, (c) 2.00, (d) 3.00, (e) 4.00, (f) 5.00, (g) 6.00, (h) 7.00, (i) 8.00, (j) 9.00, and (k) 10.00.

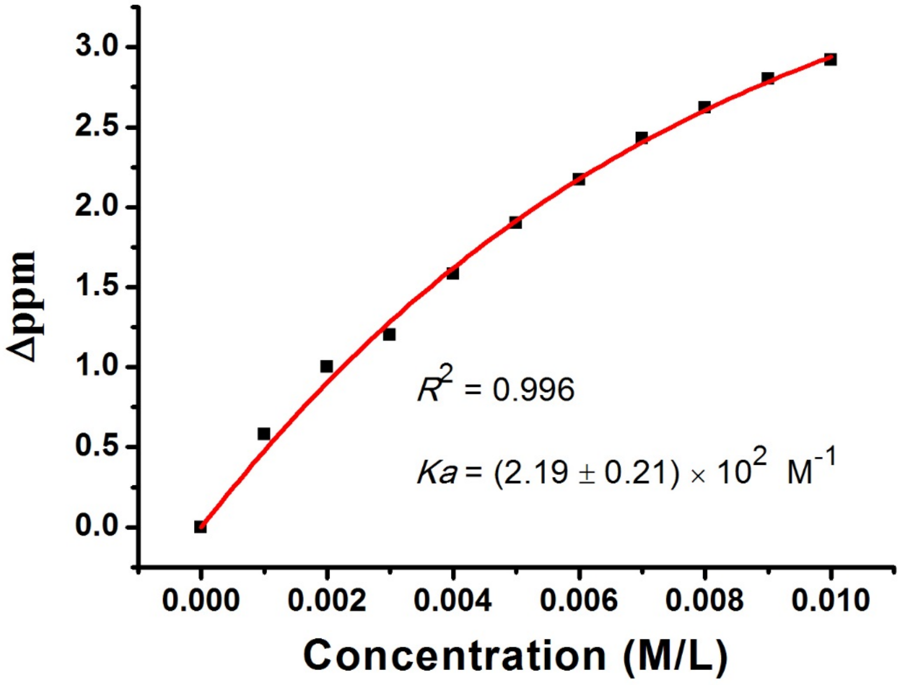

Figure S47. The chemical shift changes of H_a in pentanediol upon addition of MSP5. The red solid line was obtained from the non-linear curve-fitting method.

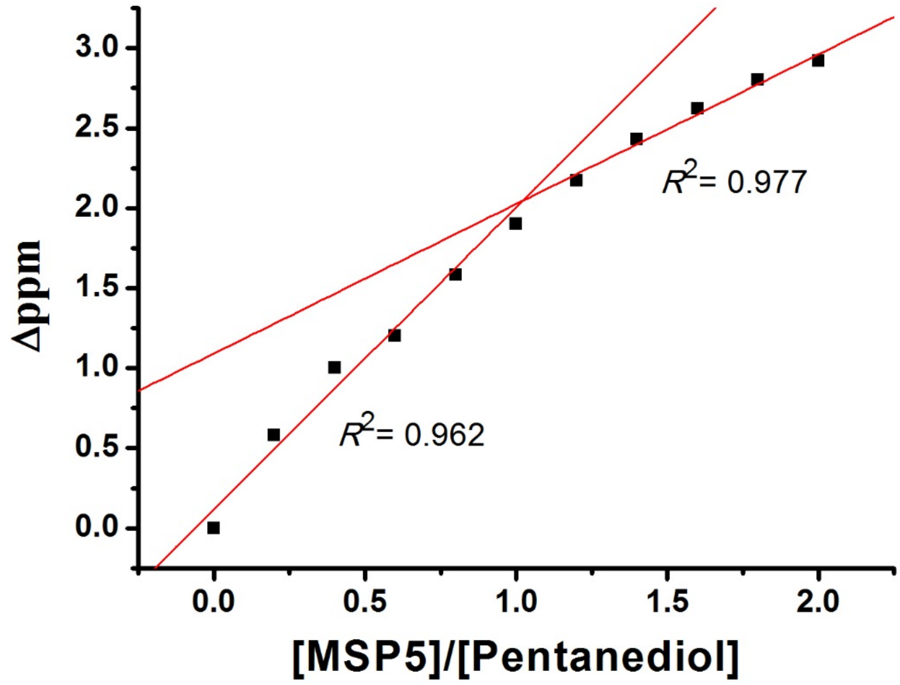

Figure S48. Mole ratio plot for the complexation between MSP5 and pentanediol, indicating a 1:1 stoichiometry.

(6) 1,6-Hexanediol ⊂ MSP5

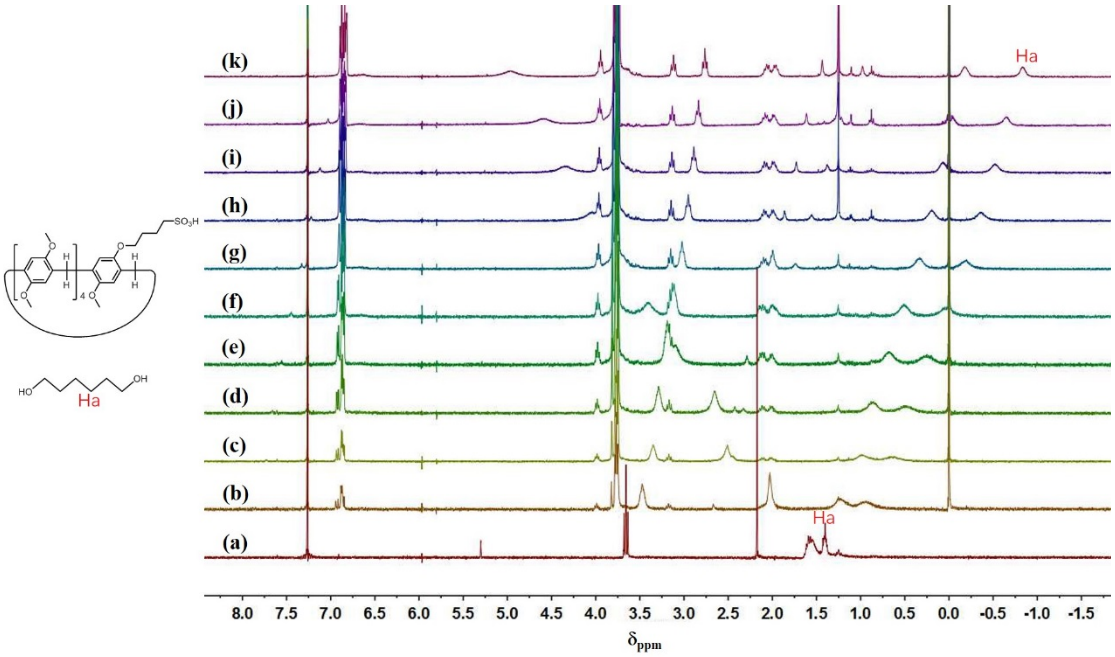

Figure S49. ^{1}H NMR titration (300 MHz, $CDCl_3$, 298 K) of 1,6-hexanediol at a concentration of 5.00 mM with different concentration (mM) of MSP5 in $CDCl_3$. (a) 0.00, (b) 1.00, (c) 2.00, (d) 3.00, (e) 4.00, (f) 5.00, (g) 6.00, (h) 7.00, (i) 8.00, (j) 9.00, and (k) 10.00.

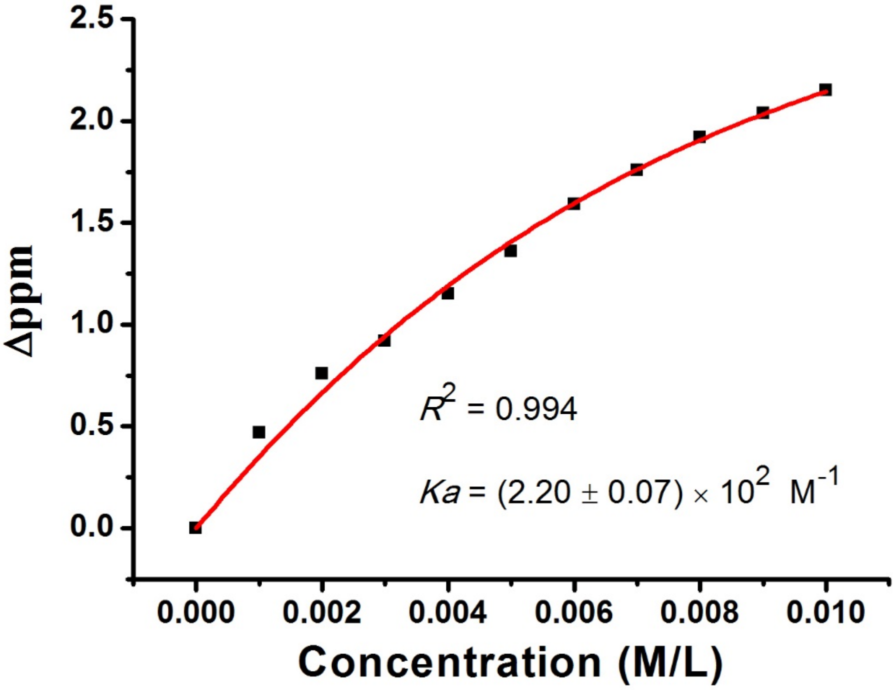

Figure S50. The chemical shift changes of H_a in 1,6-hexanediol upon addition of MSP5. The red solid line was obtained from the non-linear curve-fitting method.

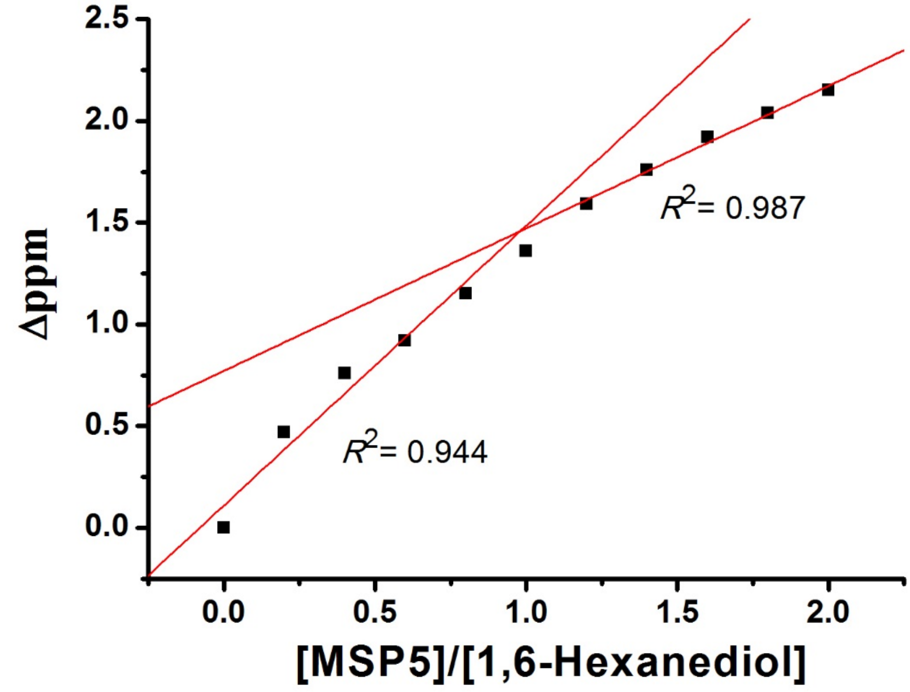

Figure S51. Mole ratio plot for the complexation between MSP5 and 1,6-hexanediol, indicating a 1:1 stoichiometry.

10. Association constant determination for G1 ⊂MSP5 in CHCl₃

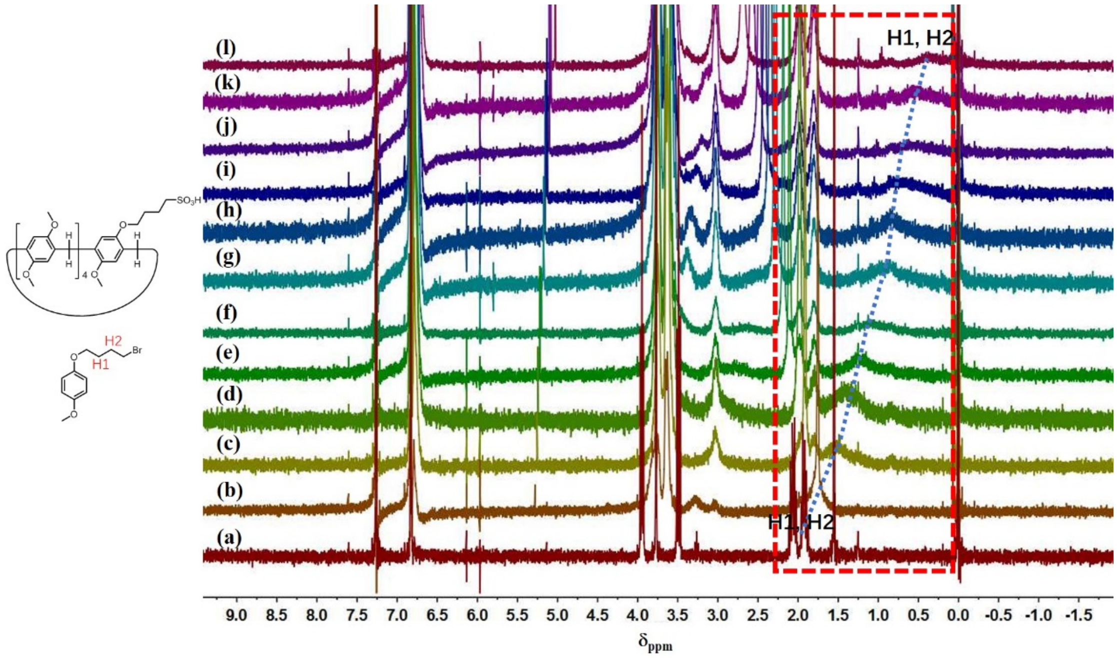

Figure S52. ^{1}H NMR titration (300 MHz, $CDCl_3$, 298 K) of G1 at a concentration of 5.00 mM with different concentration (mM) of MSP5 in $CDCl_3$. (a) 0.00, (b) 1.00, (c) 2.00, (d) 3.00, (e) 4.00, (f) 5.00, (g) 6.00, (h) 7.00, (i) 8.00, (j) 9.00, (k) 10.00, and (l) 12.00.

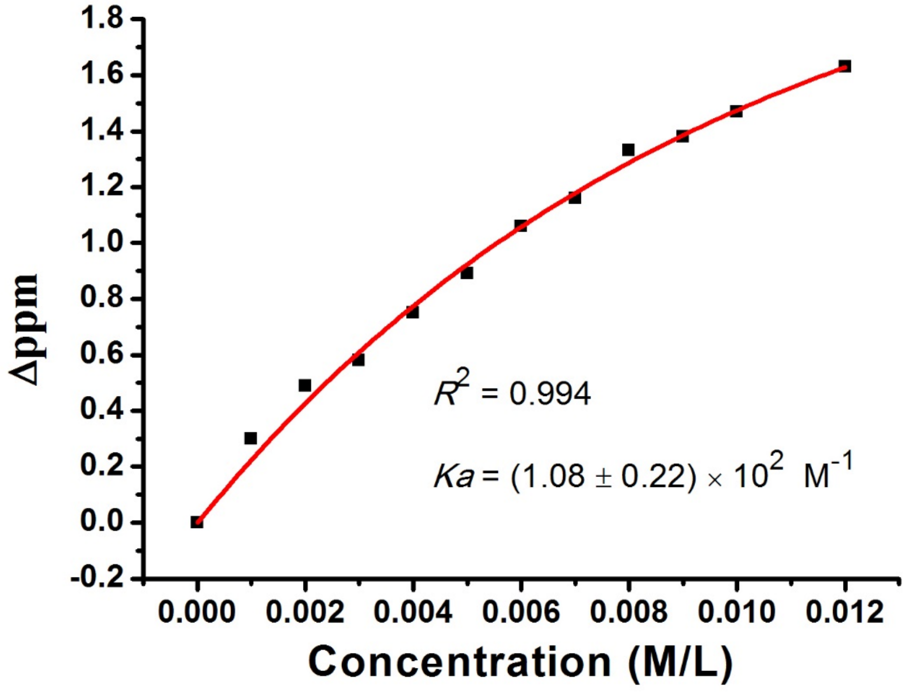

Figure S53. The chemical shift changes of H_a in G1 upon addition of MSP5. The red solid line was obtained from the non-linear curve-fitting method.

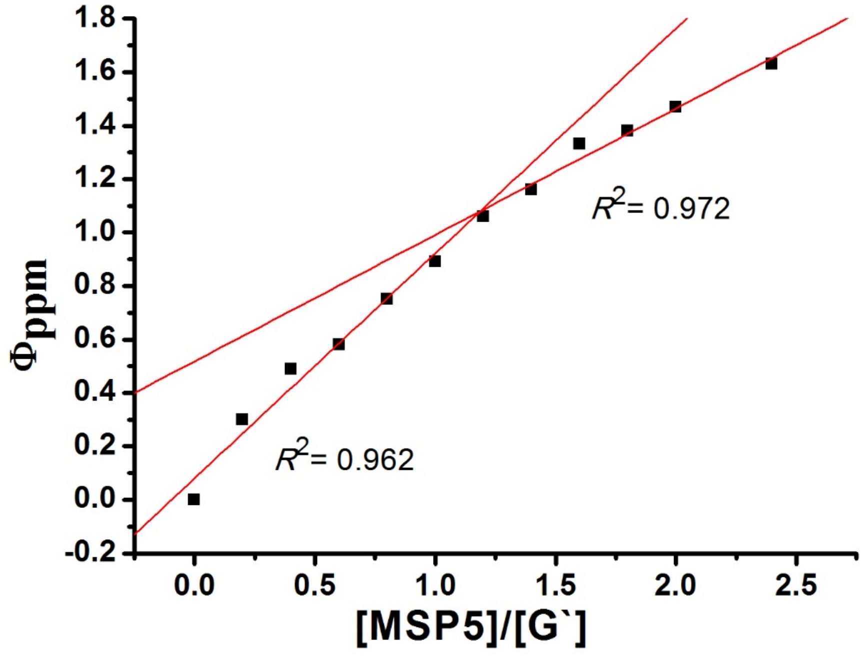

Figure S54. Mole ratio plot for the complexation between MSP5 and G1, indicating a 1:1 stoichiometry.

11. Association constant determination for G1 ⊂MCP5 in CHCl₃

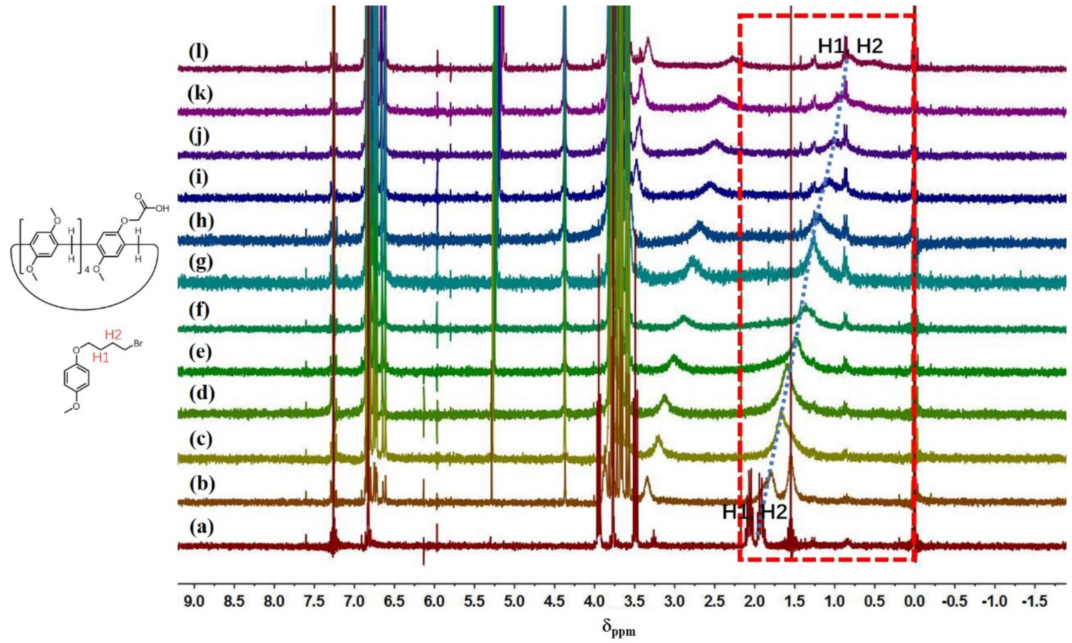

Fig. S55 ^{1}H NMR titration (300 MHz, $CDCl_3$, 298 K) of G1 at a concentration of 5.00 mM with different concentration (mM) of MCP5 in $CDCl_3$. (a) 0.00, (b) 1.00, (c) 2.00, (d) 3.00, (e) 4.00, (f) 5.00, (g) 6.00, (h) 7.00, (i) 8.00, (j) 9.00, (k) 10.00, and (l) 12.00.

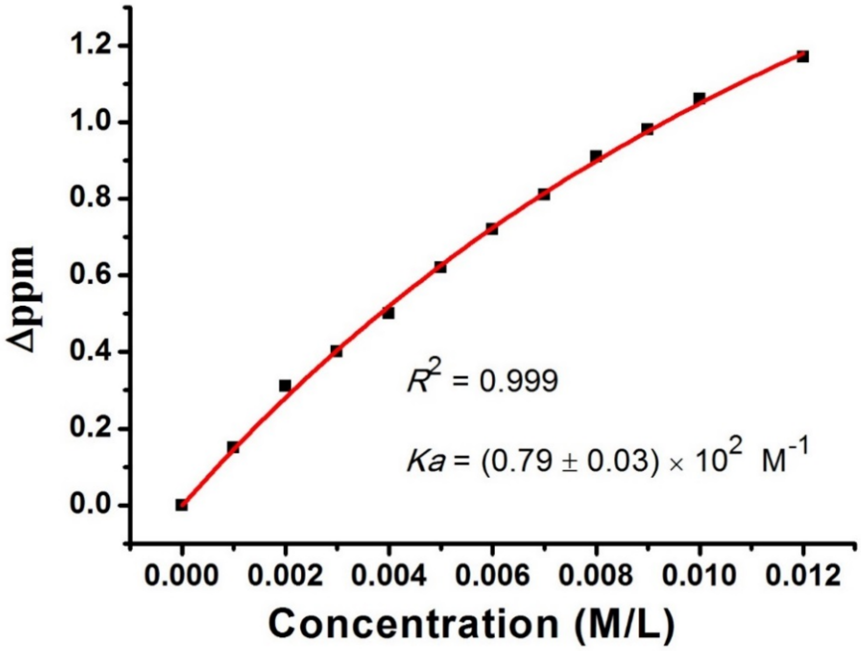

Figure S56. The chemical shift changes of H_a on G1 upon addition of MCP5. The red solid line was obtained from the non-linear curve-fitting method.

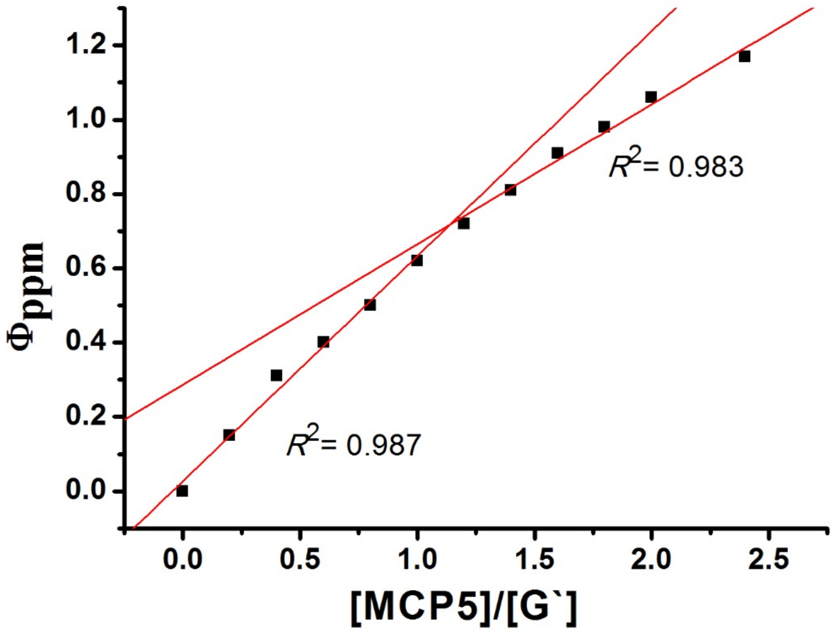

Figure S57. Mole ratio plot for the complexation between MCP5 and G1, indicating a 1:1 stoichiometry.

12. Host-guest interaction for TPE-(Br)$_4$$\subset$MSP5 in CHCl$_3$

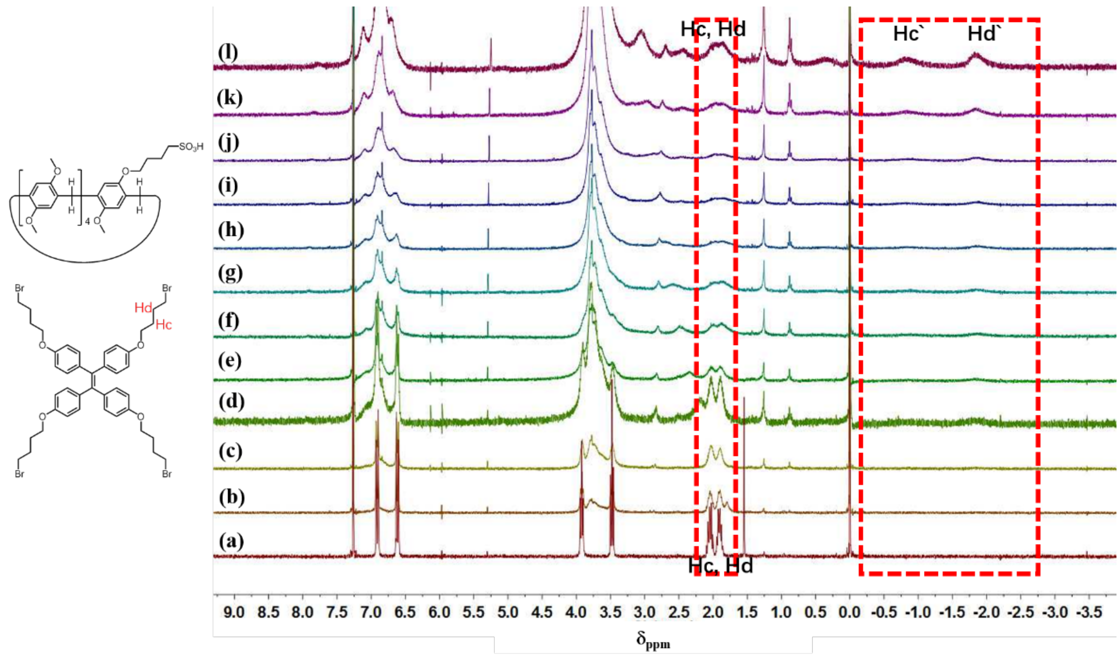

Figure S58. ^{1}H NMR titration (300 MHz, CDCl$_3$, 298 K) of TPE-(Br)$_4$ at a concentration of 5.00 mM with different concentration (mM) of MSP5 in CDCl$_3$. (a) 0.00, (b) 1.00, (c) 2.00, (d) 3.00, (e) 4.00, (f) 5.00, (g) 6.00, (h) 7.00, (i) 8.00, (j) 9.00, (k) 10.00, and (l) 12.00.

13. Host-guest interaction for TPE-$(Br)_4 \subset$ pillararene derivatives in $CHCl_3$

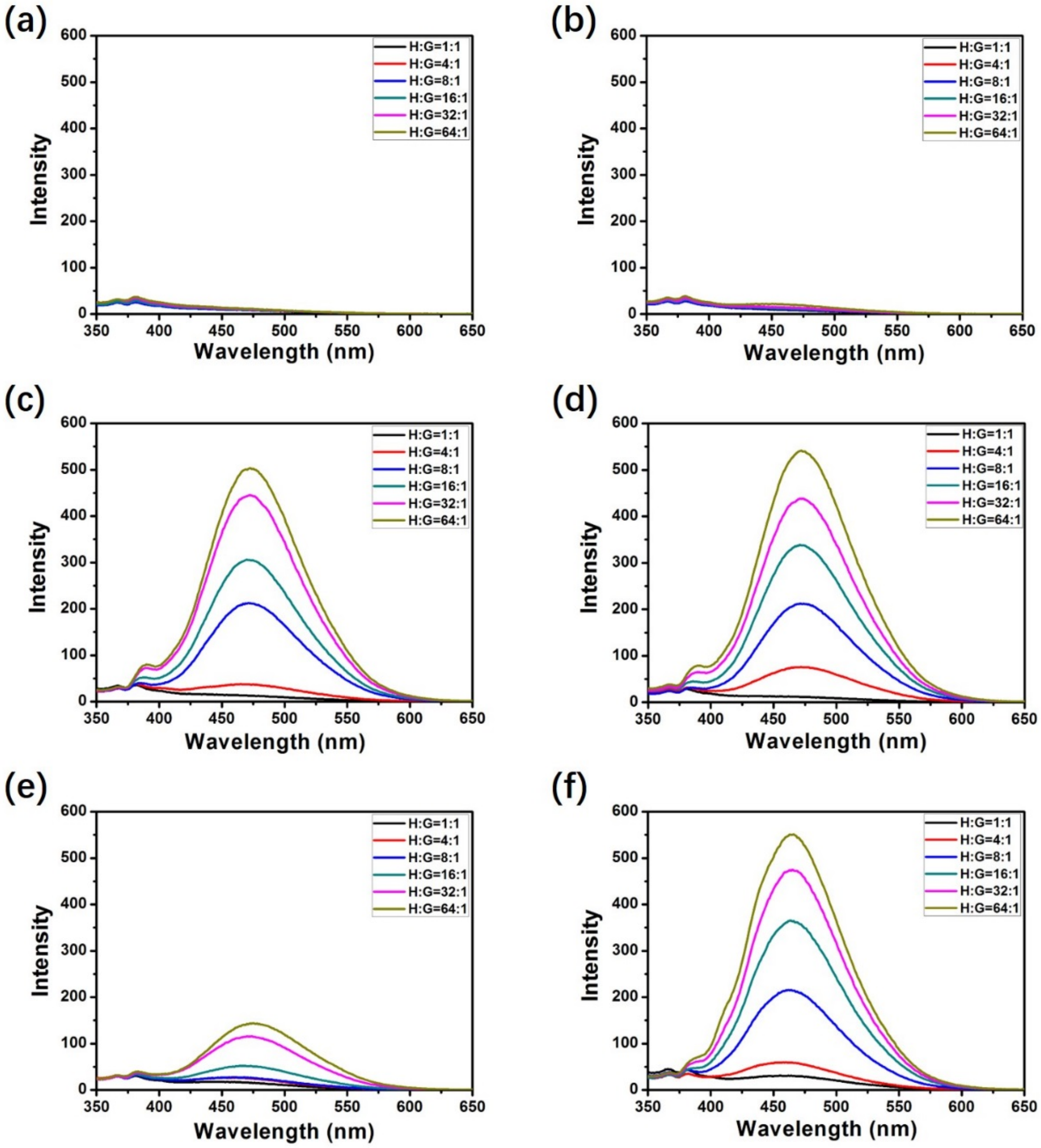

Figure S59. The fluorescence changes of upon addition of (a) MCP5, (b) monocarboxylatepillar[5]arene, (c) monosulfonicpillar[5]arene, (d) monosulfonatepillar[5]arene, (e) monophosphoricpillar[5]arene, (f) monophosphatepillar[5]arene in TPE-$(Br)_4$ solution.

14. DOSY NMR spectrum of TPE-(Br)$_4$⊂MSP5

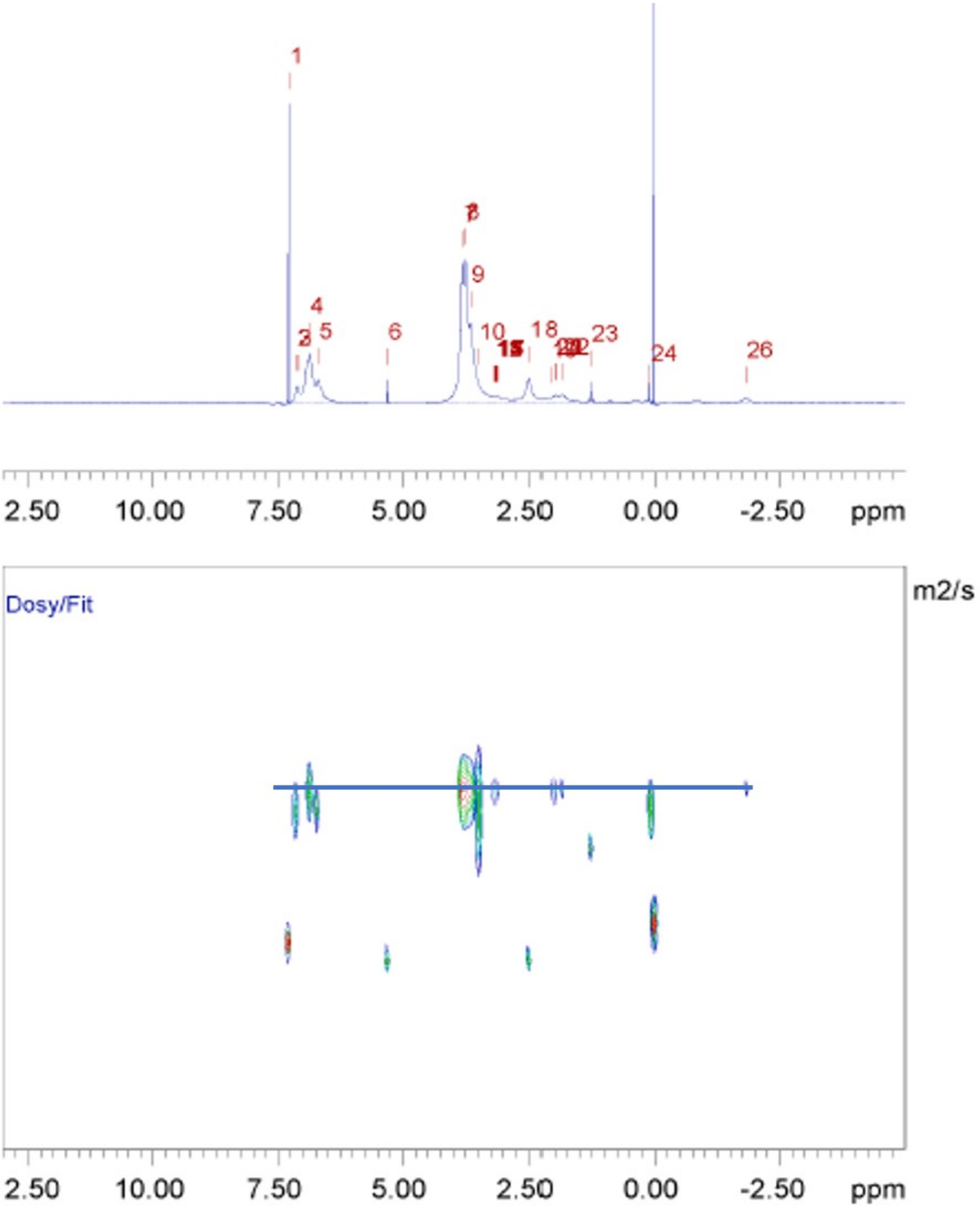

Figure S60. The DOSY NMR spectra of TPE-(Br)$_4$⊂ MSP5. (D = 2.6×10^{-9} m^2s^{-1})

15. SEM, Tyndall effect and DLS images of MSP5 and TPE-(Br)$_4$

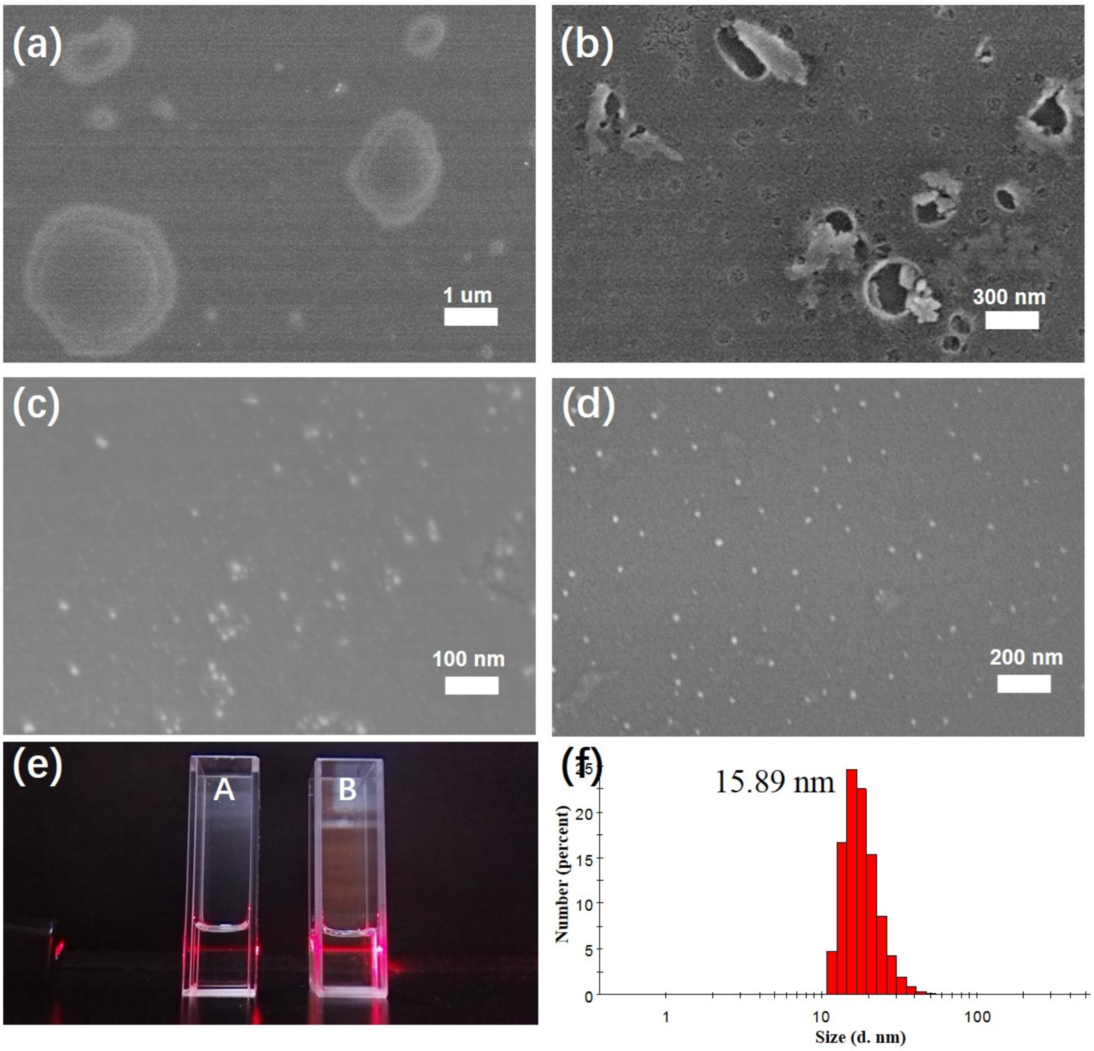

Figure S61. SEM images of (a) MSP5, (b) TPE-(Br)$_4$ and (c)(d) TPE-(Br)$_4$⊂MSP5. (e) The images of Tyndall effect of TPE-(Br)$_4$⊂MSP5 at different concentration: (A) concentration = 2×10^{-4} M, (B) concentration = 2×10^{-3} M; (f) The DLS spectrum of TPE-(Br)$_4$ ⊂ MSP5.

16. NMR spectra of MSP5 and ethylenediamine

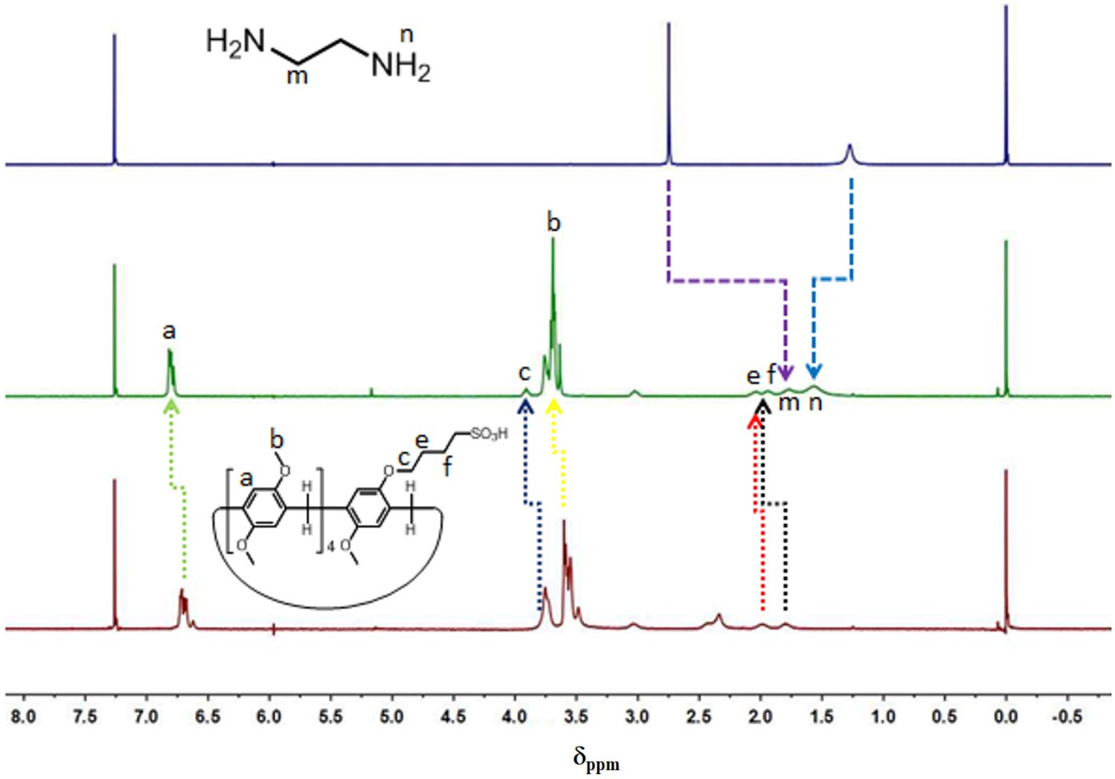

Figure S62. ^{1}H NMR experiment (300 MHz, $CDCl_3$, 298 K) of ethylenediamine at a concentration of 5.00 mM upon interacting with 5.00 mM MSP5 in $CDCl_3$.

17. Solid state fluorescence image and spectrum of host-guest complex

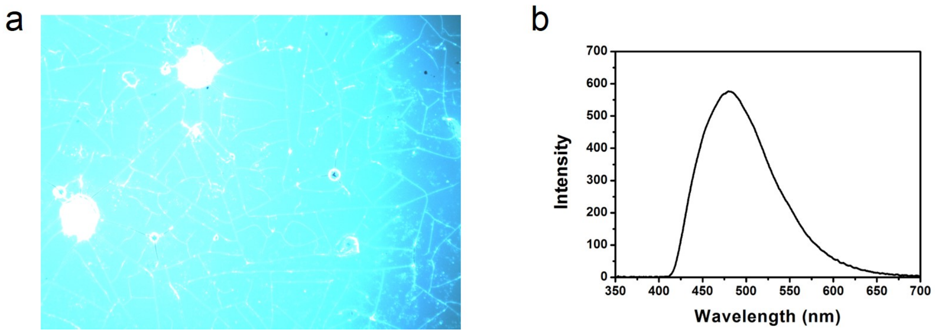

Figure S63. (a) Host-guest fluorescence complex solid state fluorescence image, (b) host-guest fluorescence complex solid state fluorescence spectrum.

References

S1. Dong, Y. *et al.* Investigating the effects of side chain length on the AIE properties of water-soluble TPE derivatives. *Tetrahedron Lett.*, **55**, 1496-1500 (2014).

S2. Dalvi-Malhotra, J. and Chen, L. Enhanced conjugated polymer fluorescence quenching by dipyridinium-based quenchers in the presence of surfactant. *J. Phys. Chem. B,* **109,** 3873-3878 (2005).

S3. Song, N. *et al.* Stimuli-responsive blue fluorescent supramolecular polymers based on a pillar[5]arene tetramer. *Chem.Commun.*, **50**, 8231-8234 (2014).

S4. Chen, Y. *et al.* Monoester copillar[5]arenes: synthesis, unusual self-inclusion behavior, and molecular recognition. *Chem.- Eur. J.*, **19**, 7064-7070 (2013).

S5. Chen, Y. *et al.* A monophosphoryl copillar[5]arene: synthesis and host–guest complexation with alkanols. *RSC Adv.*, **3**, 21405-21408 (2013).
